# Evaluation of functional group compatibility and development of reaction-accelerating additives in ammonium salt-accelerated hydrazinolysis of amides

**DOI:** 10.3389/fchem.2024.1378746

**Published:** 2024-05-22

**Authors:** Jeesoo Choi, Anna Nawachi, Natsuki Saito, Yuta Kondo, Hiroyuki Morimoto, Takashi Ohshima

**Affiliations:** ^1^ Graduate School of Pharmaceutical Sciences, Kyushu University, Fukuoka, Japan; ^2^ Department of Applied Chemistry, Graduate School of Engineering, Kyushu Institute of Technology, Fukuoka, Japan

**Keywords:** amide bond cleavage, functional group compatibility, functional group evaluation kit, carboxylic acid, zinc trifluoromethanesulfonate

## Abstract

Functional group compatibility in an amide bond cleavage reaction with hydrazine was evaluated for 26 functional groups in the functional group evaluation (FGE) kit. Accurate and rapid evaluation of the compatibility of functional groups, such as nitrogen-containing heterocycles important in drug discovery research, will enhance the application of this reaction in drug discovery research. These data will be used for predictive studies of organic synthesis methods based on machine learning. In addition, these studies led to discoveries such as the unexpected positive additive effects of carboxylic acids, indicating that the FGE kit can propel serendipitous discoveries.

## 1 Introduction

Amide bonds are among the most abundant chemical bonds in nature and are widely found in various organic molecules, such as peptides, natural products, and pharmaceuticals (Pattabiraman and Bode, 2011; Kaspar and Reichert, 2013; Brown and Bostrom, 2016). The chemical stability of amide bonds is extremely high due to their tendency to form resonance structures (Kemnitz and Loewen, 2007; Wang and Cao, 2011; Mahesh et al., 2018), and this stability affords beneficial properties to amide compounds. Due to their stability, except for biochemical cleavage by enzymes such as peptidases (Dai et al., 1995; Wu et al., 2020), chemical cleavage of amide bonds is quite difficult and often requires harsh reaction conditions (Thorner et al., 2000; Rashed et al., 2019; Lv et al., 2023). If amide bonds can be cleaved under mild conditions, the corresponding carboxylic acid equivalents and amines can be synthesized from various amides. Therefore, the development of amide bond cleavage reactions under mild conditions has attracted increased attention in recent years. Although several excellent reactions have been reported (Chaudhari and Gnanaprakasam, 2019; Li and Szostak, 2020), cleavage of common unactivated amides still requires the use of highly reactive metal catalysts, strict anhydrous conditions, and higher reaction temperatures.

**SCHEME 1:**
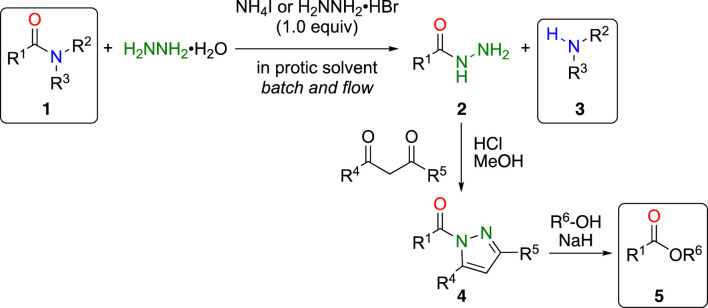
Ammonium salts promoted cleavage of amide bond with hydrazine and further transformation to esters.

To overcome these problems, we took advantage of the high nucleophilicity of hydrazine and found that simple inorganic ammonium salts, such as ammonium iodide, efficiently accelerated bond cleavage of unactivated amides **1** under mild conditions to afford the corresponding acyl hydrazides **2** and amines **3** in high yields (Scheme 1) (Shimizu et al., 2014). Furthermore, by employing a continuous microwave flow reactor, the reaction was easily scaled up (100 mmol scale, 23 mmol h^–1^) (Noshita et al., 2019). The obtained acyl hydrazides were easily converted into the corresponding ester **5** by reacting with β-diketone, such as acetylacetone, to the active amide acylpyrazole **4** (Kashima et al., 1994). By combining these reactions, both the carboxylic acid and amine portions of the amide **1** can be effectively utilized for further transformations.

If this reaction could be applied to amides with various functional groups, its usefulness would be greatly enhanced. The functional groups (FGs) are involved in the properties and reactivity of molecules and form the basis for organic chemistry and pharmaceutical chemistry (Dreos et al., 2011; Ertl, 2017). Although several functionalized amide substrates were investigated, comprehensive information on the functional group compatibility in this reaction has not yet been collected due to difficulties in synthesizing such functionalized amide substrates. Therefore, we used a functional group evaluation (FGE) kit (Saito et al., 2023), which allows for accurate and rapid assessment of information on the functional group compatibility using 26 FGE compounds, including the nitrogen-containing heterocycles imidazole and indole, important in drug discovery research (Collins and Glorius, 2013). In this system, the functional group compatibility of a given reaction is assessed by adding 26 external additives with various functional groups. Using this method, comprehensive data on functional group compatibility can be collected and entered into our “Digitization-driven Transformative Organic Synthesis (Digi-TOS)” database (https://en.digi-tos.jp). Artificial intelligence (AI) and digitization show great potential in next-generation organic synthesis, and data-driven prediction research of synthesis is essential to do so. In this regard, reliable information about functional group compatibility and chemoselectivity is important to understand the applicability of the reaction. This Digi-TOS database will be used for the development of a machine learning-based organic synthesis prediction method, such as a retrosynthetic analysis method (Mikulak-Klucznik et al., 2020; Zhang et al., 2023). For this purpose, it is essential to ensure the reliability of data, and therefore, statistical methods are used for handling the data. Another purpose of the FGE kit is to discover unexpected chemoselectivity and unexpected “positive” additive effects. Such unexpected discoveries, which are commonly referred to as “serendipitous,” frequently result in the development of reactions (Maegawa et al., 2011). In fact, in the present study, we found the unexpected positive additive effect of carboxylic acids, and further investigation allowed us to develop a new Lewis acid-catalyzed reaction system. Our results demonstrate that FGE kit effectively promotes serendipitous discovery.

## 2 Results and discussion

### 2.1 Evaluation of functional group compatibility using FGE kit

#### 2.1.1 Optimization of reaction conditions

Previously, we successfully developed ammonium salt-accelerated hydrazinolysis of unactivated amides using ammonium iodide and hydrazine monohydrate under mild conditions (Shimizu et al., 2014). To evaluate this reaction using the FGE kit, we selected *N*-(4-(trifluoromethoxy)phenyl)-4-(trifluoromethyl)benzamide (**1**) as the substrate because yields of the corresponding products 4-(trifluoromethyl)benzohydrazide (**2**) and 4-(trifluoromethoxy)aniline (**3**) can be determined by ^19^F NMR analysis of the crude mixture without affecting ^1^H-based contaminants such as additives in the FGE kit.

We first optimized reaction conditions for the hydrazinolysis of amide **1aa** using General Procedure A (Table 1) (See Supplementary Material for detailed information). Under the standard conditions of our previous studies using 10 equiv of hydrazine hydrate and 1.0 equiv of ammonium iodide at 70 °C for 48 h, the reaction gave only 20% of benzohydrazide **2a** (Entry 1). The reaction in the absence of solvent did not proceed (Entry 2). Increasing the reaction temperature to 100 °C increased the yield of **2a** to 85% in 24 h (Entry 3). Aiming to accelerate the reaction, we performed the reaction using acidic solvent hexafluoroisopropanol (Zhang et al., 2018; Kabi et al., 2020; Bhattacharya et al., 2021) and trifluoroethanol (Entries 4 and 5) (Smithrud et al., 1990; Bautista et al., 1999; Bai et al., 2019); the use of trifluoroethanol led to desired product **2a** in 93% yield. Using trifluoroethanol as the solvent in the absence of ammonium iodide decreased the reaction rate, however, clearly indicating that ammonium salt is important for achieving high reactivity (Entry 6) (Shimizu et al., 2012). On the other hand, the addition of 2 equiv of ammonium iodide decreased the yield of the product (Entry 7). NH_4_OAc (Kumar et al., 2022) instead of NH_4_I resulted in the lower yield of **2a** (Entry 8). When hydrazine monohydrate was not added, the reaction did not proceed and amide **1aa** was recovered quantitatively (Entry 9). Although increasing the amount of hydrazine from 10 to 20 equiv. improved the yield (Entry 10), the change was not significant (Entry 5). On the other hand, two equivalents of hydrazine gave **2a** in 14% yield (Entry 11); therefore, 10 equiv was considered optimal. The concentration of the solvent also affected the reaction (Entries 12–14), and a 1 M solvent concentration was optimal (Entry 5). As shown in this table, the yields of benzohydrazide **2a** and aniline **3a** were nearly identical, so only the yields of **2a** are listed in the following tables (See Table 1).

**TABLE 1:** Optimization of reaction condition using amide **1aa**.

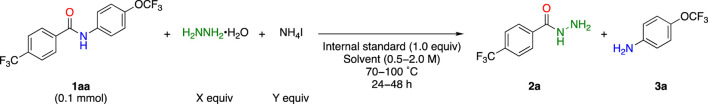									
Entry	X equiv	Y equiv	Solvent	(M)	Temp. (°C)	Time (h)	Yield (%)^a^		
							2a	3a	1aa
1	10	1.0	ethanol	1.0	70	48	20	17	82
2	10	1.0	−	−	70	24	N.D^b^	N.D^b^	≥99
3	10	1.0	ethanol	1.0	100	24	85	81	17
4	10	1.0	hexafluoroisopropanol	1.0	100	24	82	82	18
5	10	1.0	trifluoroethanol	1.0	100	24	93	93	7
6	10	−	trifluoroethanol	1.0	100	24	52	53	48
7	10	2.0	trifluoroethanol	1.0	100	24	69	67	32
8	10	1.0^c^	trifluoroethanol	1.0	100	24	57	60	42
9	−	1.0	trifluoroethanol	1.0	100	24	N.D^b^	N.D^b^	≥99
10	20	1.0	trifluoroethanol	1.0	100	24	≥99	≥99	N.D^b^
11	2.0	1.0	trifluoroethanol	1.0	100	24	15	14	85
12	10	1.0	trifluoroethanol	0.5	100	24	66	67	33
13	10	1.0	trifluoroethanol	1.4	100	24	78	78	22
14	10	1.0	trifluoroethanol	2.0	100	24	76	76	24

^a^ Determined by ^19^F NMR analysis of the crude mixture using 4-(trifluoromethoxy)anisole (0.1 mmol) as an internal standard.

^b^ Not detected.

^c^ NH_4_OAc was used instead of NH_4_I.

#### 2.1.2 Additive compounds A0–A26 for FGE kit

After determining the optimized reaction conditions (Table 1, Entry 5), we applied the FGE kit to the reaction. Additive compounds **A0**–**A26** for our FGE kit contain the 4-chlorophenyl moiety as the parent backbone to facilitate ^1^H NMR monitoring of the remaining additives (See Table 2). Because of its UV absorption, the 4-chlorophenyl moiety also enables HPLC analysis of the remaining additives. Furthermore, the isotopic distribution of the chlorine atoms facilitates the detection of chlorine-containing additive molecules as well as side products associated with the additives using mass spectroscopic analysis. We selected 26 functional groups found in many organic molecules, including amino acid residues such as the nitrogen-containing heterocycles imidazole **A7** and indole **A22**.

**TABLE 2:** Experimental results for hydrazinolysis of amides using FGE kit.

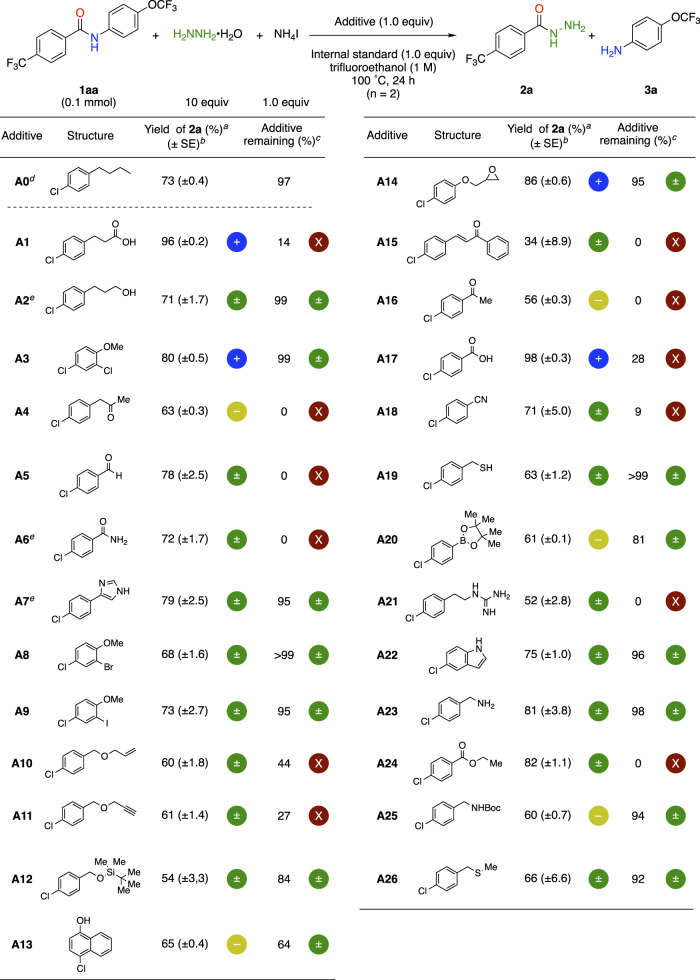			

^a^ Determined by ^19^F NMR analysis of the crude mixture using 4-(trifluoromethoxy)anisole (0.1 mmol) as an internal standard.

^b^ Standard error.

^c^ Determined by ^1^H NMR analysis of the mixture using 4-(trifluoromethoxy)anisole (0.1 mmol) as an internal standard.

^d^ n = 5.

^e^ n = 4.

#### 2.1.3 Control experiment with additive A0

Before starting the evaluation with the FGE kit, it was necessary to check whether the 4-chlorophenyl structure in the additive affects the reaction. Therefore, a control experiment was carried out by adding 1.0 equiv of additive **A0** (1-butyl-4-chlorobenzene), which does not contain an additional functional group, to the amide bond cleavage reaction. Another objective of the control experiment with additive **A0** was to check for reproducibility using the criterion of the standard deviation (σ) of the product yield (%) within 5 (σ ≤ 5) in five experiments (n = 5). Detailed experimental methods for using the FGE kit are reported in our previous work (Saito et al., 2023) (See Supplementary Material for detailed information).

**FIGURE 1:**
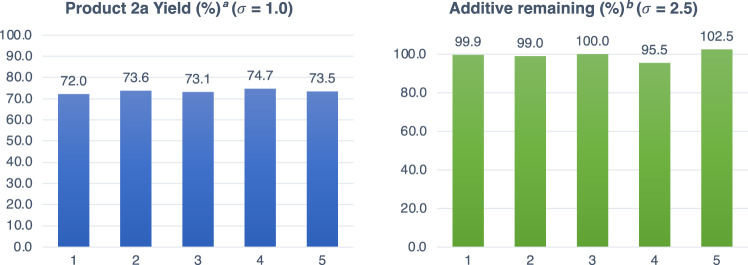
Reaction with additive **A0**. ^a^Determined by ^19^F NMR analysis of the crude mixture using 4-(trifluoromethoxy)anisole (0.1 mmol) as an internal standard. ^b^The amount of additives were calculated by ^1^H NMR analysis of the crude mixture using 4-(trifluoromethoxy)anisole (0.1 mmol) as an internal standard.

Under optimized reaction conditions, the reaction was carried out five times with 1.0 equiv of additive **A0** (Figure 1). The average yield decreased to 73% due to the decrease in the concentration caused by the addition of **A0**. Therefore, evaluation of the additive effect using **A1**–**A26** is based on this yield. The standard deviation of the yield (%) was 0.97, suggesting that sufficient reproducibility could be obtained even under the conditions with additives. No decreases in the remaining additive (%) were observed (97%), and high reproducibility was obtained with a standard deviation of 2.52, indicating that the 4-chlorophenyl structure is tolerant to the reaction conditions (Supplementary Table S1).

#### 2.1.4 Evaluation functional group compatibility with additive A1–A26

**FIGURE 2:**
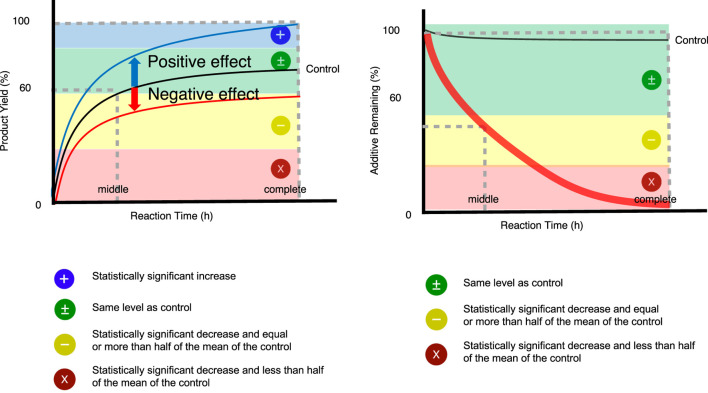
The symbols in FGE kit.

After fulfilling the reproducibility criteria of additive **A0**, we examined the additive effects of the other additives, **A1**–**A26**, in the FGE kit. Each additive was subjected to an F-test through duplicate experiments (n = 2). Only when the F-test indicated that the variance differed and the variability was too high, two additional experiments with the same additives were performed. The following symbols are used in the experimental results (Figure 2; Table 2). The blue plus sign (+) indicates a statistically significant increase effect. The green plus/minus sign (±) indicates no statistically significant effect, but a result that was comparable to that of the control experiment. The yellow dash sign (−) indicates a statistically significantly decrease effect. When there was a significant decrease of more than half compared with the control experiment, a red “X” was used. Significant differences were determined by a t-test.

The effects of 26 additives **A1**–**A26** in the FGE kit were examined using General Procedure B (Table 2) (See Supplementary Material). Additives **A2**, **A7**–**A9**, **A12**, **A19**, **A22**, **A23**, and **A26** with alcohol, imidazole, aryl bromide, iodide, silyl ether, thiol, indole, primary amine, thioester moieties had no effect on product yield nor remaining additives. It is particularly important that highly reactive thiol **A19** and thioether **A26**, as well as *N*-unprotected imidazole **A7** and indole **A22**, were tolerated. Additives **A5**, **A6**, **A10**, **A11**, **A15**, **A18**, **A21** and **A24** with aldehyde, primary amine, terminal alkene, alkyne, enone, nitrile, guanidine and ester moieties did not affect the product yields, but the remaining additives were decreased, suggesting that the functional groups of these additives reacted under the reaction conditions (*vide infra*). In the case of additives **A3** and **A14** with aryl chloride and epoxide moieties, the yield of the product slightly increased and the remaining additive was unchanged. On the other hand, additives **A1** and **A17** with carboxylic acid moieties provided significantly higher product yields than the control reaction with additive **A0** although the remaining additives were decreased due to the formation of byproducts (*vide infra*). For additives **A4**, **A13** and **A16** with alkyl ketone, naphthol and aryl ketone moieties, both the yield of the product and the remaining additive were decreased. The addition of **A20** and **A25** with Bpin and Boc-protected amine moieties decreased the product yields but did not change the remaining additives.

The decrease in the remaining additives was mainly due to the formation of the corresponding byproducts by a competitive reaction between the additives and hydrazine. The byproducts that formed from the additives were either isolated from the crude mixture or, if difficult to isolate, synthesized by methods reported in the literature, and their structures were confirmed using NMR and mass spectrometry analysis (See Supplementary Material).

As expected, carboxyl groups in additives **A1** and **A17**, the amide group in additive **A6**, and the ester group in additive **A24** reacted with hydrazine to afford the corresponding acyl hydrazides such as **B1**.

**SCHEME 2:**
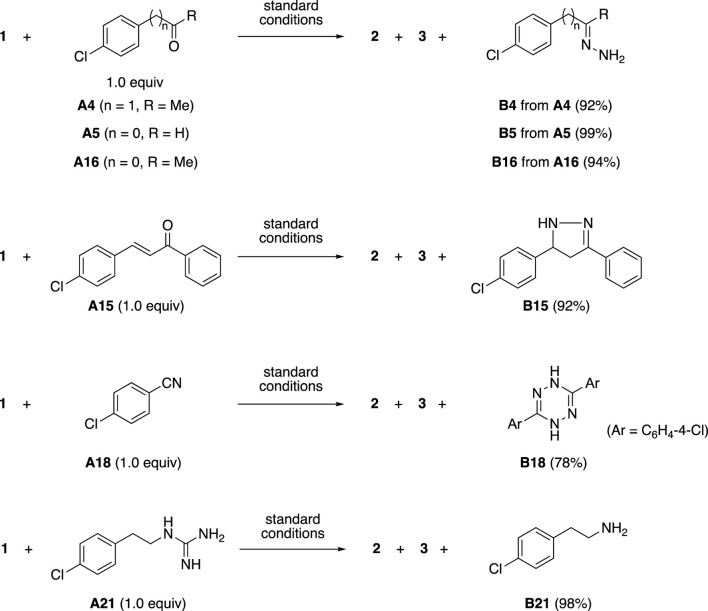
Formation of byproducts derived from additives **A4**, **A5**, **A15**, **A16**, **A18** and **A21**.

The carbonyl groups in additive **A4**, **A5**, and **A16** also reacted with hydrazine to form the corresponding hydrazones such as **B4**, **B5**, and **B16** (Scheme 2). Additive **A15** with an enone moiety and additive **A18** with a nitrile moiety also reacted with hydrazine to give nitrogen-containing heterocycles pyrazoline **B15** and tetrazine **B18** in good yields. On the other hand, some unexpected reactions were also observed. Additive **A21** with a guanidine moiety reacted with hydrazine to give amine **B21**.

**SCHEME 3:**
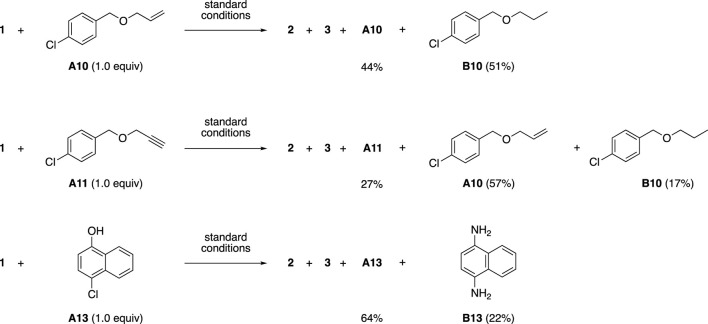
Formation of byproducts derived from additives **A10**, **A11**, and **A13**.

In the reactions using additives **A10** and **A11** containing terminal alkene and alkyne, a partial reduction proceeded to produce alkane **B10** and alkene **A10** (Scheme 3). This type of reduction of olefins by hydrazine was reported by Imada et al., where hydrazine was in situ-oxidized to diimide and the reduction was promoted by Brønsted acids (Arakawa et al., 2017). Surprisingly, the phenolic hydroxyl group and Ar-Cl moiety in additive **A13** were converted to amino groups to give naphthalene-1,4-diamine (**B13**). The discovery of these unexpected reactions is an advantage of using the FGE kit.

**FIGURE 3:**
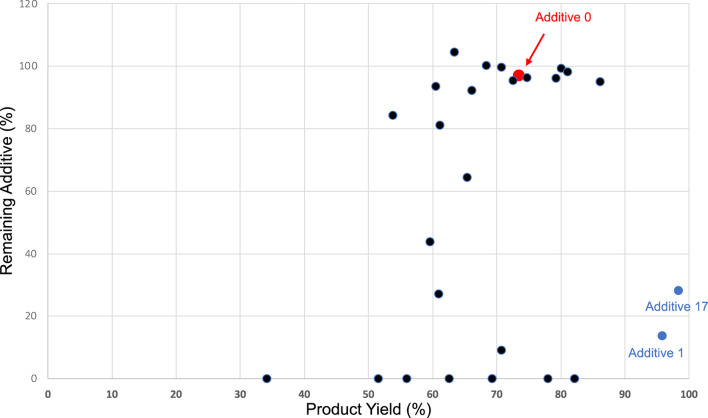
Two-dimensional representations of the product yield (%) and remaining additives (%).

Functional group compatibility of a reaction is evaluated from two aspects: the effect of the functionalized additive on the product yield and the remaining additive. The two-dimensional plot in Figure 3 shows the degree of the functional group compatibility for each reaction at a glance. In this plot, dots positioned near additive **A0** (red dot in the plot) indicate that the functional groups in the corresponding additive are tolerant in this reaction. On the other hand, dots father away from additive **A0** suggest that the reaction is significantly (often negatively) affected by the presence of the functional groups in the additives. This plot clearly shows that under the conditions of this amide bond cleavage reaction, although some functional groups reacted (reducing the recovery of the additive), many functional groups did not inhibit the reaction itself (reducing the yield). In addition, there are many additives with positive effects, and additives **A1** and **A17** with a carboxyl group significantly increased the product yield.

### 2.2 Acidic additives for accelerating the amide bond cleavage reaction

Because the above results suggested that carboxyl groups accelerate the ammonium salt-promoted amide bond cleavage reaction, we next examined various acidic additives.

#### 2.2.1 Screening of Brønsted acid additives

Carboxylic acids are easy to handle and often inexpensive (Gooßen et al., 2008; Pichette Drapeau and Gooßen, 2016). Therefore, even if the amide bond cleavage reaction requires an equivalent amount of additive, the carboxylic acid addition conditions can be a useful reaction system.

First, we screened various inorganic and organic Brønsted acids as additives using General Procedure B (See Supplementary Material). To evaluate the additive effects on the reaction rate, product yields were examined for 6 h during the course of the reaction. The use of inorganic acids **C1**–**C3** significantly decelerated the reactions (Table 3). Next, we examined a wide variety of mono-carboxylic acids **C4**–**C34** including **A1** and **A17** as additives to evaluate changes in the product yield. Overall, aliphatic or aromatic carboxylic acids were not very important, and the correlation between the electrical effects and reactivity was low. On the other hand, it is important to have a certain molecular size, and carboxylic acids, especially those with aromatic rings such as benzene rings, exhibited a good tendency. *Ortho*-substituted Benzoic acids **C16**–**C18** and **C26** were expected to have excellent effects because their carboxylic acid moieties did not react with hydrazine due to steric hindrance. They, however, did not significantly impact the product yields, suggesting that carboxylic acids without steric hindrance should be considered as additives to accelerate the reaction. The acceleration effect of heterocyclic rings is low (**C29**–**C34**). The presence of hydroxyl (**C8** and **C22**) and amino (**C7**) groups in the vicinity of carboxylic acids was not desirable, and dicarboxylic and tricarboxylic acids (**C34**–**C42**) rather inhibited the reaction. Other types of acidic compounds, 4-toluenesulfonic acid (**C41**) and BINOL (**C42**), were also ineffective. Based on this screening study, we concluded that 4-butyl benzoic acid (**C19**) and 2-naphthoic acid (**C27**) were the most effective additives for the amide bond cleavage reaction.

**TABLE 3:** Screening of Brønsted acid additives.

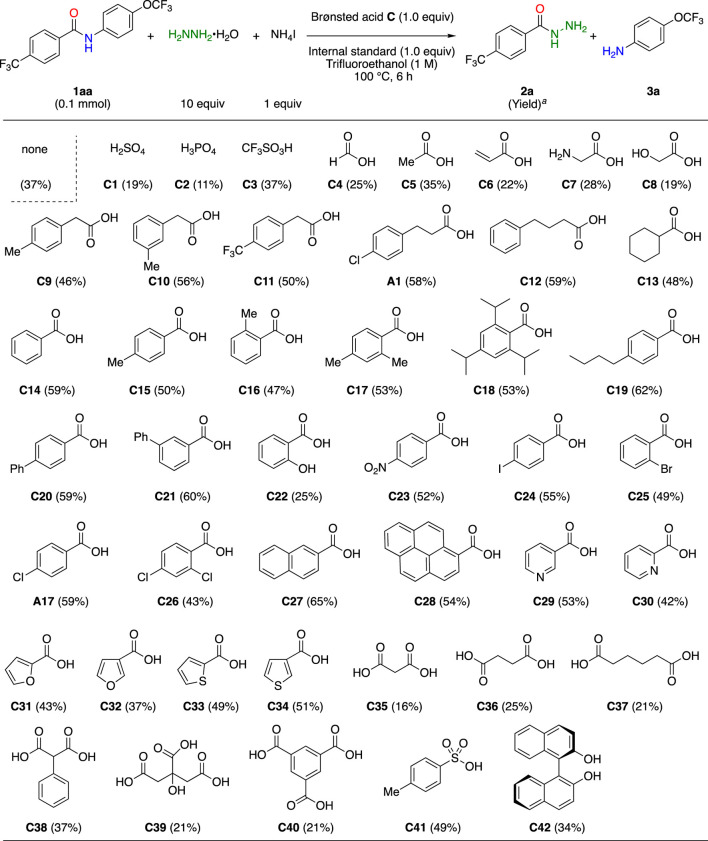								

^a^ Determined by ^19^F NMR analysis of the mixture using 4-(trifluoromethoxy)anisole (0.1 mmol) as an internal standard.

#### 2.2.2 Control experiments between carboxylic acid and hydrazide

**SCHEME 4:**
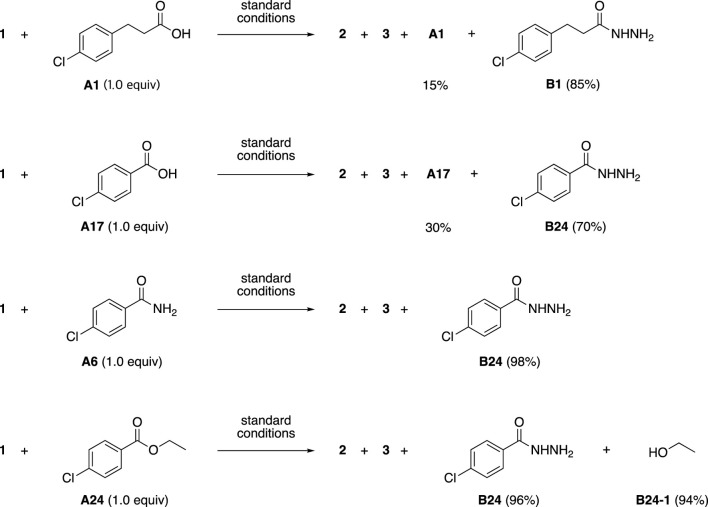
Formation of byproducts derived from additives **A1**, **A17**, **A6** and **A24**.

As mentioned above, although several carboxylic acids had positive additive effects, the carboxyl groups reacted with hydrazine to form the corresponding hydrazide (Scheme 4). Therefore, control experiments between carboxylic acids and hydrazides were performed to determine whether the positive effect was due to the carboxylic acid or the in situ-formed hydrazide.

We selected benzoic acid (**C14**) and 4-chlorobenzoic acid (**A17**) as representative carboxylic acids, and the corresponding hydrazides were synthesized to study their effects. As shown in Table 4, the addition of hydrazides had lower effects (Entries 4 and 7) than carboxylic acids (Entries 2 and 5). Furthermore, the addition of equal amounts of carboxylic acid and hydrazide gave intermediate-level results (Entries 3 and 6). These results clearly suggest that carboxylic acids accelerated the amide bond cleavage reaction, not hydrazides.

**TABLE 4:** Comparison of carboxylic acids and hydrazides.

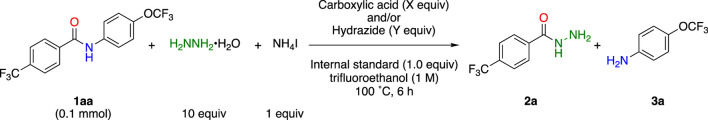			
Entry	Additive	X equiv/Y equiv	Yield of 2a (%)^a^
1	none	−	37
2	benzoic acid	1.0	59
3	benzoic acid/benzohydrazide	0.5/0.5	48
4	benzohydrazide	1.0	38
5	4-chlorobenzoic acid	1.0	59
6	4-chlorobenzoic acid/4-chlorobenzohydrazide	0.5/0.5	44
7	4-chlorobenzohydrazide	1.0	43

^a^ Determined by ^19^F NMR analysis of the crude mixture using 4-(trifluoromethoxy)anisole (0.1 mmol) as an internal standard.

#### 2.2.3 Lewis acid additives for accelerating the amide bond cleavage reaction

Because carboxylic acids are gradually converted to less effective hydrazides under the reaction conditions, we next examined Lewis acids as additives. Some Lewis acids are reported to accelerate C–H/C–C/C–O/C–N bond cleavage reactions and improve reaction efficiencies (Kita et al., 2013; Baglia et al., 2016; Gao et al., 2021).

In fact, the addition of 1.0 equiv of Zn(OTf)_2_ greatly accelerated the reaction, increasing the yield of **2a** from 37% to 91% under the conditions shown in Supplementary Table S9. Furthermore, when the amount of Lewis acid was reduced to 0.1 equiv, the additive effect was maintained, although the yield was reduced to 66% (Table 5, Entry 9). In this case, no products other than the substrate **1aa** and the target products **2a** and **3a** were observed.

**TABLE 5:** Screening of Lewis acid catalysts.

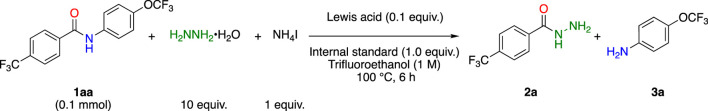					
Entry	Lewis acid	Yield of 2a (%)^*a*^	Entry	Lewis acid	Yield of 2a (%)^*a*^
1	none	37	11	AgOTf	27
2	CuBr	20	12	Cu(OTf)_2_	28
3	CuBr_2_	27	13	Ni(OTf)_2_	51
4	CuCl_2_	28	14	Co(OTf)_2_	48
5	PdCl_2_	34	15	Fe(OTf)_3_	63
6	Zn(OAc)_2_	52	16	Fe(OTf)_3_^*b*^	38
7	Cu(OAc)_2_	28	17	Sc(OTf)_3_	36
8	Pd(OAc)_2_	32	18	Y(OTf)_3_	42
9	Zn(OTf)_2_	66	19	Yb(OTf)_3_	41
10	Zn(OTf)_2_^*b*^	89	20	La(OTf)_3_	32
			21	Bi(OTf)_3_	34

^a^ Determined by ^19^F NMR analysis of the crude mixture using 4-(trifluoromethoxy)anisole (0.1 mmol) as an internal standard. ^b^Without addition of NH_4_I.

We then screened various Lewis acid catalysts for the amide bond cleavage reaction using General Procedure B (Table 5) (See Supplementary Material). Although many Lewis acids showed no positive effects, a catalytic amount of Fe(OTf)_3_ along with Zn(OTf)_2_ efficiently accelerated the amide bond cleavage reactions and increased the yield of product **2a** (Entry 15). Furthermore, in the case of Zn(OTf)_2_, the reaction was accelerated more efficiently in the absence of ammonium iodide (Entry 10). On the other hand, in the case of Fe, no acceleration effect was observed under conditions without NH_4_I (Entry 16).

#### 2.2.4 Scope of amide substrates with acidic additive/catalyst

Finally, with the optimized activation systems using carboxylic acids **C19** and **C27** (1.0 equiv) and Lewis acid catalysts Fe(OTf)_3_ and Zn(OTf)_2_ (0.1 equiv) in hand, the substrate generality of amide was examined using General Procedure B (Table 6) (See Supplementary Material).

**TABLE 6:** Substrate scope of amides with acidic additive/catalyst.

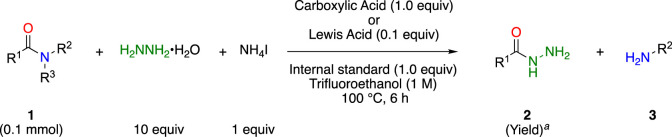						
Amide	none	C19	C27	Fe(OTf)_3_	Zn(OTf)_2_	Zn(OTf)_2_ without NH_4_I
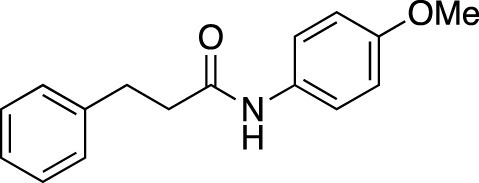 **1ba** ^b^	69%	73%	70%	63%	67%	45%
	83% (12 h)	91% (12 h)	88% (12 h)		84% (12 h)	
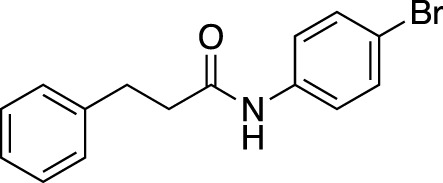 **1bb**	40%	71%	76%	40%	51%	76%
	67% (12 h)	>99% (12 h)	93% (12 h)			90% (12 h)
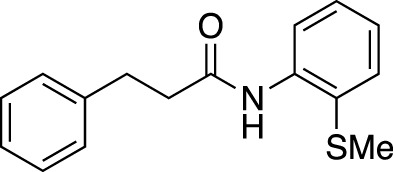 **1bc**	39%	53%	55%	44%	45%	77%
	67% (12 h)	76% (12 h)	76% (12 h)			87% (12 h)
		>99% (24 h)	>99% (24 h)			>99% (24 h)
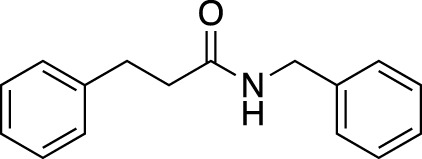 **1bd**	42%	51%	56%	62%	50%	38%
	67% (12 h)	>99% (24 h)	>99% (24 h)	79% (24 h)	82% (24 h)	
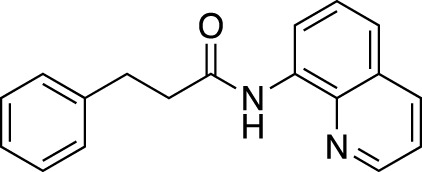 **1be** ^b^	30%	57%	54%	53%	76%	>99%
	54% (12 h)	80% (12 h)	82% (12 h)			
			>99% (24 h)			

^a^ Determined by ^1^H NMR analysis of the mixture using an internal standard.

^b^ At 90 °C.

We found that the additives also had a reaction-accelerating effect on other amide substrates. Again, to clarity the differences in the effects of these four systems, the results are shown for a reaction time of 6 h before the reaction is complete. Although the degree of effectiveness of each system varied depending on the substrates, in all cases the addition of 1.0 equiv of carboxylic acid was effective. In the case of 8-aminoquinoline amide **1be**, a highly effective and frequently used directing group amide, the system using a zinc catalyst without the addition of NH_4_I showed drastic acceleration effects, increasing the yield from 30% to 99% at 6 h. With acidic additives/catalysts, the reactions using amides **1bb**–**1bf** were almost completed by prolonging the reaction time. The addition of carboxylic or Lewis acids to the amide bond cleavage reaction is expected to be useful for cleavage of less reactive amide bonds.

## 3 Conclusion

In conclusion, we evaluated functional group compatibility in amide bond cleavage reactions using the FGE kit, which allows for accurate and rapid assessment of functional group compatibility using 26 FGE compounds with different functional groups. Except for some functional groups that react with hydrazine, we found many functional groups that are compatible. These evaluation experiments revealed that an unprecedented substitution reaction of additive **A13** with phenolic hydroxyl group proceeded. Moreover, carboxylic acids were discovered to accelerate the reaction, leading to the development of a new catalytic amide bond cleavage reaction with Zn(OTf)_2_. These results revealed the clear advantage of the FGE kit for both collecting data that can be applied for machine learning and discovering seeds for the development of new reactions.

## Data Availability

The raw data supporting the conclusion of this article will be made available by the authors, without undue reservation.
