# Supplementary material for: Evaluation of functional group compatibility and development of reaction-accelerating additives in ammonium salt-accelerated hydrazinolysis of amides
Source: Front Chem. 2024 May 22;12:1378746. doi: 10.3389/fchem.2024.1378746 (PMC11150581; doi:10.3389/fchem.2024.1378746)
Supplement: Supplementary file 1 [file DataSheet1.pdf]

Supplementary Material

1 General Experimental Details

All reactions were performed in flame-dried or oven-dried glassware under an argon atmosphere unless otherwise noted. Reagents and catalysts were obtained from commercial sources and used as received unless otherwise stated. Solvents were purchased from commercial sources and dried over molecular sieves before use. Flash silica gel column chromatography was performed with Kanto Chemical silica gel 60N (spherical neutral, particle size 40–50 μm). Automated flash column chromatography was performed using Biotage Selekt equipped with Biotage Sfär Silica HC Duo columns.

Nuclear magnetic resonance (NMR) spectra were acquired on 500 MHz Bruker Avance III spectrometer. Chloroform-*d* ($CDCl_3$) containing 0.03% tetramethylsilane (TMS) as the reference material or DMSO-d_6 without reference material was used. ^{1}H and $^{13}C\{^{1}H\}$ NMR chemical shifts are reported in ppm and referenced to tetramethylsilane or residual solvent peaks as internal standards (for $CDCl_3$, tetramethylsilane 0 ppm for ^{1}H and $CDCl_3$ 77.0 ppm for $^{13}C\{^{1}H\}$; for DMSO-d_6, 2.50 ppm for 1H and 39.5 ppm for $^{13}C\{^{1}H\}$). ^{19}F NMR chemical shifts are reported in ppm relative to trifluoromethylbenzene at −62.78 ppm as external reference. Coupling constants are reported in hertz. The following abbreviations are used: s = singlet, d = doublet, t = triplet, q = quartet, m = multiplet, brs = broad singlet, brd = broad doublet. High-resolution mass spectroscopy (HRMS) was obtained with Bruker MicrOTOF II (ESI) and Shimadzu LCMS-9030 with IonSense DART-OS (DART).

2 Preparation of Starting Materials

Reagents not specifically described were purchased from commercial suppliers and used as received without further purification. The FGE kit was prepared according to the literature (Saito et al., 2023).

Synthesis of *N*-[4-(Trifluoromethoxy)phenyl]-4-(trifluoromethyl)benzamide (1aa) (Cas no. 361364-86-5)

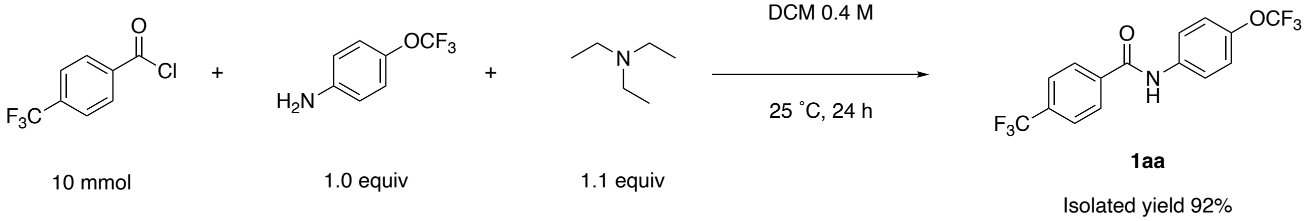

Scheme S1 Synthesis of **1aa**.

A 20 mL flask with a magnetic stir bar was added 4-(trifluoromethoxy)aniline (1.34 mL, 10.0 mmol, 1.0 equiv), triethylamine (1.48 mL, 11.0 mmol, 1.1 equiv) and dry dichloromethane (25 mL, 0.40 M) under an argon atmosphere. The mixture was cooled with ice bath before the addition of 4-(trifluoromethyl)benzoyl chloride (1.35 mL, 10.0 mmol, 1.0 equiv). The resulting mixture was stirred at room temperature for overnight. The crude mixture was quenched with saturated aqueous $NaHCO_3$ solution (20 mL). The crude mixture was extracted with CH_2Cl_2 (30 mL x 3) and the combined organic layers were dried over Na_2SO_4, filtered, and concentrated under reduced pressure. The crude product was purified by silica gel flash column chromatography using hexane/EtOAc = 5/1 as eluent to give **1aa** as a white solid (3.21 g, 92% yield).

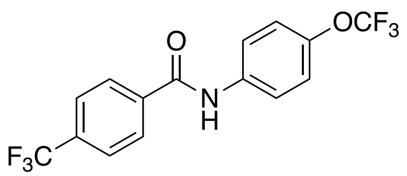

^{1}H NMR (500 MHz, $CDCl_3$) δ 7.97 (d, J = 7.5 Hz, 2H), 7.89 (brs, NH), 7.76 (d, J = 8.0 Hz, 2H), 7.67 (d, J = 9.0 Hz, 2H), 7.25 (d, J = 8.5 Hz, 2H). **^{13}C{^{1}H} NMR (125 MHz, $CDCl_3$)** δ 164.5, 145.9 (q, J_{C-F} = 1.6 Hz), 137.8, 133.8 (q, J_{C-F} = 32.6 Hz), 127.5, 126.8, 126.0 (q, J_{C-F} = 3.6 Hz), 123.5 (q, J_{C-F} = 270.9 Hz), 121.9, 121.6, 120.5 (q, J_{C-F} = 270.9 Hz). **^{19}F NMR (470 MHz, $CDCl_3$)** δ −57.0 (s, 3F), −61.4 (s, 3F). **HRMS (DART)** *m/z* calcd. for $C_{15}H_{10}F_6NO_2^+$ $[M + H]^+$ 350.0610, found 350.0617.

4-(Trifluoromethyl)benzohydrazide (2a) (Cas No. 339-59-3)

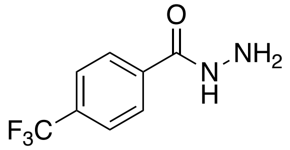

^{1}H NMR (500 MHz, $CDCl_3$) δ 7.86 (d, *J* = 8.0 Hz, 2H), 7.73 (d, *J* = 8.0 Hz, 2H), 7.48 (brs, NH), 4.14 (brs, NH_2). **$^{13}C\{^1H\}$ NMR (125 MHz, $CDCl_3$)** δ 167.4, 136.0, 133.7 (q, $J_{C\text{-}F}$ = 32.5 Hz), 127.4, 125.8 (q, $J_{C\text{-}F}$ = 3.5 Hz), 123.6 (q, $J_{C\text{-}F}$ = 270.9 Hz). **^{19}F NMR (470 MHz, $CDCl_3$)** δ −63.1 (s, 3F). **HRMS (DART)** *m/z* calcd. for $C_8H_8F_3N_2O^+$ $[M + H]^+$ 205.0583, found 205.0586.

4-Trifluoromethoxylanilne (3a) (Cas No. 461-82-5)

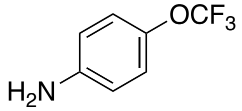

^{1}H NMR (500 MHz, $CDCl_3$) δ 6.97 (d, *J* = 8.5 Hz, 2H), 6.56 (d, *J* = 8.5 Hz, 2H), 3.65 (brs, NH_2). **$^{13}C\{^1H\}$ NMR (125 MHz, $CDCl_3$)** δ 145.2, 141.3 (q, $J_{C\text{-}F}$ = 1.4 Hz), 122.4, 120.7 (q, $J_{C\text{-}F}$ = 253.8 Hz), 115.5. **^{19}F NMR (470 MHz, $CDCl_3$)** δ −58.5 (s, 3F). **HRMS (DART)** *m/z* calcd. for $C_7H_7F_3NO^+$ $[M + H]^+$ 178.0474, found 178.0467.

***N*-(4-Methoxyphenyl)benzenepropanamide (1bb) (Cas No. 97754-31-9)**

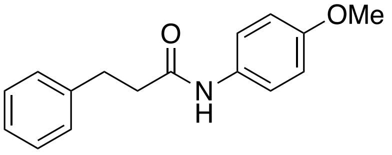

^{1}H NMR (500 MHz, $CDCl_3$) δ 7.32−7.29 (m, 4H), 7.26−7.20 (m, 3H), 6.94 (brs, NH), 6.83 (d, *J* = 8.5 Hz, 2H), 3.78 (s, 3H), 3.05 (t, *J* = 7.5 Hz, 2H), 2.63 (t, *J* = 7.5 Hz, 2H). **$^{13}C\{^1H\}$ NMR (125 MHz, $CDCl_3$)** δ 170.2, 156.5, 140.7, 130.8, 128.6, 128.4, 126.4, 121.9, 114.1, 55.5, 39.4, 31.7. **HRMS (DART)** *m/z* calcd. for $C_{18}H_{18}NO_2^+$ $[M + H]^+$ 256.1332, found 256.1336.

***N*-(4-Bromophenyl)benzenepropanamide (1bc) (Cas No. 316146-27-7)**

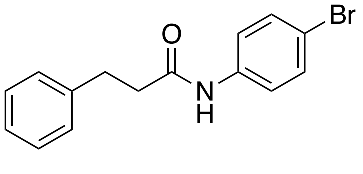

^{1}H NMR (500 MHz, $CDCl_3$) δ 7.40 (d, *J* = 9.0 Hz, 2H), 7.32−7.22 (m, 7H), 6.96 (brs, NH), 3.05 (t, *J* = 7.5 Hz, 2H), 2.65 (t, *J* = 7.5 Hz, 2H). **$^{13}C\{^1H\}$ NMR (125 MHz, $CDCl_3$)** δ 170.3, 140.3, 136.7, 131.9,

128.7, 128.4, 126.5, 121.4, 116.9, 39.5, 31.5. **HRMS (DART)** *m/z* calcd. for $C_{15}H_{15}BrNO^+$ $[M + H]^+$ 304.0332, found 304.0333.

_N_-[2-(Methylthio)phenyl]benzenepropanamide (1bd) (Cas No. 708222-26-8)

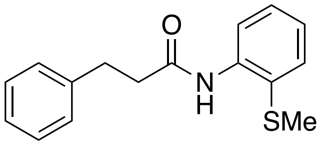

^{1}H NMR (500 MHz, DMSO-d_6) δ 9.32 (brs, NH), 7.35−7.29 (m, 6H), 7.22−7.14 (m, 3H), 2.92 (t, *J* = 8.0 Hz, 2H), 2.66 (t, *J* = 7.5 Hz, 2H), 2.39 (s, 3H). **^{13}C{^{1}H} NMR (125 MHz, $CDCl_3$)** δ 170.3, 140.5, 138.3,133.1, 129.0, 128.6, 128.4, 126.4, 125.0, 124.3, 120.6, 39.7, 31.5, 18.9. **HRMS (DART)** *m/z* calcd. for $C_{16}H_{18}NOS^+$ $[M + H]^+$ 272.1104, found 272.1103.

_N_-Benzyl-3-phenylpropionamide (1be) (Cas No. 10264-10-5)

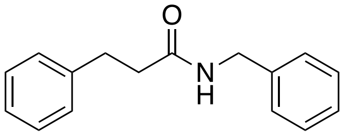

^{1}H NMR (500 MHz, $CDCl_3$) δ 7.31−7.26 (m, 5H), 7.22−7.19 (m, 3H), 7.15−7.14 (m, 2H), 5.63 (brs, NH), 4.40 (d, *J* = 5.5 Hz, 2H), 3.00 (t, *J* = 8.0 Hz, 2H), 2.52 (t, *J* = 8.0 Hz, 2H). **^{13}C{^{1}H} NMR (125 MHz, $CDCl_3$)** δ 171.8, 140.8, 138.1, 128.7, 128.6, 128.4, 127.8, 127.5, 126.3, 43.6, 38.5, 31.7. **HRMS (DART)** *m/z* calcd. for $C_{16}H_{18}NO^+$ $[M + H]^+$ 240.1383, found 240.1383.

_N_-8-Quinolinylbenzenepropanamide (1bf) (Cas No. 867347-49-7)

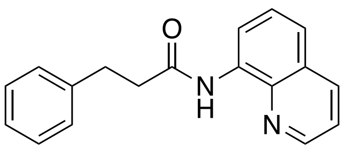

^{1}H NMR (500 MHz, $CDCl_3$) δ 9.79 (brs, NH), 8.78−8.77 (m, 2H), 8.16 (dd, *J* = 8.0 Hz, 1.5 Hz, 1H), 7.56−7.49 (m, 2H), 7.47 (dd, *J* = 8.0 Hz, 4.0 Hz, 1H), 7.30 (d, *J* = 5.0 Hz, 4H), 7.22−7.19 (m, 1H), 3.15 (t, *J* = 8.0 Hz, 2H), 2.89 (t, *J* = 8.0 Hz, 2H). **^{13}C{^{1}H} NMR (125 MHz, $CDCl_3$)** δ 170.7, 148.1, 140.8, 138.3, 136.4, 134.5, 128.6, 128.4, 127.9, 127.4, 126.2, 121.6, 121.4, 116.5, 39.7, 31.5. **HRMS (DART)** *m/z* calcd. for $C_{18}H_{17}N_2O^+$ $[M + H]^+$ 277.1335, found 277.1343.

3-Phenylpropanehydrazide (2b) (Cas No. 3538-68-9)

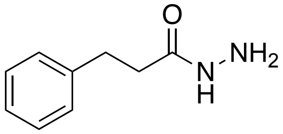

^{1}H NMR (500 MHz, CDCl$_3$) δ 7.31−7.18 (m, 5H), 6.57 (brs, NH), 3.86 (brs, NH_2), 2.98 (t, J = 7.5 Hz, 2H), 2.45 (t, J = 7.5 Hz, 2H). **^{13}C{^{1}H} NMR (125 MHz, CDCl$_3$)** δ 172.9, 140.5, 128.6, 128.3, 126.4, 36.3, 31.4. **HRMS (DART)** *m/z* calcd. for $C_9H_{13}N_2O^+$ $[M + H]^+$ 165.1022, found 165.1019.

4-Methoxyaniline (3b) (Cas No. 104-94-9)

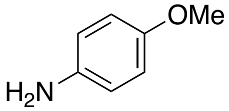

^{1}H NMR (500 MHz, CDCl$_3$) δ 6.74 (d, J = 9.0 Hz, 2H), 6.65 (d, J = 9.0 Hz, 2H), 3.74 (s, 3H), 3.36 (brs, NH_2). **^{13}C{^{1}H} NMR (125 MHz, CDCl$_3$)** δ 152.8, 140.0, 116.4, 114.8, 55.8. **HRMS (DART)** *m/z* calcd. for $C_7H_{10}NO^+$ $[M + H]^+$ 124.0757, found 124.0753.

4-Bromoaniline (3c) (Cas No. 106-40-1)

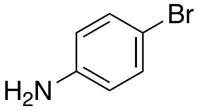

^{1}H NMR (500 MHz, CDCl$_3$) δ 7.23 (d, J = 9.0 Hz, 2H), 6.56 (d, J = 9.0 Hz, 2H), 3.65 (brs, NH_2). **^{13}C{^{1}H} NMR (125 MHz, CDCl$_3$)** δ 145.4, 132.0, 116.7, 110.2. **HRMS (DART)** *m/z* calcd. for $C_6H_7BrN^+$ $[M + H]^+$ 171.9756, found 171.9753.

2-(Methylthio)aniline (3d) (Cas No. 2987-53-3)

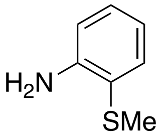

^{1}H NMR (500 MHz, CDCl$_3$) δ 7.34 (d, J = 6.5 Hz, 1H), 7.08 (ddd, J = 8.0 Hz, 8.0 Hz, 1.2 Hz, 1H), 6.72−6.69 (m, 2H), 4.25 (brs, NH_2), 2.34 (s, 3H). **^{13}C{^{1}H} NMR (125 MHz, CDCl$_3$)** δ 147.1, 133.5, 128.9, 120.2, 118.8, 114.9, 17.7. **HRMS (DART)** *m/z* calcd. for $C_7H_{10}NS^+$ $[M + H]^+$ 140.0528, found 140.0519.

Benzylamine (3e) (Cas No. 100-46-9)

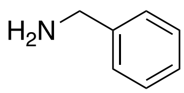

^{1}H NMR (500 MHz, CDCl$_3$) δ 7.34−7.22 (m, 5H), 3.86 (s, 2H), 1.50 (s, NH_2). **^{13}C{^{1}H} NMR (125 MHz, CDCl$_3$)** δ 143.4, 128.6, 127.1, 126.8, 46.5. **HRMS (DART)** *m/z* calcd. for $C_7H_{10}N^+$ $[M + H]^+$ 108.0808, found 108.0805.

8-Aminoquinoline (3f) (Cas No. 578-66-5)

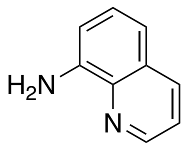

^{1}H NMR (500 MHz, CDCl$_3$) δ 8.76 (dd, J = 4.3 Hz, 1.5 Hz, 1H), 8.07 (dd, J = 8.5 Hz, 1.5 Hz, 1H), 7.38−7.32 (m, 2H), 7.15 (d, J = 8.0 Hz, 1H), 6.93 (dd, J = 7.5 Hz, 1.0 Hz, 1H), 5.01 (brs, NH_2). **^{13}C{^{1}H} NMR (125 MHz, CDCl$_3$)** δ 147.5, 144.0, 138.5, 136.0, 128.9, 127.4, 121.4, 116.0, 110.0. **HRMS (DART)** *m/z* calcd. for $C_9H_9N_2^+$ $[M + H]^+$ 145.0760, found 145.0760.

3 Optimization of the Amide Bond Cleavage Reaction Conditions

3.1 General Procedure A

To a 4 mL vial with a magnetic stir bar were added *N*-[4-(trifluoromethoxy)phenyl]-4-(trifluoromethyl)benzamide (**1aa**) (34.92 mg, 0.10 mmol, 1.0 equiv) and ammonium iodide (0–2.0 equiv), and trifluoroethanol (0.5–2.0 M) under an argon atmosphere. To the mixture were added hydrazine monohydrate (0–20 equiv), and mixture was stirred at 70–100 °C on a hot plate for 24 hours. The yields of products **2a** and **3a** were determined by ^{19}F NMR analysis of the crude mixture.

3.2 Result of Optimizations

The results were shown in the **Table 1** below.

Table 1 Optimization of reaction condition using amide **1aa**

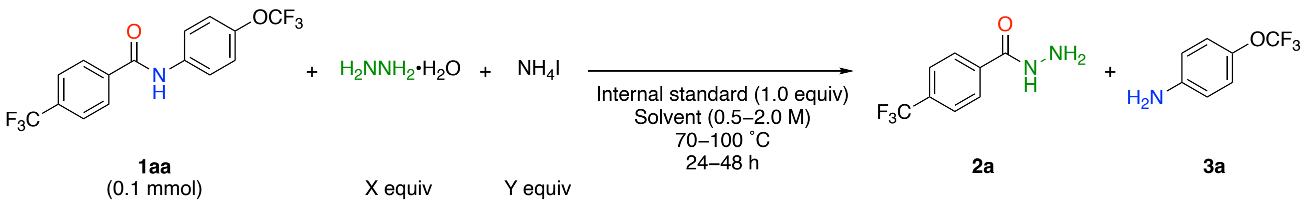

Entry	X equiv	Y equiv	Solvent	(M)	Temp. (°C)	Time (h)	Yield (%)[a] 2a	3a	1aa
1	10	1.0	ethanol	1.0	70	48	20	17	82
2	10	1.0	–	–	70	24	N.D.[b]	N.D.[b]	≥99
3	10	1.0	ethanol	1.0	100	24	85	81	17
4	10	1.0	hexafluoroisopropanol	1.0	100	24	82	82	18
5	10	1.0	trifluoroethanol	1.0	100	24	93	93	7
6	10	–	trifluoroethanol	1.0	100	24	52	53	48
7	10	2.0	trifluoroethanol	1.0	100	24	69	67	32
8	–	1.0	trifluoroethanol	1.0	100	24	N.D.[b]	N.D.[b]	≥99
9	20	1.0	trifluoroethanol	1.0	100	24	≥99	≥99	N.D.[b]
10	10	1.0	trifluoroethanol	0.5	100	24	66	67	33
11	10	1.0	trifluoroethanol	1.4	100	24	78	78	22
12	10	1.0	trifluoroethanol	2.0	100	24	76	76	24

[a] Determined by ^{19}F NMR analysis of the crude mixture using 4-(trifluoromethoxy)anisole (0.1 mmol) as an internal standard [b] Not detected.

4 Experimental Procedure for FGE Kit Evaluation

The evaluation of the additives for the amide bond cleavage reaction using the FGE kit was conducted in accordance with the literature procedure (Saito et al. 2023).

4.1 Detailed Procedure for Control Experiments with Additive A0

The experiment with 1.0 equiv of additive **A0** was carried out fire times (n = 5). The standard deviations were calculated using the STDEV.S function in Microsoft® Excel®.

The results were shown in the **Table S1** below.

Table S1 Results of control experiments with additive **A0**

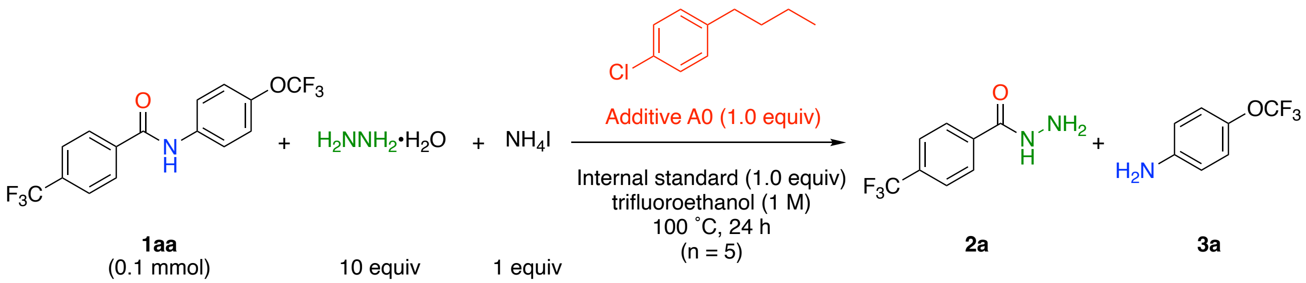

	1	2	3	4	5	Standard deviation (σ)
24h Yield of **2a** (%)[a]	72.0	73.6	73.1	74.7	73.5	**1.0**
24h Yield of **3a** (%)[a]	71.6	72.8	72.1	75.1	72.9	**1.3**
24h remaining additive **A0** (%)[b]	99.9	99.0	100.0	95.5	102.5	**2.5**

[a] Determined by ^{19}F NMR analysis of the crude mixture using 4-(trifluoromethoxy)anisole (0.1 mmol) as an internal standard [b] Determined by ^{1}H NMR analysis of the crude mixture using 4-(trifluoromethoxy)anisole (0.1 mmol) as an internal standard.

The standard deviation of yield (%) is 0.97, suggesting that it could be obtained sufficient reproducibility under conditions with additives. The reduction of the remaining additive (%) were not observed (97%), and it was obtained high reproducibility with a standard deviation of 2.52, indicating that the 4-chlorophenyl structure is tolerant to the reaction conditions.

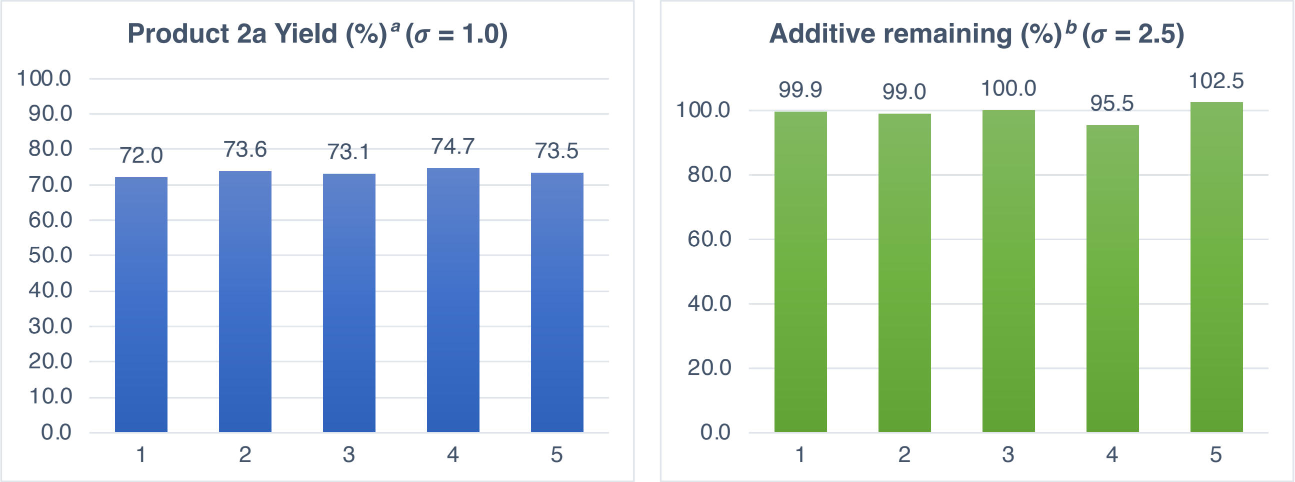

Figure 2 Reaction with additive **A0**

4.2 Detailed Procedure for Each Additives with Additives A1–A26

After fulfilling the reproducibility criteria of additive **A0**, a significant level of 1% F-test using the F.TEST function in Microsoft® Excel® was conducted to calculate the additive effects of the other additives, **A1**–**A26**, in the FGE kit.

4.3 Detailed Procedure for Assessing Yields Remaining Additives

To determine the significant differences in the data, the two-tailed Student's t-test or two-tailed Welch's t-test with a significance level of 1% were calculated. The two-tailed Student's t-test was used if the F-test showed that there was the same population variance between the date of additive **A0** and additive **A1–A26**, otherwise two-tailed Welch's t-test was used.

If the above t-test result showed a statistically significant increase in data from additives **A1–A26** the data were marked with a blue + mark. If the t-test result showed that there were no statistically significant differences, the data were marked with a green ± mark. If it decreased significantly larger than half of the mean value with the additive **A0**, the data were marked with a yellow − mark, and if it decreased significantly less than half of the additive **A0**, they were marked with a red x mark.

Since the remaining additives **A1–A26** did not increase, they were performed in the same method except using "one-tailed" t-tests with a significance level of 1%, and the data were indicated with the same mark.

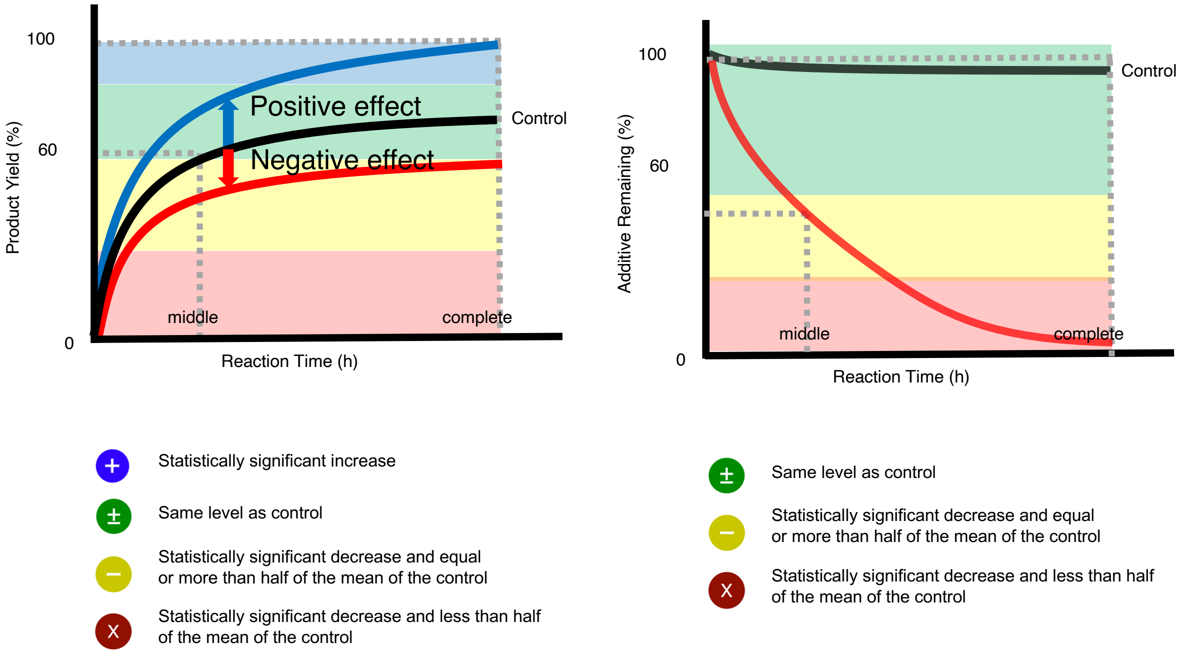

Figure 3 The symbols in FGE kit

5 Ammonium Salt-Accelerated Amide Bond Cleavage Reaction with FGE Kit Additives

5.1 General Procedure B

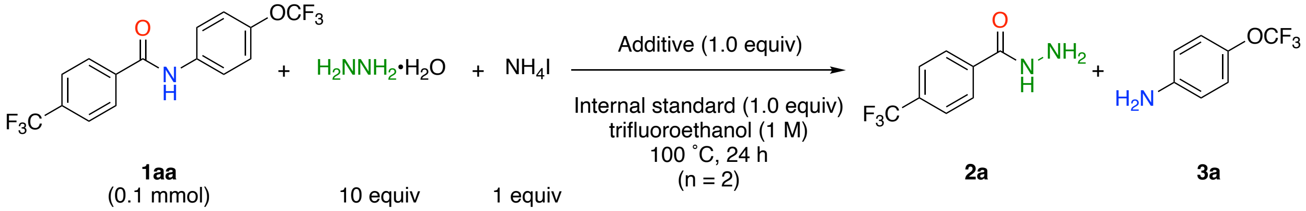

To a 4 mL vial with a magnetic stir bar was added *N*-[4-(trifluoromethoxy)phenyl]-4-(trifluoromethyl)benzamide (**1aa**) (34.92 mg, 0.10 mmol, 1.0 equiv) and ammonium iodide (14.49 mg, 0.10 mmol, 1.0 equiv), additive (if the additive was solid) (0.10 mmol, 1.0 equiv), and trifluoroethanol (0.10 mL, 1.0 M) under an argon atmosphere. To the mixture were added additive (if the additive was liquid) (0.10 mmol, 1.0 equiv), hydrazine monohydrate (48.5 μL, 1.0 mmol, 10 equiv), and mixture was stirred at 100 °C on a hot plate. After 6 hours, the vial was opened under an argon atmosphere and a sample was taken for NMR analysis. The vial was sealed again and stirred for an additional 18 hour (total 24 hours). The yields of products **2a** and **3a** were determined by ^{19}F NMR analysis, and the percentage of remaining additive was determined by ^{1}H NMR analysis of the crude mixture.

5.2 Results for Amide Bond Cleavage Reaction with Additives in FGE Kit

After reproducibility of additive **A0** was attained, we applied the other 26 additives in the FGE kit using General Procedure B. The tolerance of the functional group to the amide bond cleavage reaction were suggested by the experimental results.

The results were shown in the **Table S2** below.

Table S2 Experimental results for hydrazinolysis of amides using FGE kit

Additive	Structure	Yield of **2a** (%)[a] (± SE)[b]		Yield of **3a** (%)[a] (± SE)[b]		Additive remaining (%)[c]	
**A0**[d]	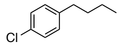	73 (± 0.4)		72 (± 0.3)		97	
**A1**	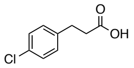	96 (± 0.2)	+	95 (±0.9)	+	14	X
**A2**[e]	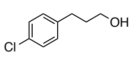	71 (± 1.7)	±	71 (±1.4)	±	99	±
**A3**	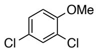	80 (± 0.5)	+	80 (±0.8)	±	99	±
**A4**	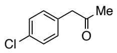	63 (± 0.3)	–	63 (±0.5)	–	0	X
**A5**	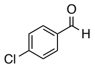	78 (± 2.5)	±	78 (±2.7)	±	0	X
**A6**[e]	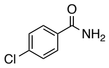	72 (± 1.7)	±	73 (±0.9)	±	0	X
**A7**[e]	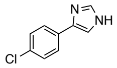	79 (± 2.5)	±	78 (±2.5)	±	95	±
**A8**	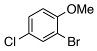	68 (± 1.6)	±	68 (±2.3)	±	>99	±
**A9**	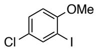	73 (± 2.7)	±	73 (±2.5)	±	95	±
**A10**	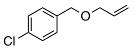	60 (± 1.8)	±	60 (±2.6)	±	44	X
**A11**	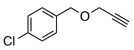	61 (± 1.4)	±	60 (±1.7)	±	27	X
**A12**	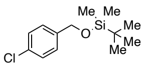	54 (± 3.3)	±	53 (±3.6)	±	84	±
**A13**	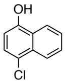	65 (± 0.4)	–	65 (±0.9)	±	64	±
**A14**	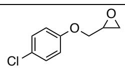	86 (± 0.6)	+	86 (±0.9)	+	95	±
**A15**	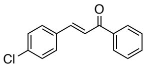	34 (± 8.9)	±	32 (±8.8)	±	0	X
**A16**	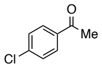	56 (± 0.3)	–	56 (±0.8)	–	0	X
**A17**	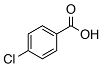	98 (± 0.3)	+	98 (±0.2)	+	28	X
**A18**	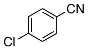	71 (± 5.0)	±	74 (±4.3)	±	9	X
**A19**	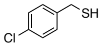	63 (± 1.2)	±	64 (±0.8)	±	>99	±
**A20**	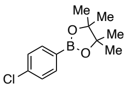	61 (± 0.1)	–	61 (±0.2)	–	81	±
**A21**	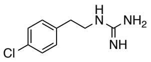	52 (± 2.8)	±	52 (±2.8)	±	0	X
**A22**	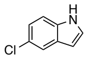	75 (± 1.0)	±	75 (±0.8)	±	96	±
**A23**	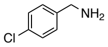	81 (± 3.8)	±	82 (±3.5)	±	98	±
**A24**	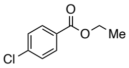	82 (± 1.1)	±	83 (±1.1)	±	0	X
**A25**	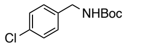	60 (± 0.7)	–	61 (±1.6)	±	94	±
**A26**	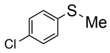	66 (± 6.6)	±	66 (±6.9)	±	92	±

[a] Determined by ^{19}F NMR analysis of the crude mixture using 4-(trifluoromethoxy)anisole (0.1 mmol) as an internal standard.
[b] Standard error. [c] Determined by ^{1}H NMR analysis of the mixture using 4-(trifluoromethoxy)anisole (0.1 mmol) as an internal standard. [d] n = 5. [e] n = 4.

5.3 Byproducts of Amide Bond Cleavage Reaction

The byproducts formed from the additives were isolated from the crude mixture or synthesized by the method reported in the literature when isolation was difficult, and their structures were confirmed using NMR and mass spectrometry analysis.

5.3.1 Byproducts B1 and B24 from A1, A6, A17 and A24

Additives **A1** and **A17** with carboxyl groups, **A6** with amide group, and **A24** with ester group reacted with hydrazine to afford the corresponding acyl hydrazides such as **B1** and **B24**. **B1** and **B24** were synthesized by the reported methods[2] and confirmed using NMR and mass spectrometry analysis.

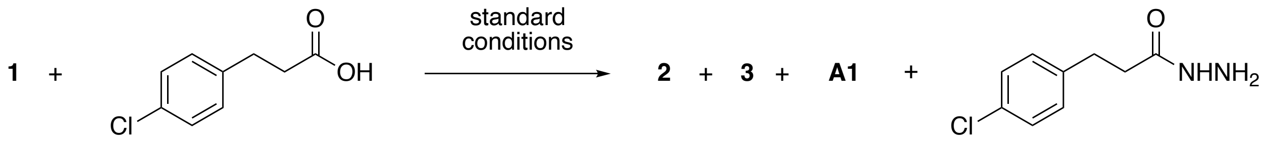

A1 (1.0 equiv) 15% **B1** (85%)

Scheme S2 Formation of byproduct **B1** from additive **A1**

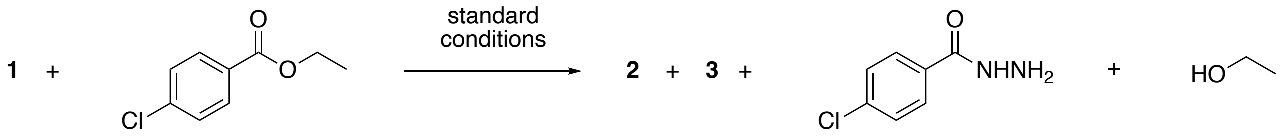

A24 (1.0 equiv) **B24** (96%) **B24-1** (94%)

Scheme S3 Formation of byproduct **B24** from additive **A24**

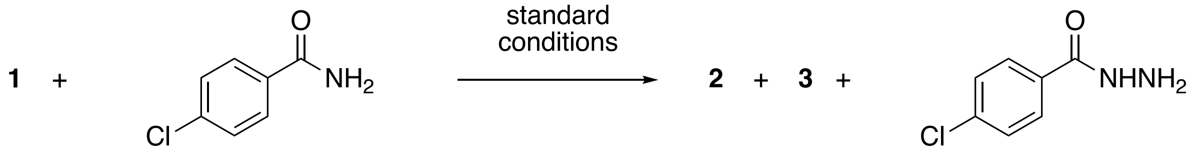

A6 (1.0 equiv) **B24** (98%)

Scheme S4 Formation of byproduct **B24** from additive **A6**

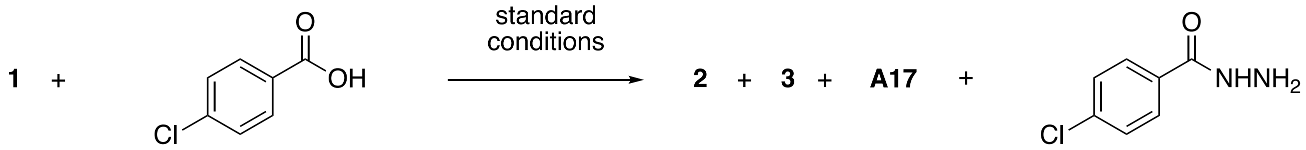

A17 (1.0 equiv) 30% **B24** (70%)

Scheme S5 Formation of byproduct **B24** from additive **A17**

5.3.1.1 Synthesis of Byproduct B1 Derived from Additives A1 (Dulla et al. 2012; La Pietra et al. 2018)

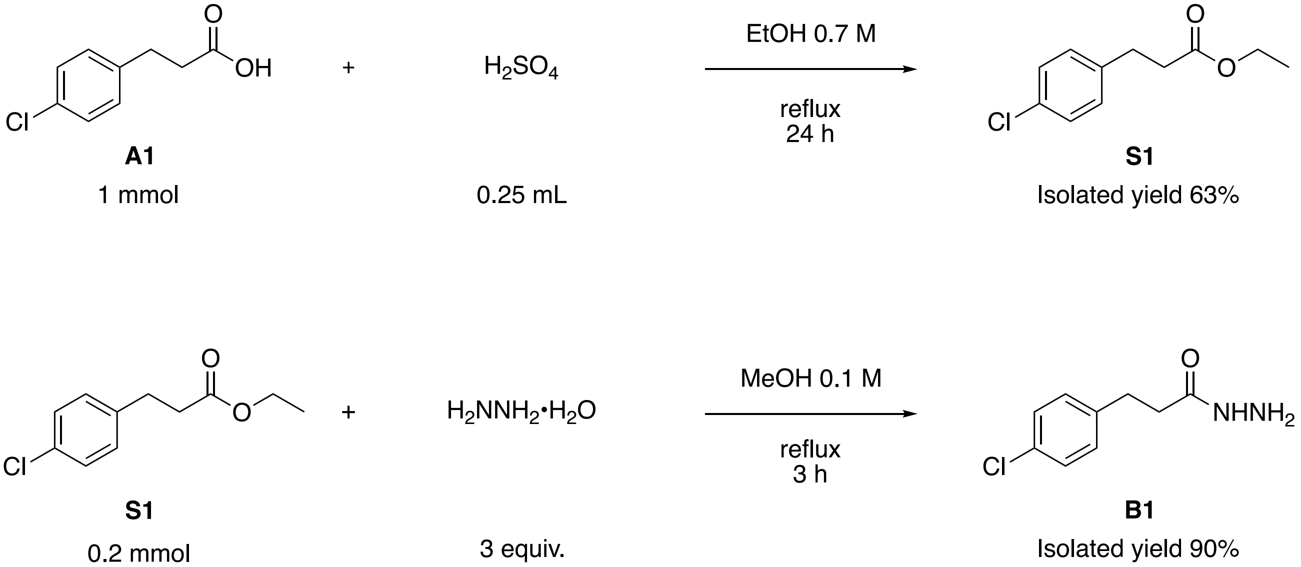

Scheme S6 Synthesis of byproduct **B1** from additive **A1**

(a) A 4 mL vial with a magnetic stir bar was added 3-(4-chlorophenyl)propionic acid (**A1**) (184.62 mg, 1.0 mmol, 1.0 equiv), ethanol (1.5 mL, 0.67 M), and sulfuric acid (0.25 mL, 4.7 equiv) under an argon atmosphere. The mixture was heated to reflux for 24 hours. The reaction mixture was cooled to room temperature and was quenched with saturated aqueous Na_2CO_3 solution (20 mL). The crude mixture was extracted with ethyl acetate (30 mL x 3) and the combined organic layers were dried over Na_2SO_4, filtered, and concentrated under reduced pressure. The crude product was purified by silica gel flash column chromatography using hexane/EtOAc = 1/1 as eluent to give **S1** as an oil (133.00 mg, 63% yield).

(b) A 4 mL vial with a magnetic stir bar was added ethyl 3-(4-chlorophenyl)propanoate (**S1**) (42.53 mg, 0.20 mmol, 1.0 equiv), methanol (2.0 mL, 0.10 M), and hydrazine monohydrate (29.1 μL, 0.60 mmol, 3.0 equiv) under an argon atmosphere. The mixture was heated to reflux for 3 hours. Methanol was removed under high vacuum and a solid obtained was purified by recrystallization from EtOH. The white solid was collected by filtration and dried under vacuum to give **B1** (35.54 mg, 90% yield).

3-(4-Chlorophenyl)propanehydrazide (B1) (Cas No. 1132-73-6)

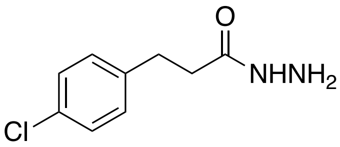

^{1}H NMR (500 MHz, $CDCl_3$) δ 7.25 (d, *J* = 8.0 Hz, 2H), 7.12 (d, *J* = 8.0 Hz, 2H), 6.60 (brs, NH), 3.88 (br s, NH_2), 2.95 (t, *J* = 7.5 Hz, 2H), 2.43 (t, *J* = 7.5 Hz, 2H). **$^{13}C\{^1H\}$ NMR (125 MHz, $CDCl_3$)** δ 172.5, 138.9, 132.2, 129.7, 128.7, 36.1, 30.7. **HRMS (DART)** *m/z* calcd. for $C_9H_{12}ClN_2O^+$ $[M + H]^+$ 199.0633, found 199.0634.

5.3.1.2 Synthesis of Byproduct B24 Derived from Additives A24 (Dulla et al., 2012; La Pietra et al., 2018)

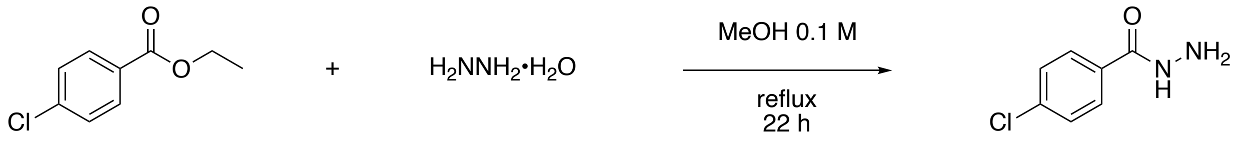

Scheme S7 Synthesis of byproduct **B24** from additive **A24**

A 4 mL vial with a magnetic stir bar was added ethyl 4-chlorobenzoate (**A24**) (31.1 μL, 0.20 mmol, 1.0 equiv), methanol (2.0 mL, 0.10 M), and hydrazine monohydrate (29.1 μL, 0.60 mmol, 3.0 equiv) under an argon atmosphere. The mixture was heated to reflux for 22 hours. The crude mixture was evaporated and purified by silica gel flash column chromatography using hexane/EtOAc = 1/10 as eluent to give a white solid **B24** (20.40 mg, 60% yield).

4-Chlorobenzohydrazide (B24) (Cas No. 536-40-3)

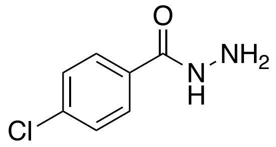

^{1}H NMR (500 MHz, $CDCl_3$) δ 7.68 (d, *J* = 8.0 Hz, 2H), 7.43 (d, *J* = 8.0 Hz, 2H), 7.30 (brs, NH), 4.14 (brs, NH_2). **$^{13}C\{^1H\}$ NMR (125 MHz, $CDCl_3$)** δ 167.7, 138.3, 131.0, 129.1, 128.3. **HRMS (DART)** *m/z* calcd. for $C_7H_8ClN_2O^+$ $[M + H]^+$ 171.0320, found 171.0320.

5.3.2 Byproducts B4, B5 and B16 from A4, A5, and A16

Additives **A4**, **A5** and **A16** with carbonyl groups reacted with hydrazine to afford the corresponding acyl hydrazones such as **B4**, **B5** and **B16**. **B4**, **B5** and **B16** were synthesized by the reported methods[3] and confirmed using NMR and mass spectrometry analysis.

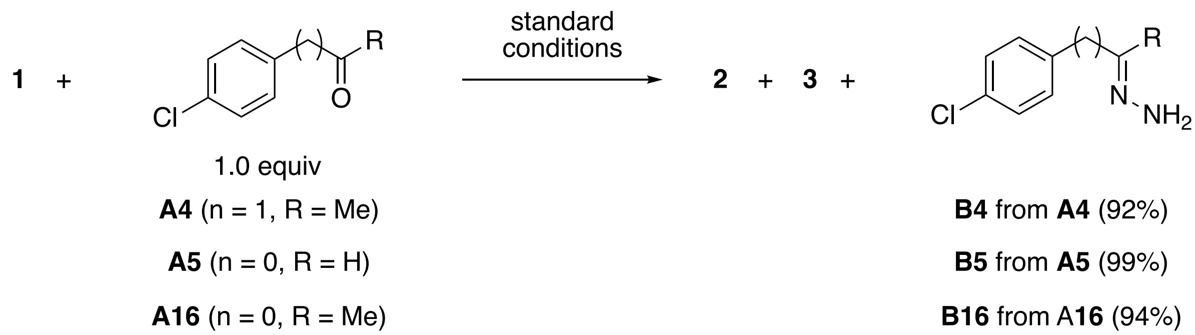

Scheme S8 Formation of byproduct **B4**, **B5**, and **B16** from additive **A4**, **A5**, and **A16**

5.3.2.1 Synthesis of Byproduct B4, B5, B16 Derived from Additives A4, A5, A16 (Poh et al. 2015; Poh et al. 2017)

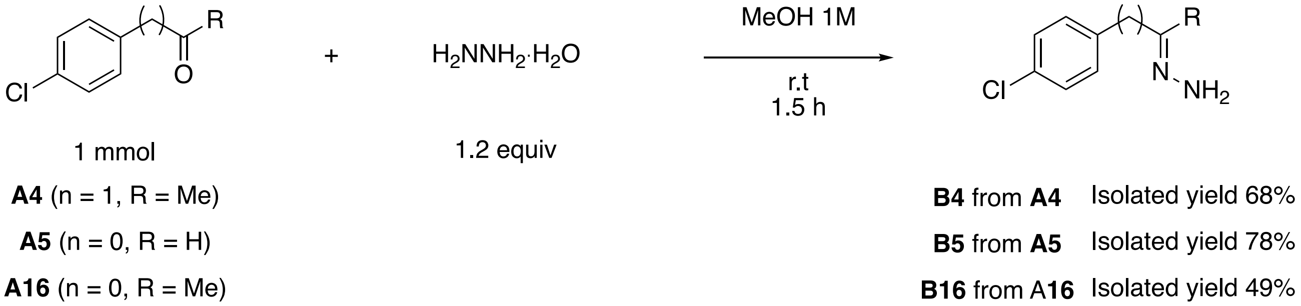

Scheme S9 Synthesis of byproduct **B4**, **B5**, and **B16** from additive **A4**, **A5**, and **A16**

A 20 mL vial with a magnetic stir bar was added aldehyde/ketone (**A4, A5, A16**) (1.0 mmol, 1.0 equiv), methanol (10 mL, 0.10 M), and hydrazine monohydrate (58.2 µL, 1.2 mmol, 1.2 equiv) under an argon atmosphere. The mixture stirred at 80 °C for 1.5 hours. The solvent was evaporated for removal. The crude mixture was extracted with diluted with water (20 mL) and ethyl acetate (30 mL x 3) and the combined organic layers were dried over Na_2SO_4, filtered, and concentrated under reduced pressure to give the corresponding hydrazones (**B2, B3, B16**).

1-(4-Chlorophenyl)-2-propanone hydrazone (B4) (Cas No. 1865893-61-3)

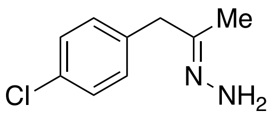

^{1}H NMR (500 MHz, CDCl$_3$) δ (only the peaks derived from an isomer are shown) 7.26 (d, J = 8.0 Hz, 2H), 7.14 (d, J = 8.0 Hz, 2H), 4.97 (brs, NH_2), 3.47 (s, 2H), 1.67 (s, 3H). **^{13}C{^{1}H} NMR (125 MHz, CDCl$_3$)** δ (only the peaks derived from an isomer are shown) 149.5, 136.4, 132.2, 130.3, 128.7, 44.7, 13.2. **HRMS (DART)** *m/z* calcd. for $C_9H_{12}ClN_2^+$ $[M + H]^+$ 183.0684, found 183.0683.

(4-Chlorobenzylidene)hydrazine (B5) (Cas No. 52372-80-2)

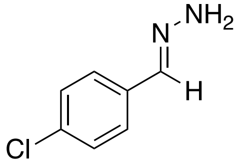

^{1}H NMR (500 MHz, CDCl$_3$) δ (only the peaks derived from an isomer are shown) 7.68 (s, 1H), 7.47 (d, J = 8.5 Hz, 2H), 7.31 (d, J = 8.5 Hz, 2H), 5.53 (brs, NH_2). **^{13}C{^{1}H} NMR (125 MHz, CDCl$_3$)** δ (only the peaks derived from an isomer are shown) 141.5, 134.2, 133.7, 128.8, 127.3. **HRMS (DART)** *m/z* calcd. for $C_7H_8ClN_2^+$ $[M + H]^+$ 155.0371, found 155.0367.

[1-(4-Chlorophenyl)ethylidene]hydrazine (B16) (Cas No. 40137-41-5)

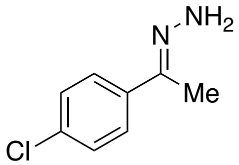

^{1}H NMR (500 MHz, CDCl$_3$) δ (only the peaks derived from an isomer are shown) 7.58 (d, J = 8.5 Hz, 2H), 7.31 (d, J = 8.5 Hz, 2H), 5.38 (brs, NH_2), 2.10 (s, 3H). **^{13}C{^{1}H} NMR (125 MHz, CDCl$_3$)** δ (only the peaks derived from an isomer are shown) 145.9, 137.8, 133.9, 128.4, 126.7, 11.5. **HRMS (DART)** *m/z* calcd. for $C_8H_{10}ClN_2^+$ $[M + H]^+$ 169.0527, found 169.0527.

5.3.3 Byproducts B15 from A15 and B18 from A18

Additive **A15** with an enone moiety and additive **A18** with a nitrile moiety reacted with hydrazine to afford the nitrogen-containing heterocycles pyrazoline **B15** and tetrazine **B18**. **B15** and **B18** were isolated from the crude mixture and confirmed using NMR and mass spectrometry analysis.

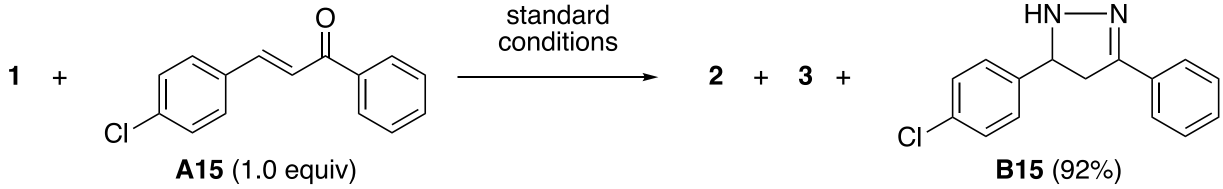

Scheme S10 Formation of byproduct **B15** from additive **A15**

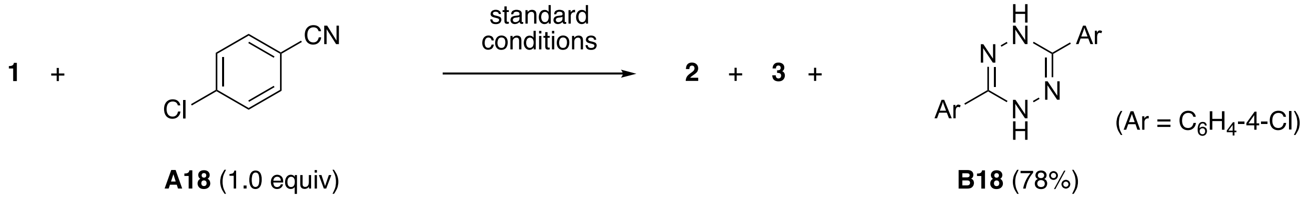

Scheme S11 Formation of byproduct **B18** from additive **A18**

5-(4-Chlorophenyl)-4,5-dihydro-3-phenyl-1*H*-pyrazole (B15) (Cas No. 89144-73-0)

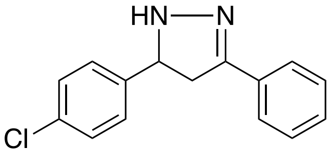

^{1}H NMR (500 MHz, CDCl$_3$) δ 7.70−7.66 (m, 2H), 7.40−7.32 (m, 8H, Ar-H, NH), 4.92 (dd, *J* = 11.0 Hz, 9.5 Hz, 1H), 3.49 (dd, *J* = 16.0 Hz, 11.0 Hz, 1H), 3.00 (dd, *J* = 16.5 Hz, 9.0 Hz, 1H). **^{13}C{^{1}H} NMR (125 MHz, CDCl$_3$)** δ 151.4, 141.2, 133.5, 132.6, 129.0, 129.0, 128.6, 127.8, 126.0, 63.7, 41.5. **HRMS (DART)** *m/z* calcd. for $C_{15}H_{14}ClN_2^+$ [M + H]$^+$ 257.0840, found 257.0841.

3,6-Bis(4-chlorophenyl)-1,4-dihydro-1,2,4,5-tetrazine (B18) (Cas No. 53876-70-3)

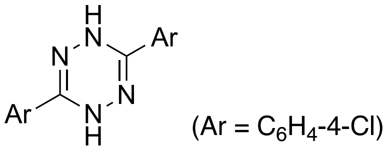

^{1}H NMR (500 MHz, DMSO-*d$_6$*) δ 8.08 (d, *J* = 8.5 Hz, 4H), 7.65 (d, *J* = 8.5 Hz, 4H), 6.34 (s, 2H). **^{13}C{^{1}H} NMR (125 MHz, DMSO-*d$_6$*)** δ 154.0, 135.0, 130.4, 129.1, 126.4. **HRMS (DART)** *m/z* calcd. for $C_{14}H_{11}Cl_2N_4^+$ [M + H]$^+$ 305.0355, found 305.0350.

5.3.4 Byproducts B13 from A13

Additive **A13** with the phenolic hydroxyl group and Ar-Cl moiety were reacted to amino groups to give **B13**. **B13** was isolated from the crude mixture and confirmed using NMR and mass spectrometry analysis.

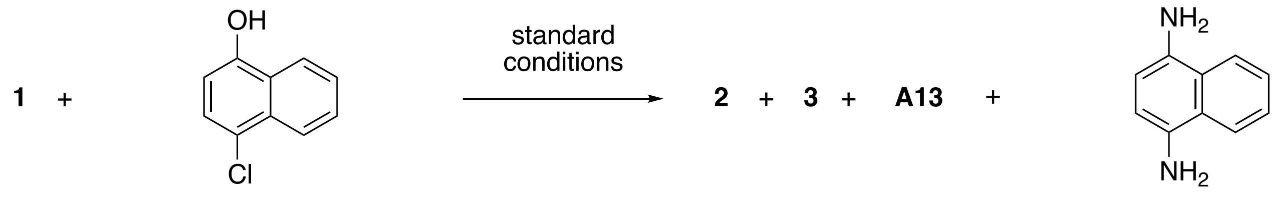

A13 (1.0 equiv) (64%) **B13** (22%)

Scheme S12 Formation of byproduct **B13** from additive **A13**

1,4-Naphthalenediamine (B13) (Cas no. 2243-61-0)

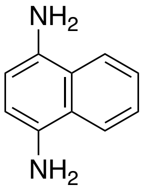

^{1}H NMR (500 MHz, Acetone-*d*$_6$) δ 7.85−7.83 (m, 2H), 7.26−7.24 (m, 2H), 6.51 (s, 2H), 4.30 (brs, 4H). **^{13}C{^{1}H} NMR (125 MHz, Acetone-*d*$_6$)** δ 135.4, 124.7, 123.9, 122.2, 110.1. **HRMS (DART)** *m/z* calcd. for $C_{10}H_{11}N_2^+$ $[M + H]^+$ 159.0917, found 159.0890.

5.3.5 Byproducts B21 from A21

Additive **A21** with guanidine reacted into amine **B21**. **B21** was isolated from the crude mixture and confirmed using NMR and mass spectrometry analysis.

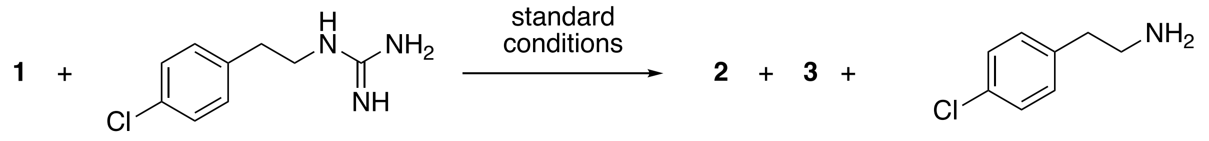

A21 (1.0 equiv) **B21** (98%)

Scheme S13 Formation of byproduct **B21** from additive **A21**

2-(4-Chlorophenyl)ethylamine (B21) (Cas No. 156-41-2)

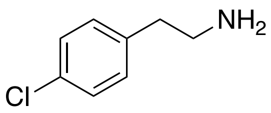

^{1}H NMR (500 MHz, $CDCl_3$) δ 7.26 (d, *J* = 8.5 Hz, 2H), 7.13 (d, *J* = 8.5 Hz, 2H), 2.94 (t, *J* = 7.0 Hz, 2H), 2.71 (t, *J* = 7.0 Hz, 2H), 1.15 (brs, NH_2). **^{13}C{^{1}H} NMR (125 MHz, $CDCl_3$)** δ 138.3, 131.9, 130.2, 128.5, 43.5, 39.4. **HRMS (DART)** *m/z* calcd. for $C_8H_{11}ClN_2^+$ $[M + H]^+$ 156.0575, found 156.0572.

5.4 Two-Dimensional Representations of the Product Yield (%) and Remaining Additives (%)

The two-dimensional plot shows the degree of the functional group compatibility for each reaction briefly. The positions of the number and structure of the additives were shown on the graph.

Table S3 The number and structure of the additives in FGE kit

Additive No.	Additive structure	Additive No.	Additive structure	Additive No.	Additive structu
Additive 1	RCO_2H	Additive 10	$RCH{=}CH_2$	Additive 19	RSH
Additive 2	ROH	Additive 11	RC≡CH	Additive 20	ArBpin
Additive 3	ArCl	Additive 12	ROTBS	Additive 21	Guanidine
Additive 4	RC(=O)R	Additive 13	ArOH	Additive 22	Indole
Additive 5	ArCHO	Additive 14	epoxide	Additive 23	RNH_2
Additive 6	$ArCONH_2$	Additive 15	RCH=CHC(=O)R	Additive 24	$ArCO_2R$
Additive 7	Imidazole	Additive 16	ArC(=O)R	Additive 25	$RNHCO_2{}^t$Bu
Additive 8	ArBr	Additive 17	$ArCO_2H$	Additive 26	ArSMe
Additive 9	ArI	Additive 18	ArCN		

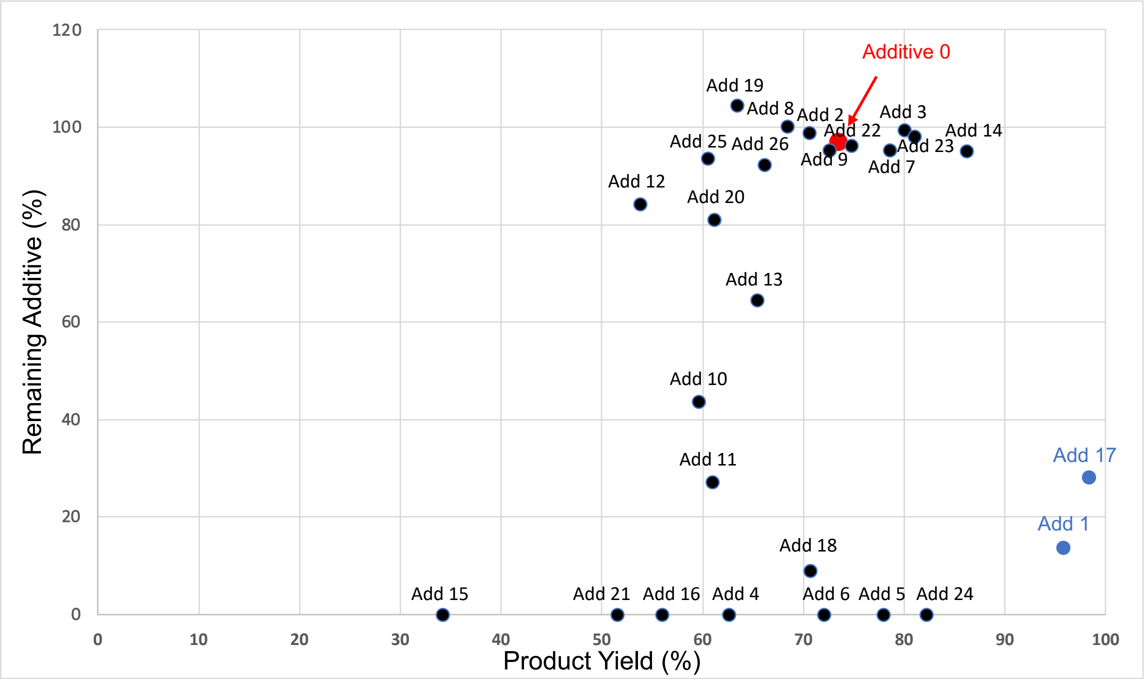

Figure S1 Two-dimensional with the number of the additives

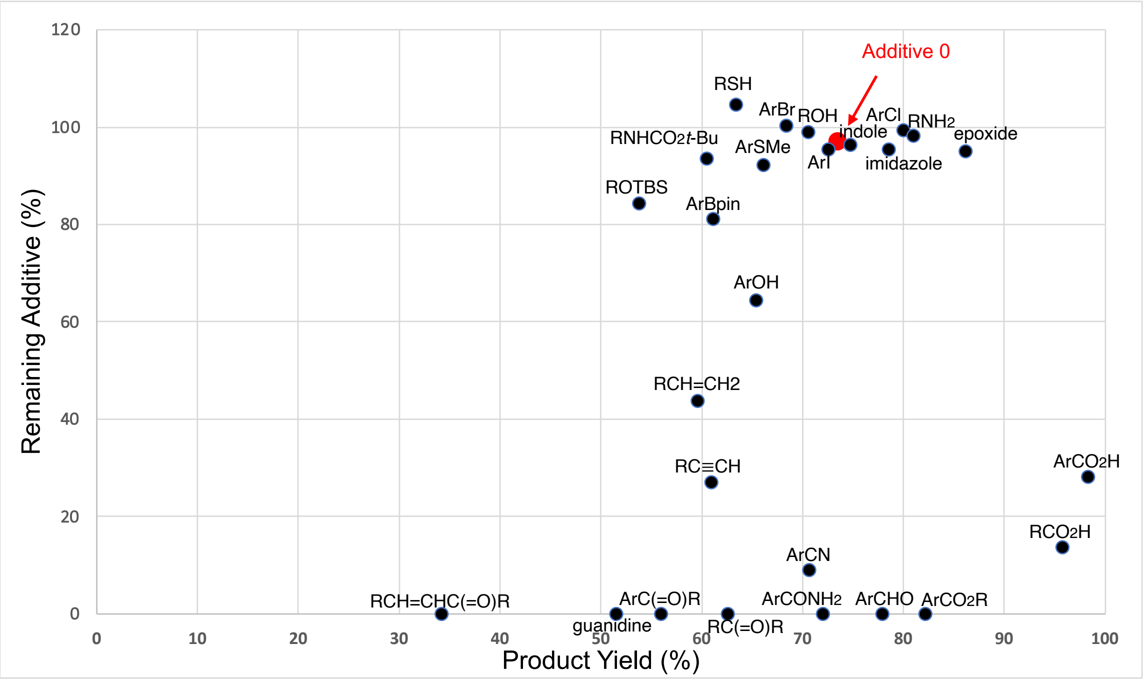

Figure S2 Two-dimensional with the structure of the additives

6 Acidic Additives for Accelerating the Amide Bond Cleavage Reaction

6.1 Screening of Brønsted Acid Additives

Reaction was performed according to the general procedure B using various inorganic and organic Brønsted acids as additives. The results were shown in the **Table 3** below. To evaluate the effects of additives on the reaction rate, the yield of products was examined for 6 hours during the reaction process. Products **2a** and **3a** were obtained in the same yields, so only the yield of product **2a** was shown in the table.

Table 3. Screening of Brønsted acid additives

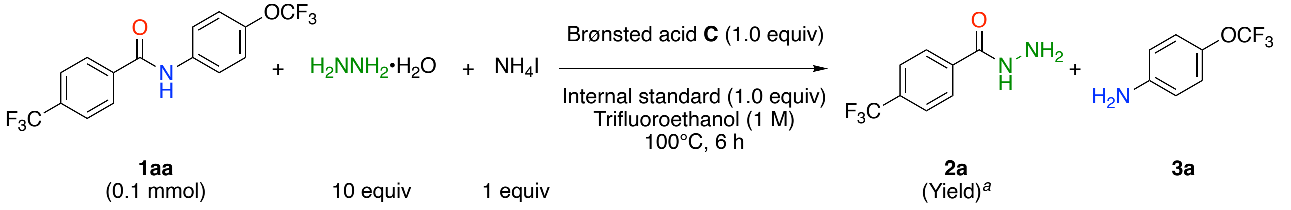

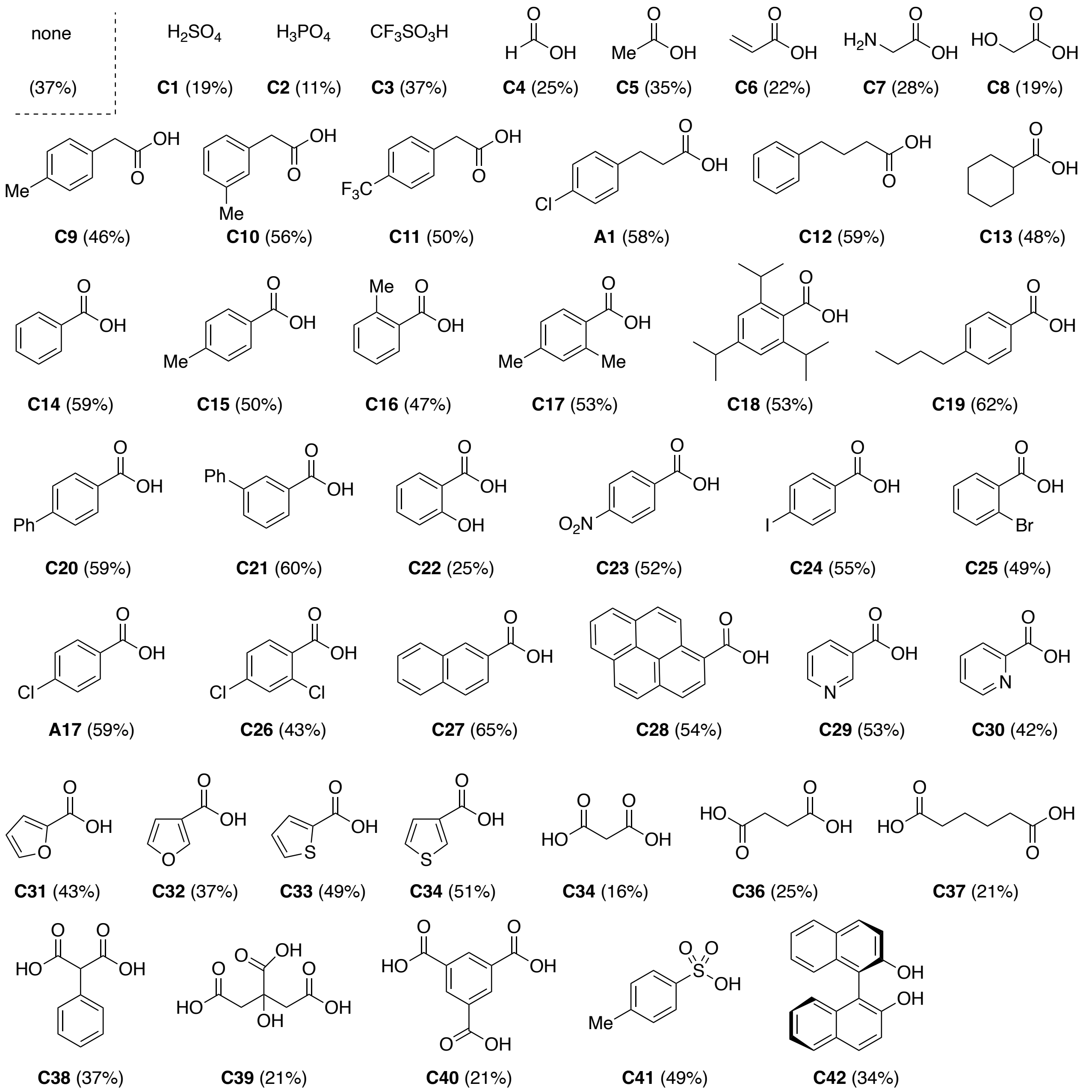

[a] Determined by ^{19}F NMR analysis of the crude mixture using 4-(trifluoromethoxy)anisole (0.1 mmol) as an internal standard

6.1.1 Control Experiments Between Carboxylic acid and Hydrazide

Reaction was performed according to the general procedure B using benzoic acid (**C14**) and 4-chlorobenzoic acid (**A17**) which are carboxylic acids, and corresponding hydrazides were selected as additives. Then, control experiments were performed.

The results were shown in the **Table S4** below.

As a result of the control experiments, it was confirmed that additives with carboxylic acid moiety accelerated the reaction. A graphical representation was generated to facilitate intuitive validation (**Figure S3**).

Table S4 Comparison of carboxylic acids and hydrazides

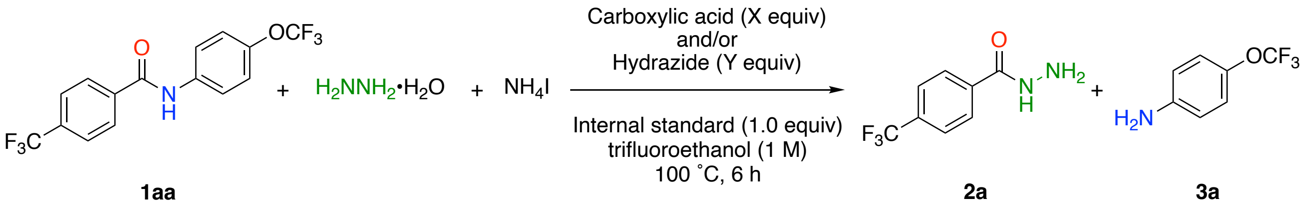

Entry	Additive	Equiv	Yield (%)[a]	
			**2a**	**3a**
1	None	–	37	37
2	Benzoic acid	1.0	59	60
3	Benzoic acid/Benzohydrazide	0.5/0.5	48	48
4	Benzohydrazide	1.0	38	39
5	4-Chlorobenzoic acid	1.0	59	59
6	4-Chlorobenzoic acid/4-Chlorobenzohydrazide	0.5/0.5	44	44
7	4-Chlorobenzohydrazide	1.0	43	42

[a] Determined by ^{19}F NMR analysis of the crude mixture using 4-(trifluoromethoxy)anisole (0.1 mmol) as an internal standard.

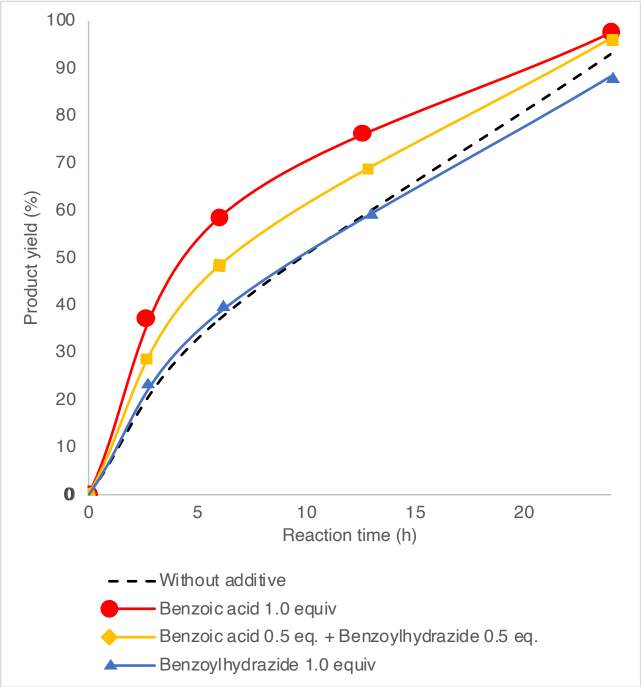

Figure S3 Graphs of reaction with carboxylic acid and/or hydrazide

6.1.2 Effect of Carboxylic Acid

Reaction was performed according to the general procedure B using 1.0 equivalent of 4-fluorobenzoic acid as an additive. The results were shown in the **Table S5** below.

The product yield and the remaining additive over time were compared. In the absence of an additive, the amide bond cleavage reaction proceeded gradually over time, resulting in a smooth increase in the yield of product **2a**. When 4-fluorobenzoic acid was added as an additive, it was observed that the product yield increased steadily without a rapid alteration in the reaction rate. It suggested that the acid additive only facilitates the amide bond cleavage reaction without exerting additional effects. A graphical representation was generated to facilitate intuitive validation (**Figure S4**).

Table S5 Variations over time in product yield and amount of 4-fluorobenzoic acid

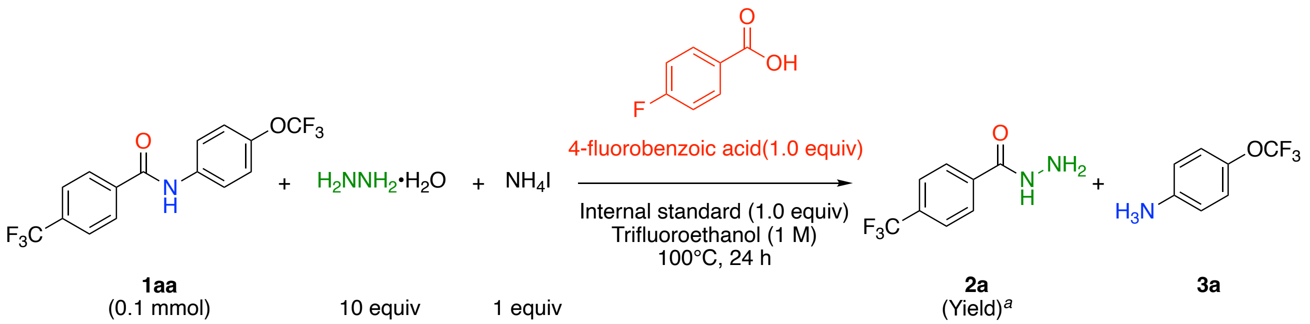

Entry	Additive		Reaction time (h) 0	4	8	12	16	20	24
1	None	Yield of **2a** (%) [a]	0	24	48	65	78	87	93
2	4-fluoro benzoic acid	Yield of **2a** (%) [a]	0	37	56	72	92	96	99
		remaining of additive (%) [b]	100	85	67	56	42	32	24

[a] Determined by ^{19}F NMR analysis of the crude mixture using 4-(trifluoromethoxy)anisole (0.1 mmol) as an internal standard. [b] Determined by ^{1}H NMR analysis of the crude mixture using 4-(trifluoromethoxy)anisole (0.1 mmol) as an internal standard.

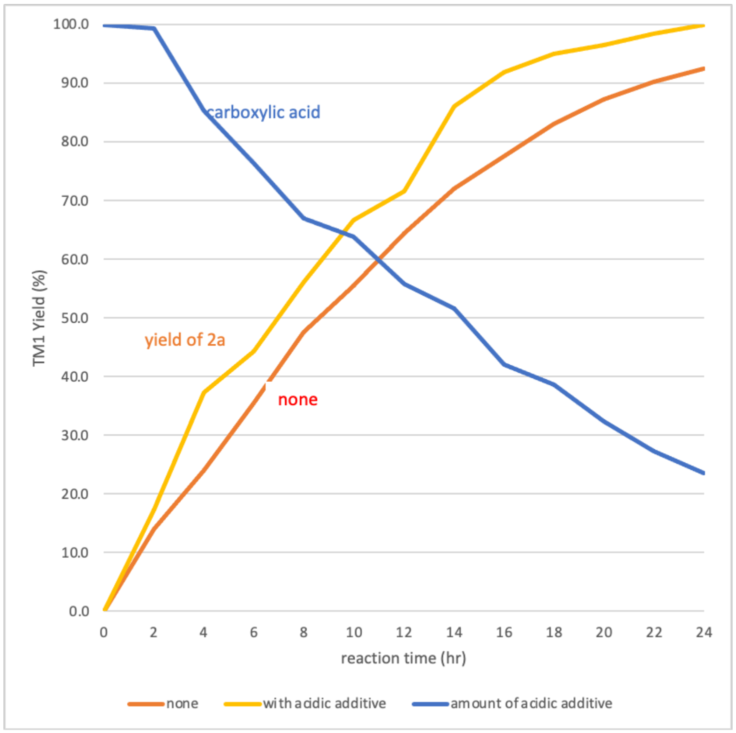

Figure S4 Graphs of the reaction with 4-fluorobenzoic acid

6.1.3 Amount of Carboxylic Acid

Reaction was performed according to the general procedure B using 0–2 equivalent of benzoic acid (**C14**) as an additive. The results were shown in the **Table S6** below.

It can be seen that the reaction accelerated as the amount of additives increased. However, an excess of additives could affect the reaction, so 1.0 equivalent was added. A graphical representation was generated to facilitate intuitive validation (**Figure S5**).

Table S6 Amount of benzoic acid (**C14**)

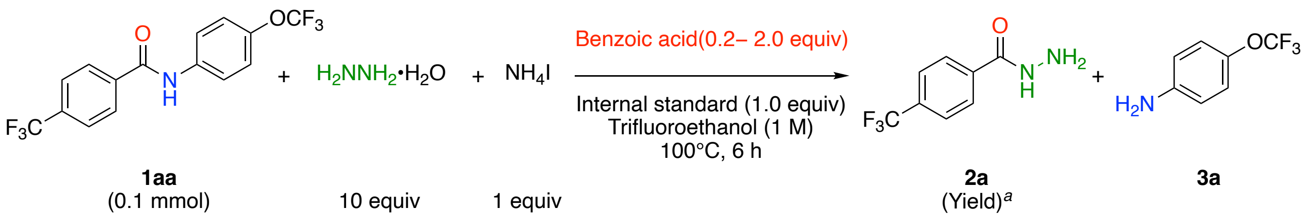

Entry	Additive	Equiv	Yield (%)[a]	
			**2a**	**3a**
1	None	–	37	37
2	Benzoic acid	2.0	64	64
3	Benzoic acid	1.2	56	57
4	Benzoic acid	1.0	55	52
5	Benzoic acid	0.8	49	49
6	Benzoic acid	0.6	42	42
7	Benzoic acid	0.4	36	36
8	Benzoic acid	0.2	32	30

[a] Determined by ^{19}F NMR analysis of the crude mixture using 4-(trifluoromethoxy)anisole (0.1 mmol) as an internal standard.

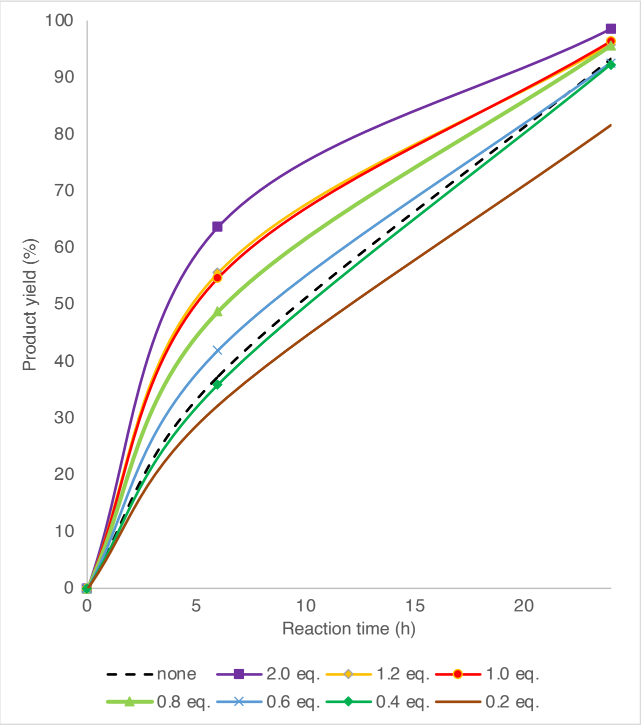

Figure S5 Graphs of reaction with benzoic acid

6.1.4 Amount of *ortho and/or para-Substituted* Carboxylic Acid

Reaction was performed according to the general procedure B using 1.0 equivalent of *ortho*-and/or *para*-substituted carboxylic acid as an additive. The results were shown in the **Table S7** below.

ortho-Substituted carboxylic acid did not react with hydrazine due to steric hindrance, however, did not significantly affect product yields.

Table S7 Amount *ortho*- and/or *para*-substituted carboxylic acid

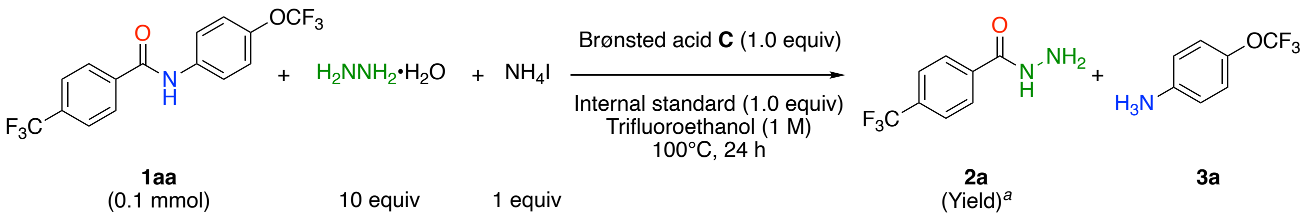

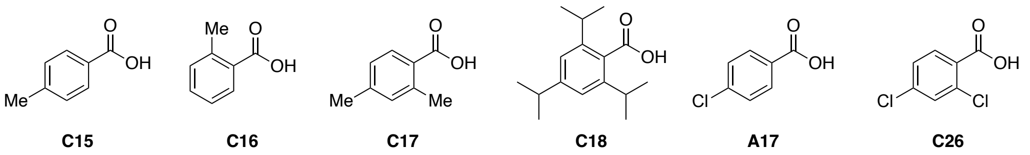

Entry	Additive	Product yield (%)[a]		Remaining additive (%)[b]	Hydrazine from additive (%)[b]
		**2a**	**3a**		
1	**C15**	94	94	66	34
2	**C16**	95	95	69	0
3	**C17**	97	97	87	0
4	**C18**	96	95	104	0
5	**A17**	99	99	28	72
6	**C26**	91	90	86	0

[a] Determined by ^{19}F NMR analysis of the crude mixture using 4-(trifluoromethoxy)anisole (0.1 mmol) as an internal standard. [b] Determined by ^{1}H NMR analysis of the crude mixture using 4-(trifluoromethoxy)anisole (0.1 mmol) as an internal standard.

6.2 Screening of Lewis Acid Catalysts

Reactions were performed according to the general procedure B using Lewis acid as additives. The results were shown in the **Table S8** below. To evaluate the effects of additives on the reaction rate, the yield of products was examined for 6 hours during the reaction process.

Table S7 Screening of Lewis acid catalysts

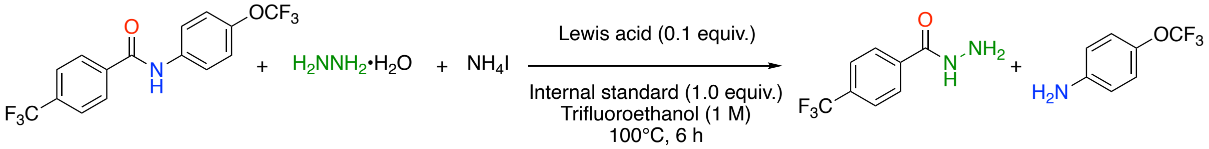

Entry	Lewis acid	Yield (%)[a] 2a	3a	Entry	Lewis acid	Yield (%)[a] 2a	3a
1	none	37	37	11	AgOTf	27	26
2	CuBr	20	22	12	$Cu(OTf)_2$	36	36
3	$CuBr_2$	27	25	13	$Ni(OTf)_2$	51	50
4	$CuCl_2$	28	29	14	$Co(OTf)_2$	48	44
5	$PdCl_2$	34	32	15	$Fe(OTf)_3$	65	65
6	$Zn(OAc)_2$	52	51	16	$Fe(OTf)_3$[b]	38	34
7	$Cu(OAc)_2$	28	26	17	$Sc(OTf)_3$	36	33
8	$Pd(OAc)_2$	32	26	18	$Y(OTf)_3$	42	43
9	$Zn(OTf)_2$	66	65	19	$Yb(OTf)_3$	41	40
10	$Zn(OTf)_2$[b]	89	89	20	$La(OTf)_3$	32	32
				21	$Bi(OTf)_3$	34	35

[a] Determined by ^{19}F NMR analysis of the crude mixture using 4-(trifluoromethoxy)anisole (0.1 mmol) as an internal standard. [b] Without addition of NH_4I.

6.2.1 Amount of Lewis Acid

Reaction was performed according to the general procedure B using 0–1 equivalent of zinc(II) trifluoromethanesulfonate as an additive. The results were shown in the **Table S9** below. To evaluate the effects of additives on the reaction rate, the yield of products was examined for 6 hours during the reaction process.

In the case of metal catalysts, the reaction accelerated as the amount of additives increased. However, even with a 0.1 equivalent, there was a sufficient effect. A graphical representation was generated to facilitate intuitive validation (**Figure S6**).

Table S8 Amount of $Zn(OTf)_2$

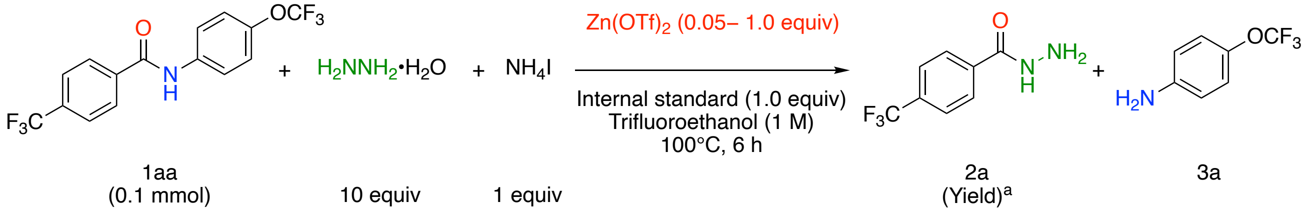

Entry	Additive	Equiv	Yield (%)[a] 2a	3a
1	None	–	37	37
2	$Zn(OTf)_2$	0.05	44	43
3	$Zn(OTf)_2$	0.10	66	65
4	$Zn(OTf)_2$	1.0	91	93

[a] Determined by ^{19}F NMR analysis of the crude mixture using 4-(trifluoromethoxy)anisole (0.1 mmol) as an internal standard.

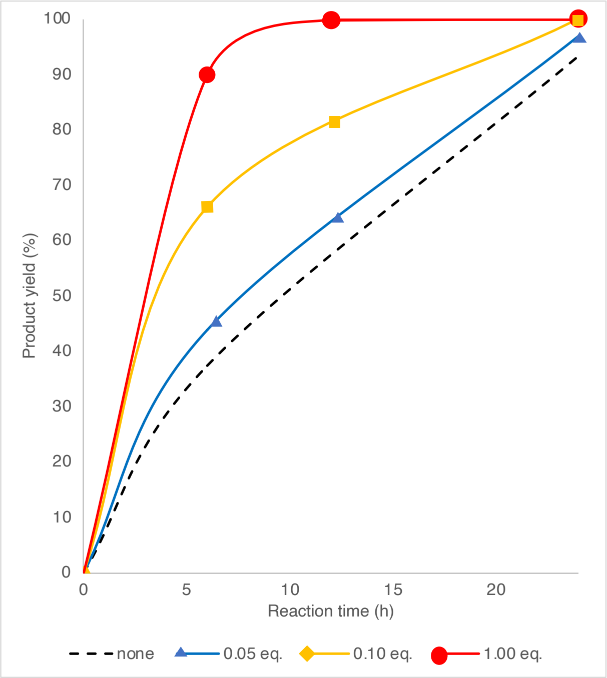

Figure S6 Graphs of reaction with $Zn(OTf)_2$

6.2.2 The Presence of NH_4I for $Zn(OTf)_2$

Reaction was performed according to the general procedure B using 0.1 equivalent of zinc(II) or iron(III) trifluoromethanesulfonate as an additive. The results were shown in the **Table S10** below. To evaluate the effects of additives on the reaction rate, the yield of products was examined for 6 hours during the reaction process.

Furthermore, in the case of $Zn(OTf)_2$, when ammonium iodide was not present, the reaction was accelerated more efficiently. On the other hand, in the case of $Fe(OTf)_3$, no acceleration effect was observed under conditions without NH_4I. A graphical representation was generated to facilitate intuitive validation (**Figure S7**).

Table S9 Results of amide bond cleavage reaction in the presence of NH_4I for Lewis acids

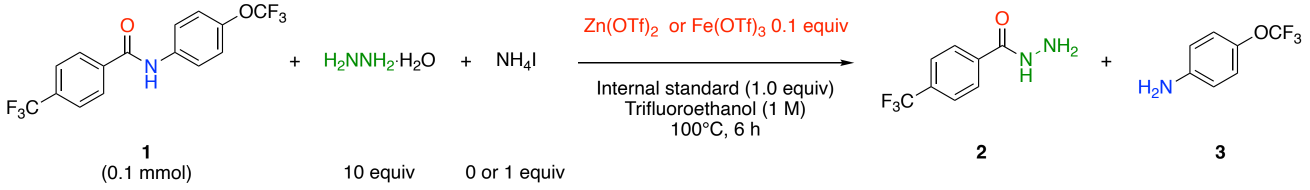

Entry	Lewis acid	Ammonium salt	Equiv	Yield (%)[a]	
				**2a**	**3a**
1	–	None	1.0	37	37
2	$Zn(OTf)_2$	Without NH_4I	–	89	89
3	$Zn(OTf)_2$	With NH_4I	1.0	66	65
4	$Fe(OTf)_3$	Without NH_4I	–	63	63
5	$Fe(OTf)_3$	With NH_4I	1.0	38	34

[a] Determined by ^{19}F NMR analysis of the crude mixture using 4-(trifluoromethoxy)anisole (0.1 mmol) as an internal standard.

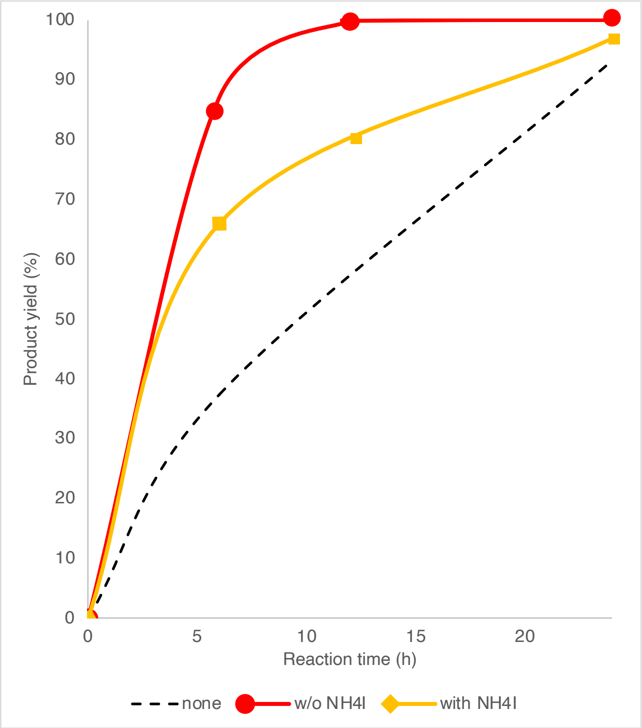

Figure S7 Graphs of reaction in the presence of NH_4I for $Zn(OTf)_2$

6.3 Ammonium Salt-Accelerated Amide Bond Cleavage Reaction with Acid Additives

In short, the screening of various acid additives indicated that 0.1 equivalent of $Zn(OTf)_2$ and $Fe(OTf)_3$ and 1.0 equivalent of 4-butylbenzoic acid (**C19**) and 2-naphthoic acid (**C27**) had great effect in accelerating the amide bond cleavage reaction. A graphical representation was generated to facilitate intuitive validation (**Figure S8**).

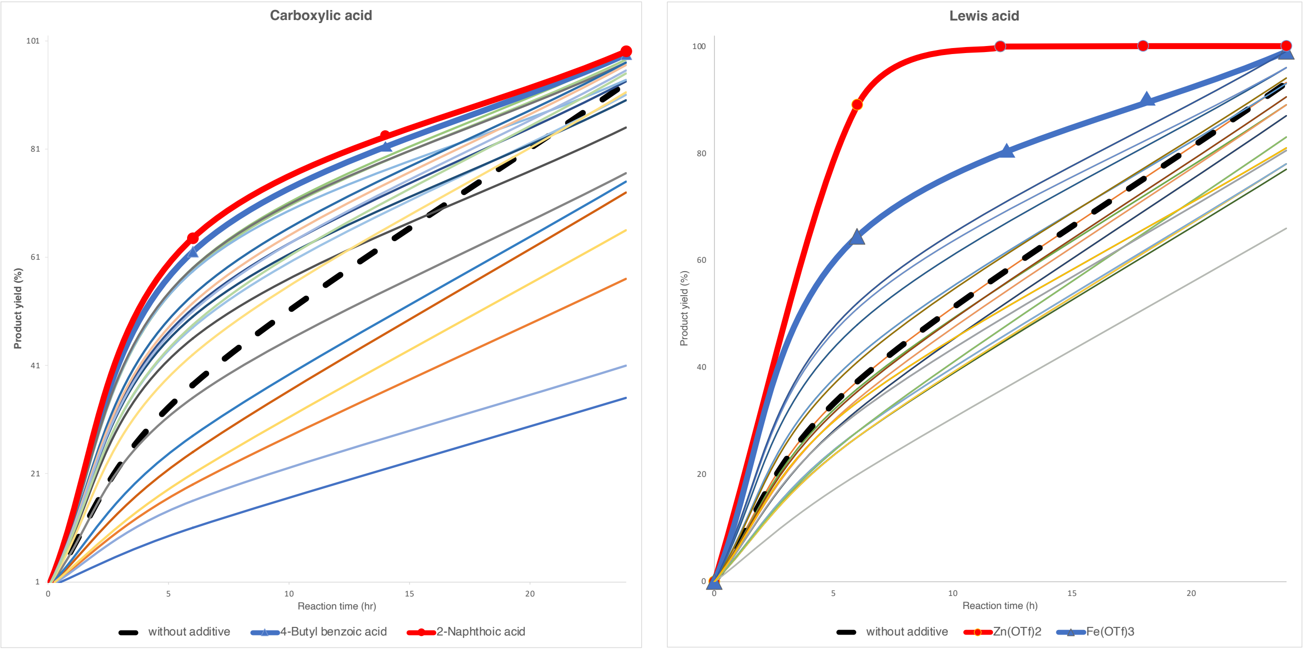

Figure S8 Graphs of the results for acid additives

6.4 Application to Amide Cleavage Reaction with Acid Additives for Other Substrates

Reaction was performed according to the general procedure B using $Zn(OTf)_2$, $Fe(OTf)_3$, 4-butylbenzoic acid (**C19**), and 2-naphthoic acid (**C27**) as additives, and amide compound **1bb**−**1bf** as substrates. The results were shown in the **Table 6** below. To evaluate the effects of additives on the reaction rate, the yield of products was examined for 6 hours during the reaction process. Products **2** and **3** were obtained in the same yields, so only the yield of product **2** was shown in the table.

Table 6. Substrate Scope of amides with acidic additive/catalyst

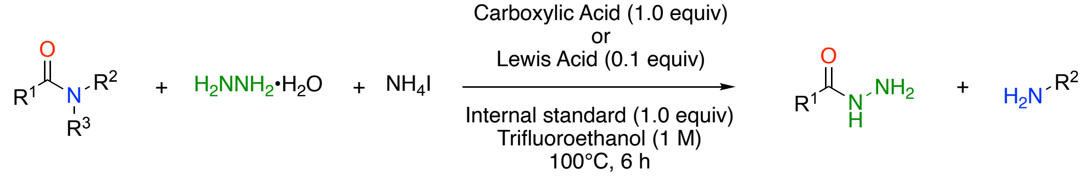

1 (0.1 mmol)	10 equiv	1 equiv	2 (Yield)[a]	3

Amide	none	**C19**	**C27**	$Fe(OTf)_3$	$Zn(OTf)_2$	$Zn(OTf)_2$ without NH_4I
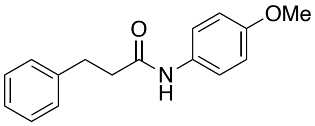 **1bb**[b]	69%<br>83% (12 h)	73%<br>91% (12 h)	70%<br>88% (12 h)	63%	67%<br>84% (12 h)	45%
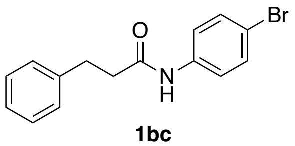 **1bc**	40%<br>67% (12 h)	71%<br>>99% (12 h)	76%<br>93% (12 h)	40%	51%	76%<br>90% (12 h)
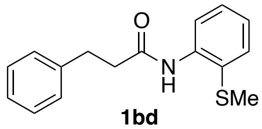 **1bd**	39%<br>67% (12 h)	53%<br>76% (12 h)<br>>99% (24 h)	55%<br>76% (12 h)<br>>99% (24 h)	44%	45%	77%<br>87% (12 h)<br>>99% (24 h)
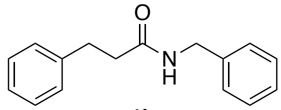 **1be**	42%<br>67% (12 h)	51%<br>>99% (24 h)	56%<br>>99% (24 h)	62%<br>79% (24 h)	50%<br>82% (24 h)	38%
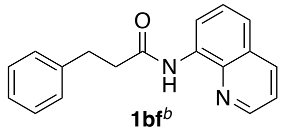 **1bf**[b]	30%<br>54% (12h)	57%<br>80% (12 h)	54%<br>82% (12 h)<br>>99% (24 h)	53%	76%	?99%

[a] Determined by ^1H NMR analysis of the crude mixture using an internal standard. [b] At 90 ˚C. [c] Without NH_4I.

7 References

Dulla, B., Wan, B., Franzblau, S. G., Kapavarapu, R., Reiser, O., Iqbal, J., and Pal, M. (2012). Construction and functionalization of fused pyridine ring leading to novel compounds as potential antitubercular agents. *Bioorganic & medicinal chemistry letters*, 22(14), 4629-4635. doi:10.1016/j.bmcl.2012.05.096

La Pietra, V., Sartini, S., Botta, L., Antonelli, A., Ferrari, S. M., Fallahi, P., *et al.* (2018). Challenging clinically unresponsive medullary thyroid cancer: Discovery and pharmacological activity of novel RET inhibitors. *European Journal of Medicinal Chemistry*, 150, 491-505. doi:10.1016/j.ejmech.2018.02.080

Poh, J. S., Tran, D. N., Battilocchio, C., Hawkins, J. M., and Ley, S. V. (2015). A Versatile Room-Temperature Route to Di-and Trisubstituted Allenes Using Flow-Generated Diazo Compounds. *Angewandte Chemie*, 127(27), 8031-8034. doi:10.1002/ange.201501538

Poh, J. S., Makai, S., von Keutz, T., Tran, D. N., Battilocchio, C., Pasau, P., and Ley, S. V. (2017). Rapid Asymmetric Synthesis of Disubstituted Allenes by Coupling of Flow-Generated Diazo Compounds and Propargylated Amines. *Angewandte Chemie International Edition*, 56(7), 1864-1868. doi:10.1002/anie.201611067

Saito, N., Nawachi, A., Kondo, Y., Choi, J., Morimoto, H., and Ohshima, T. (2023). Functional group evaluation kit for digitalization of information on the functional group compatibility and chemoselectivity of organic reactions. *Bulletin of the Chemical Society of Japan*, 96(5), 465-474. doi:10.1246/bcsj.20230047

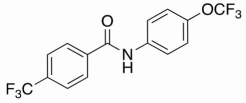

1aa

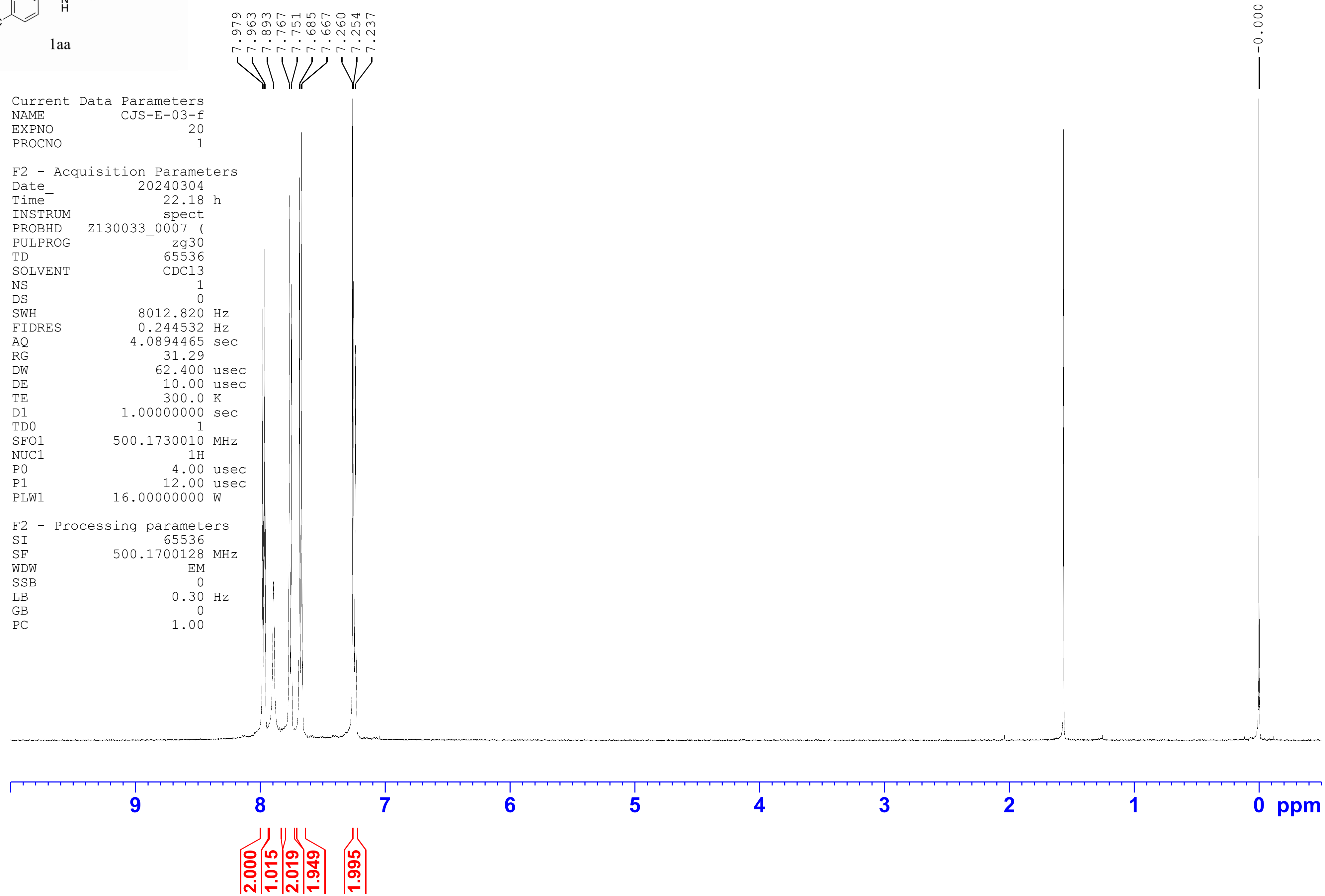


```
Current Data Parameters
NAME          CJS-E-03-f
EXPNO                 20
PROCNO                 1

F2 - Acquisition Parameters
Date_           20240304
Time               22.18 h
INSTRUM            spect
PROBHD   Z130033_0007 (
PULPROG             zg30
TD                 65536
SOLVENT            CDCl3
NS                     1
DS                     0
SWH             8012.820 Hz
FIDRES          0.244532 Hz
AQ             4.0894465 sec
RG                 31.29
DW                62.400 usec
DE                 10.00 usec
TE                 300.0 K
D1            1.00000000 sec
TD0                    1
SFO1         500.1730010 MHz
NUC1                  1H
P0                  4.00 usec
P1                 12.00 usec
PLW1         16.00000000 W

F2 - Processing parameters
SI                 65536
SF           500.1700128 MHz
WDW                   EM
SSB                    0
LB                  0.30 Hz
GB                     0
PC                  1.00
```

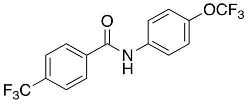

1aa

Current Data Parameters
NAME CJS-E-03-f
EXPNO 21
PROCNO 1

F2 - Acquisition Parameters
Date_ 20240304
Time 23.11 h
INSTRUM spect
PROBHD Z130033_0007 (
PULPROG zgpg30
TD 65536
SOLVENT CDCl3
NS 1024
DS 0
SWH 29761.904 Hz
FIDRES 0.908261 Hz
AQ 1.1010048 sec
RG 189.66
DW 16.800 usec
DE 11.00 usec
TE 300.0 K
D1 1.89900005 sec
D11 0.03000000 sec
TD0 1
SFO1 125.7804223 MHz
NUC1 13C
P0 3.33 usec
P1 10.00 usec
PLW1 70.00000000 W
SFO2 500.1720007 MHz
NUC2 1H
CPDPRG[2 waltz16
PCPD2 80.00 usec
PLW2 16.00000000 W
PLW12 0.36000001 W
PLW13 0.18108000 W

F2 - Processing parameters
SI 32768
SF 125.7678463 MHz
WDW EM
SSB 0
LB 1.00 Hz
GB 0
PC 1.40

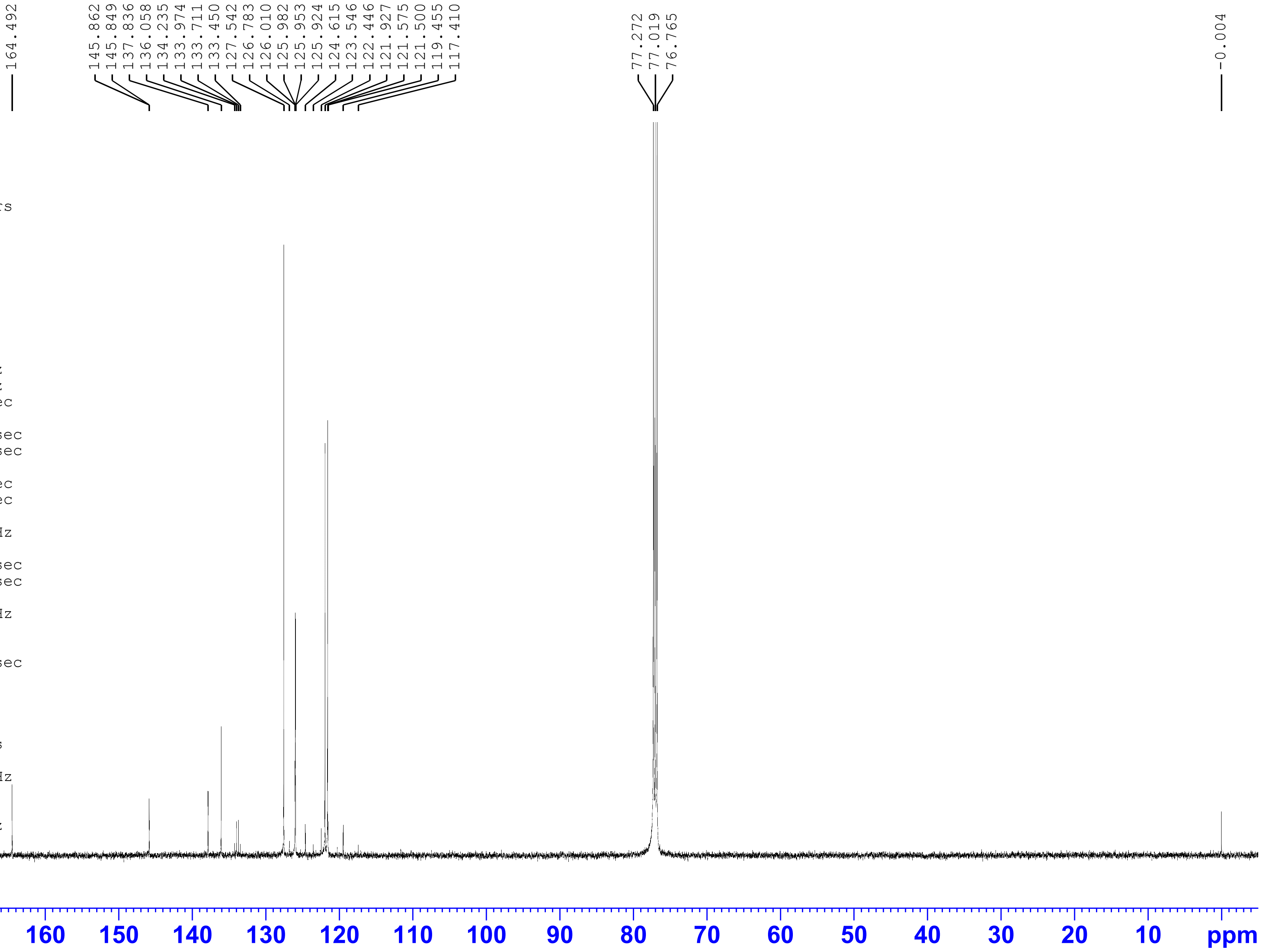

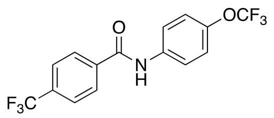

1aa

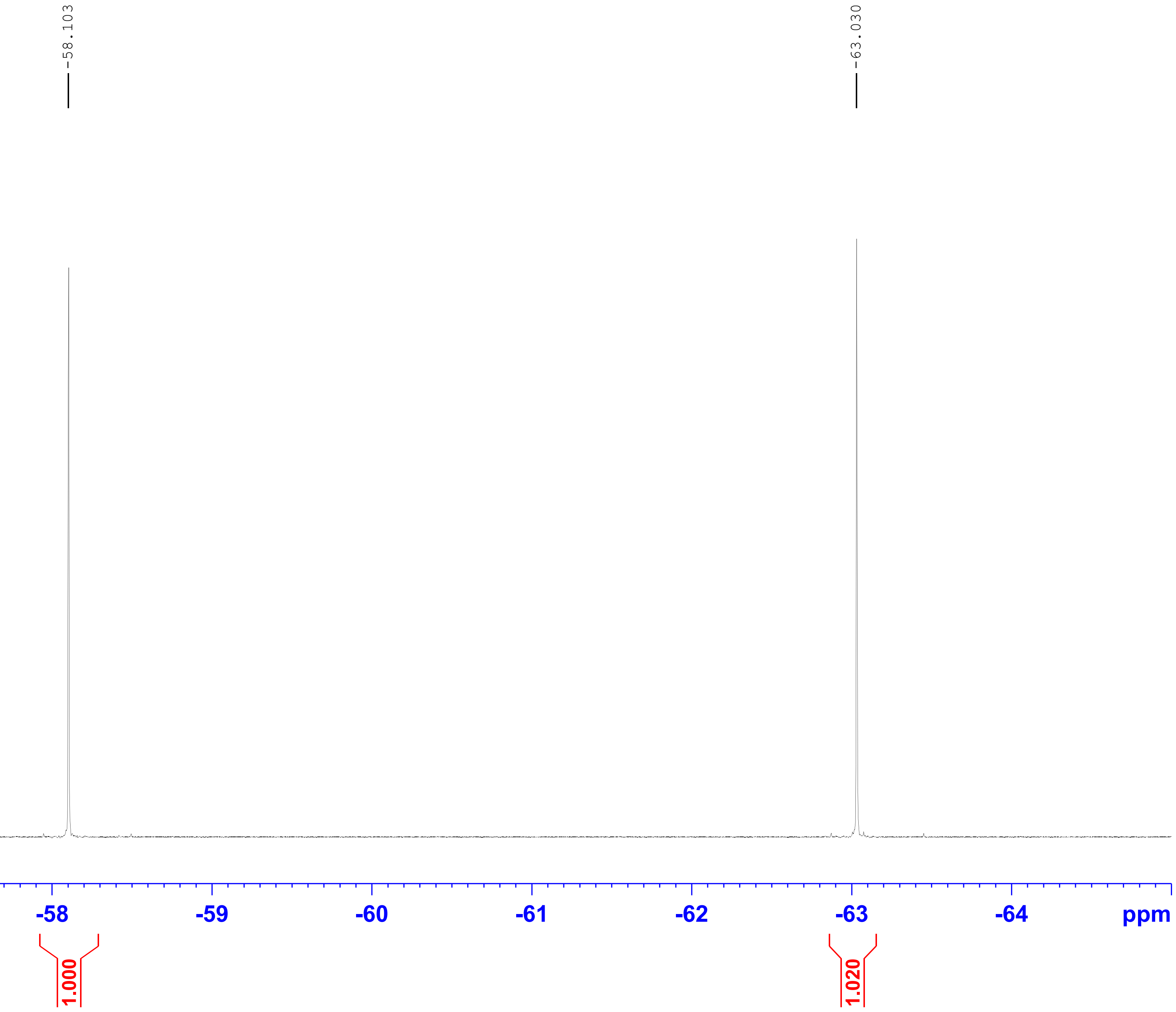


```
Current Data Parameters
NAME      CJS-E-03-nasi-3tube
EXPNO                  20
PROCNO                  1

F2 - Acquisition Parameters
Date_            20220928
Time                15.00 h
INSTRUM             spect
PROBHD   Z130033_0007 (
PULPROG            zgflqn
TD                 909078
SOLVENT             CDCl3
NS                      1
DS                      0
SWH            113636.367 Hz
FIDRES           0.250004 Hz
AQ              3.9999433 sec
RG                 189.66
DW                  4.400 usec
DE                 100.00 usec
TE                  300.0 K
D1             1.00000000 sec
TD0                     1
SFO1          470.5829520 MHz
NUC1                  19F
P1                  15.00 usec
PLW1          15.10000038 W

F2 - Processing parameters
SI                  65536
SF            470.6300150 MHz
WDW                    EM
SSB                     0
LB                   0.30 Hz
GB                      0
PC                   1.00
```

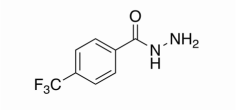

2a

Current Data Parameters
NAME CJS-E-03-414-4
EXPNO 11
PROCNO 1

F2 - Acquisition Parameters
Date_ 20231123
Time 21.28 h
INSTRUM spect
PROBHD Z130033_0007 (
PULPROG zg30
TD 65536
SOLVENT CDCl3
NS 1
DS 0
SWH 8012.820 Hz
FIDRES 0.244532 Hz
AQ 4.0894465 sec
RG 31.29
DW 62.400 usec
DE 10.00 usec
TE 300.0 K
D1 1.00000000 sec
TD0 1
SFO1 500.1730010 MHz
NUC1 1H
P0 4.00 usec
P1 12.00 usec
PLW1 16.00000000 W

F2 - Processing parameters
SI 65536
SF 500.1700109 MHz
WDW EM
SSB 0
LB 0.30 Hz
GB 0
PC 1.00

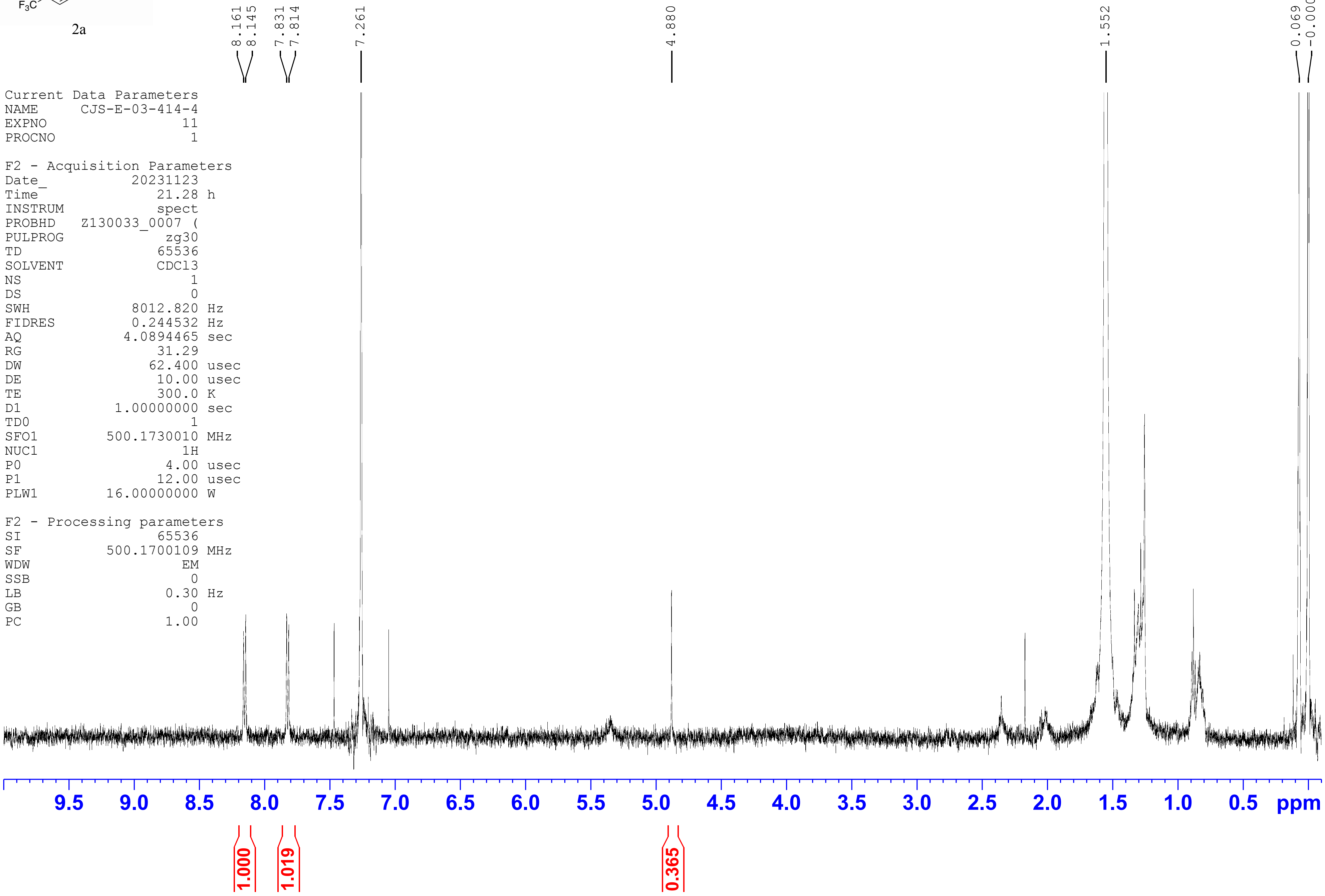

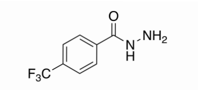

2a

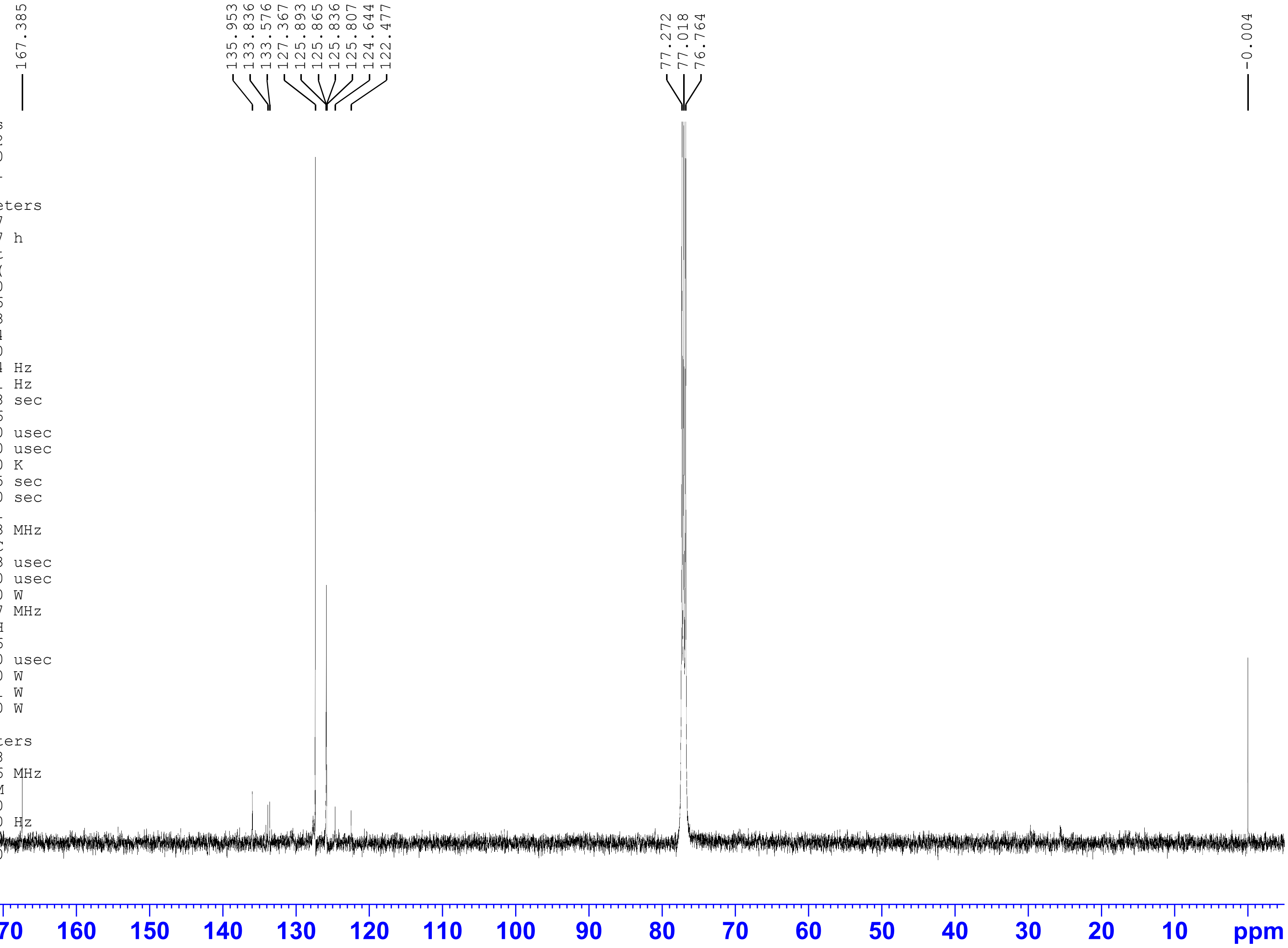


```
Current Data Parameters
NAME      CJS-e-03-32-2
EXPNO                20
PROCNO                1

F2 - Acquisition Parameters
Date_          20231217
Time              16.27 h
INSTRUM           spect
PROBHD   Z130033_0007 (
PULPROG          zgpg30
TD                65536
SOLVENT           CDCl3
NS                 1024
DS                    0
SWH           29761.904 Hz
FIDRES         0.908261 Hz
AQ            1.1010048 sec
RG               189.66
DW               16.800 usec
DE                11.00 usec
TE                300.0 K
D1           1.89900005 sec
D11          0.03000000 sec
TD0                   1
SFO1        125.7804223 MHz
NUC1                13C
P0                 3.33 usec
P1                10.00 usec
PLW1        70.00000000 W
SFO2        500.1720007 MHz
NUC2                 1H
CPDPRG[2        waltz16
PCPD2             80.00 usec
PLW2        16.00000000 W
PLW12        0.36000001 W
PLW13        0.18108000 W

F2 - Processing parameters
SI                32768
SF          125.7678466 MHz
WDW                  EM
SSB                   0
LB                 1.00 Hz
GB                    0
PC                 1.40
```

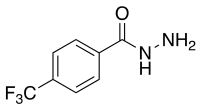

2a

Current Data Parameters
NAME CJS-E-03-414-4
EXPNO 10
PROCNO 1

F2 - Acquisition Parameters
Date_ 20231123
Time 21.26 h
INSTRUM spect
PROBHD Z130033_0007 (
PULPROG zgflqn
TD 909078
SOLVENT CDCl3
NS 1
DS 0
SWH 113636.367 Hz
FIDRES 0.250004 Hz
AQ 3.9999433 sec
RG 189.66
DW 4.400 usec
DE 100.00 usec
TE 300.0 K
D1 1.00000000 sec
TD0 1
SFO1 470.5829516 MHz
NUC1 19F
P1 15.00 usec
PLW1 15.10000038 W

F2 - Processing parameters
SI 65536
SF 470.6300150 MHz
WDW EM
SSB 0
LB 0.30 Hz
GB 0
PC 1.00

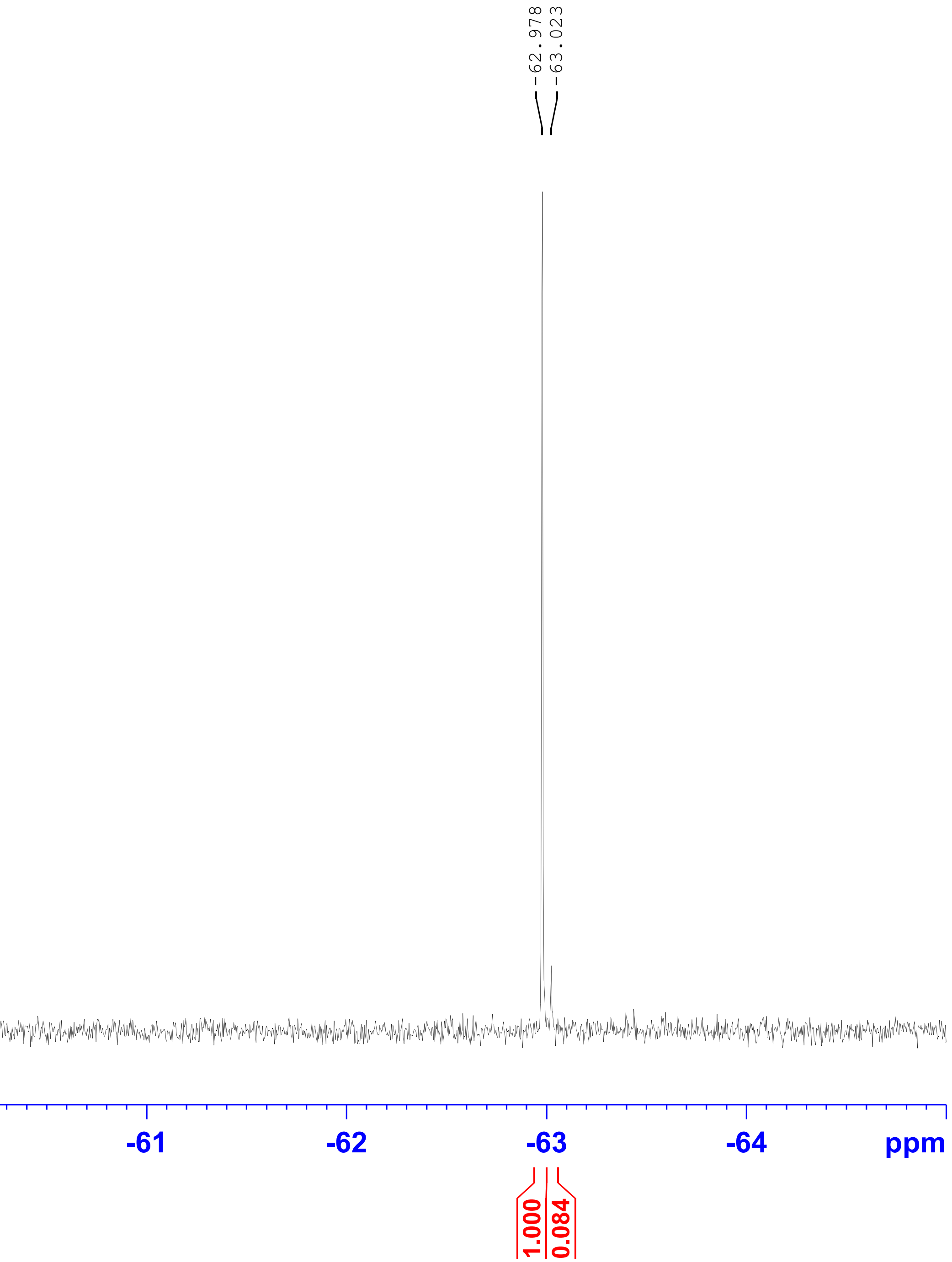

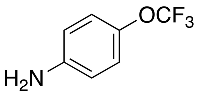

3a

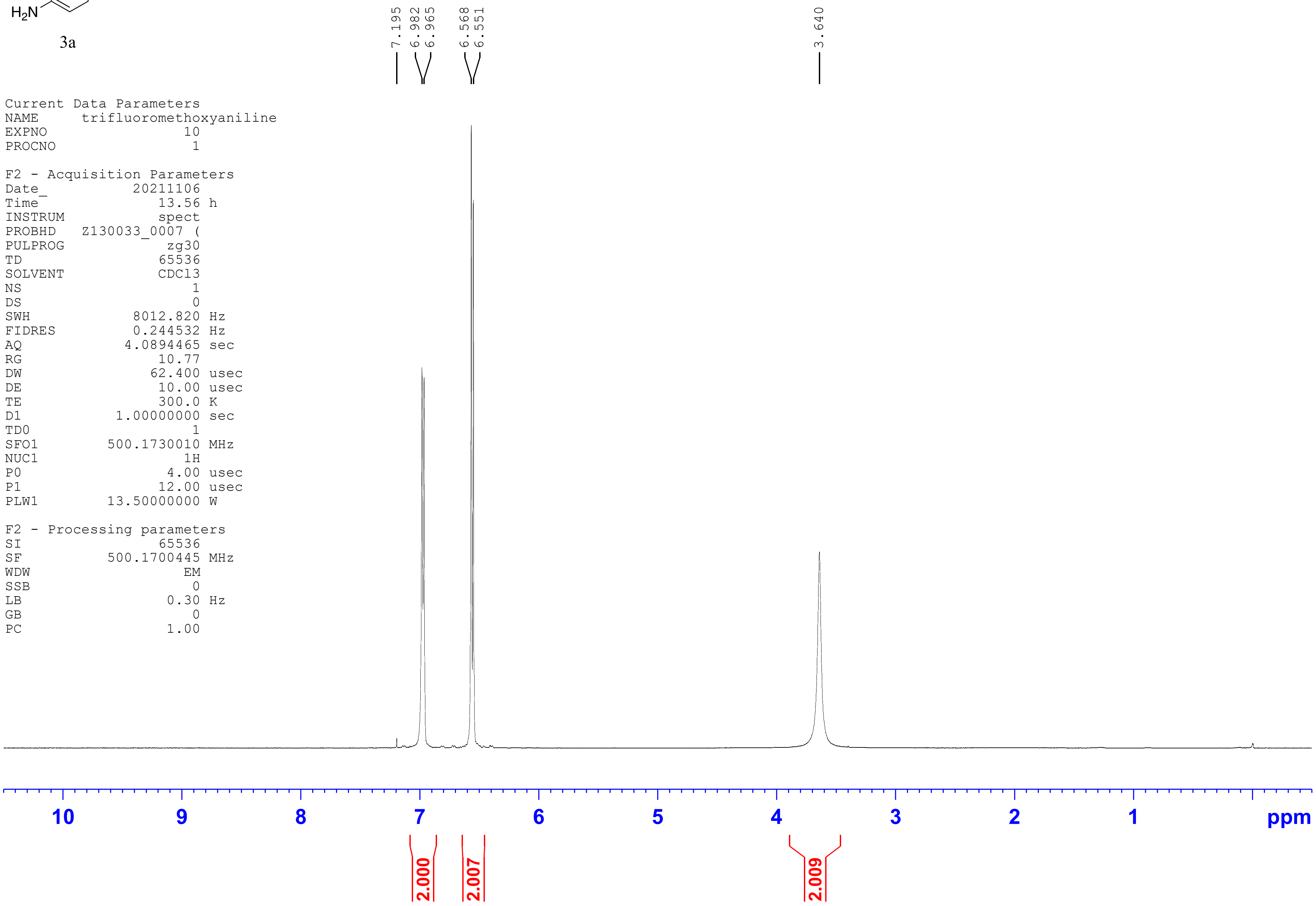


```
Current Data Parameters
NAME      trifluoromethoxyaniline
EXPNO                    10
PROCNO                    1

F2 - Acquisition Parameters
Date_              20211106
Time                  13.56 h
INSTRUM               spect
PROBHD    Z130033_0007 (
PULPROG                zg30
TD                    65536
SOLVENT               CDCl3
NS                        1
DS                        0
SWH                8012.820 Hz
FIDRES             0.244532 Hz
AQ                4.0894465 sec
RG                    10.77
DW                   62.400 usec
DE                    10.00 usec
TE                    300.0 K
D1               1.00000000 sec
TD0                       1
SFO1            500.1730010 MHz
NUC1                     1H
P0                     4.00 usec
P1                    12.00 usec
PLW1            13.50000000 W

F2 - Processing parameters
SI                    65536
SF              500.1700445 MHz
WDW                      EM
SSB                       0
LB                     0.30 Hz
GB                        0
PC                     1.00
```

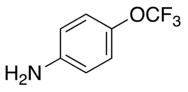

3a

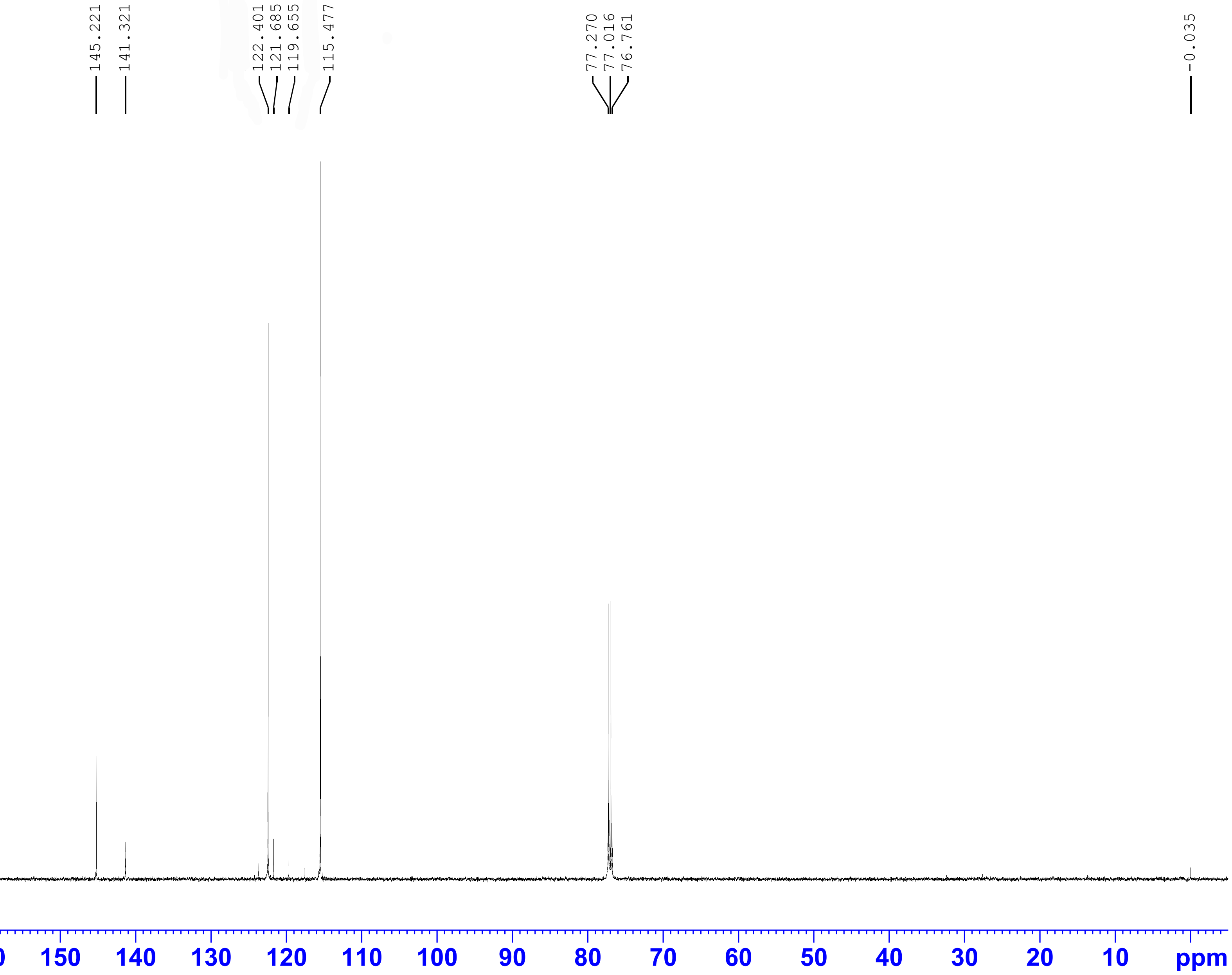


```
Current Data Parameters
NAME     CJS-E-03-f-aniline
EXPNO                  10
PROCNO                  1

F2 - Acquisition Parameters
Date_            20231202
Time                19.50 h
INSTRUM             spect
PROBHD   Z130033_0007 (
PULPROG            zgpg30
TD                  65536
SOLVENT             CDCl3
NS                    128
DS                      0
SWH             29761.904 Hz
FIDRES           0.908261 Hz
AQ              1.1010048 sec
RG                 189.66
DW                 16.800 usec
DE                  11.00 usec
TE                  300.0 K
D1             1.89900005 sec
D11            0.03000000 sec
TD0                     1
SFO1          125.7804223 MHz
NUC1                  13C
P0                   3.33 usec
P1                  10.00 usec
PLW1          70.00000000 W
SFO2          500.1720007 MHz
NUC2                   1H
CPDPRG[2          waltz16
PCPD2               80.00 usec
PLW2          16.00000000 W
PLW12          0.36000001 W
PLW13          0.18108000 W

F2 - Processing parameters
SI                  32768
SF            125.7678470 MHz
WDW                    EM
SSB                     0
LB                   1.00 Hz
GB                      0
PC                   1.40
```

Current Data Parameters
NAME 4-trifluoromethoxylaniline
EXPNO 10
PROCNO 1

F2 - Acquisition Parameters

Date_	20211113	
Time	19.36	h
INSTRUM	spect	
PROBHD	Z130033_0007 (	
PULPROG	zgflqn	
TD	909078	
SOLVENT	CDCl3	
NS	1	
DS	0	
SWH	113636.367	Hz
FIDRES	0.250004	Hz
AQ	3.9999433	sec
RG	189.66	
DW	4.400	usec
DE	100.00	usec
TE	300.0	K
D1	1.00000000	sec
TD0	1	
SFO1	470.5829520	MHz
NUC1	19F	
P1	15.00	usec
PLW1	15.10000038	W

F2 - Processing parameters

SI	65536	
SF	470.6300150	MHz
WDW	EM	
SSB	0	
LB	0.30	Hz
GB	0	
PC	1.00	

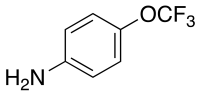

3a

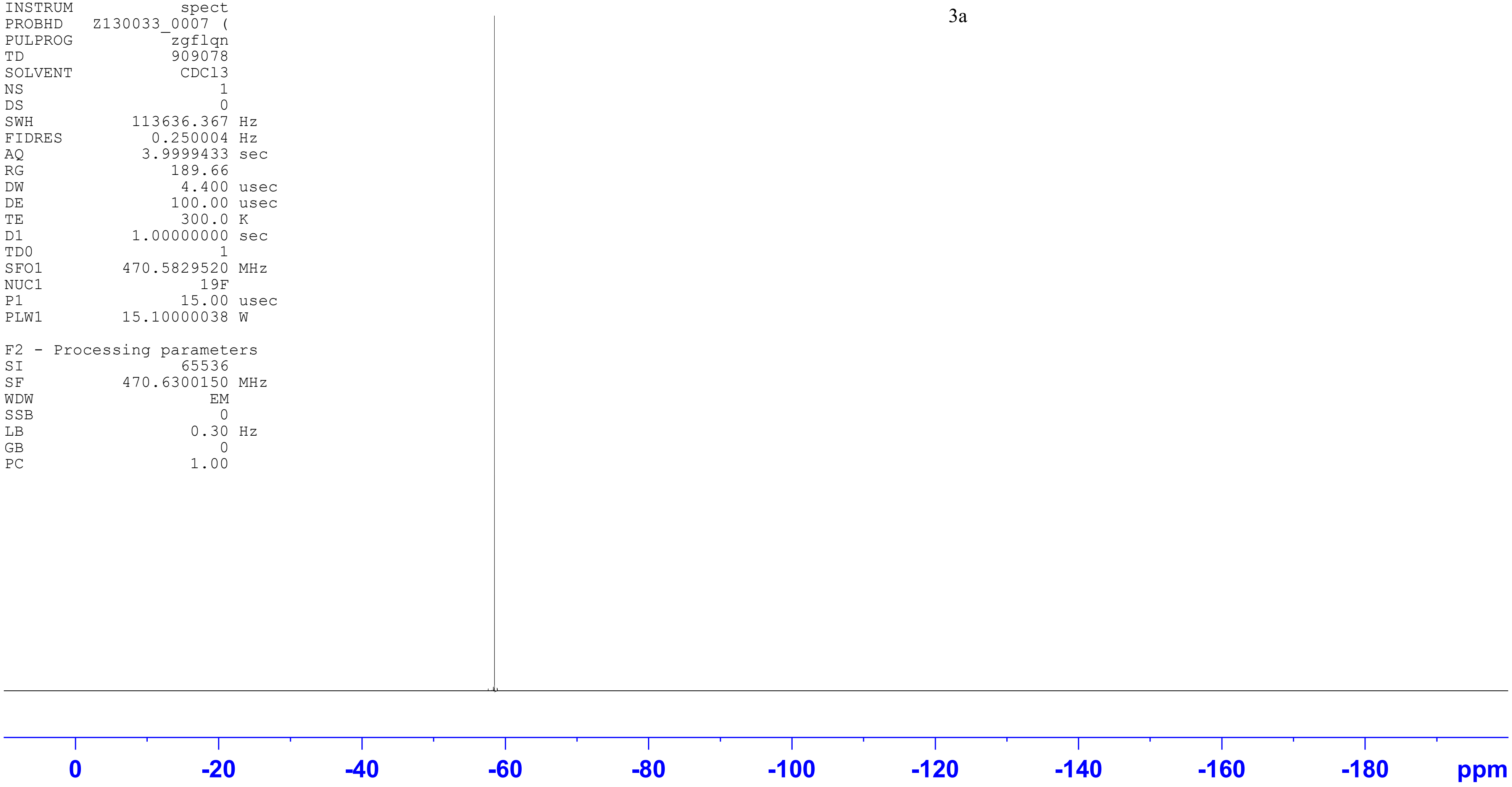

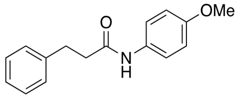

1bb

Current Data Parameters
NAME CJS-E-03-237-SM
EXPNO 10
PROCNO 1

F2 - Acquisition Parameters
Date_ 20221118
Time 10.40 h
INSTRUM spect
PROBHD Z130033_0007 (
PULPROG zg30
TD 65536
SOLVENT CDCl3
NS 16
DS 2
SWH 10000.000 Hz
FIDRES 0.305176 Hz
AQ 3.2767999 sec
RG 54.54
DW 50.000 usec
DE 13.55 usec
TE 300.0 K
D1 1.00000000 sec
TD0 1
SFO1 500.1730885 MHz
NUC1 1H
P0 4.00 usec
P1 12.00 usec
PLW1 13.50000000 W

F2 - Processing parameters
SI 65536
SF 500.1700132 MHz
WDW EM
SSB 0
LB 0.30 Hz
GB 0
PC 1.00

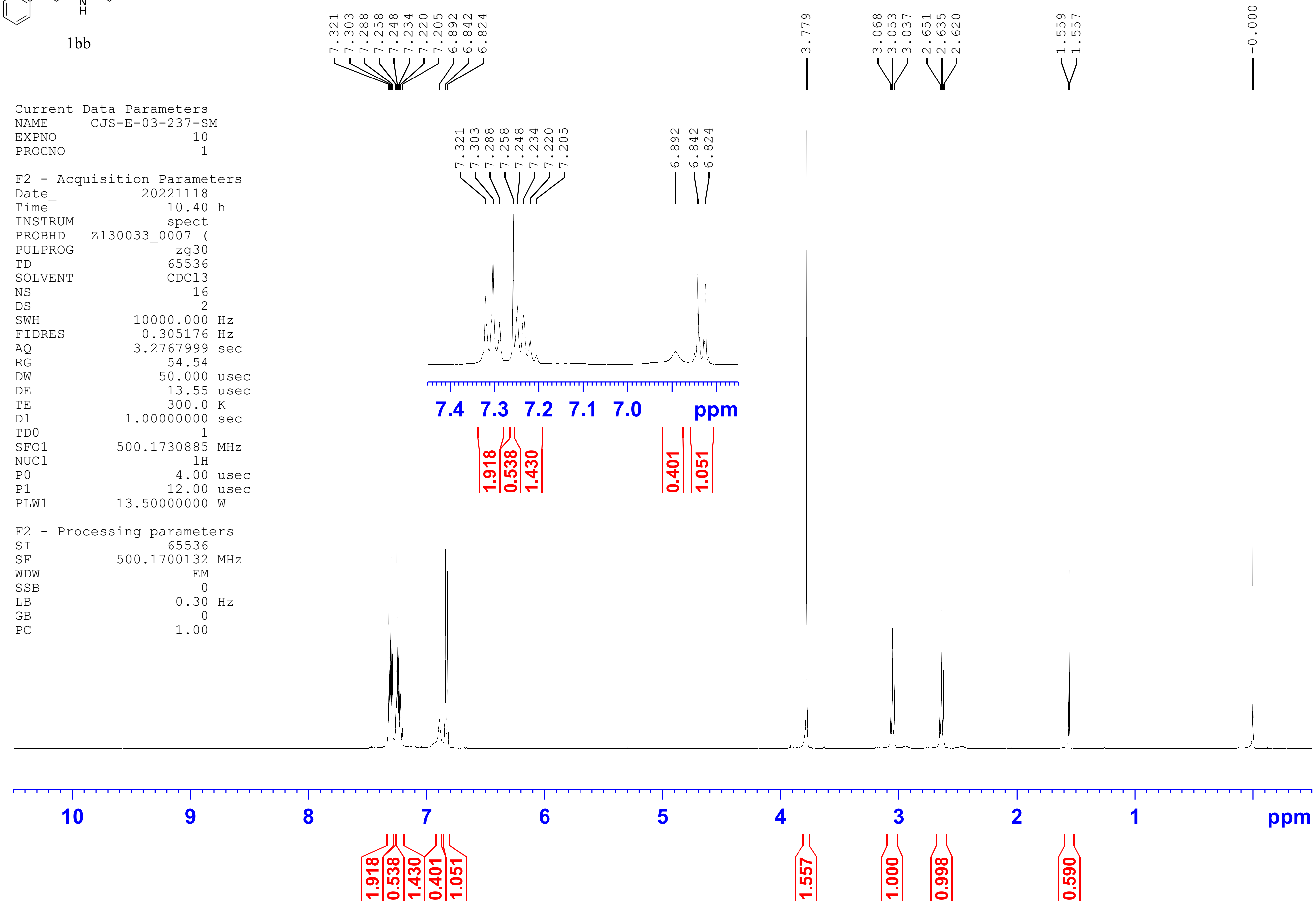

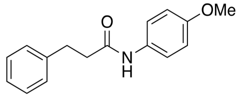

1bb

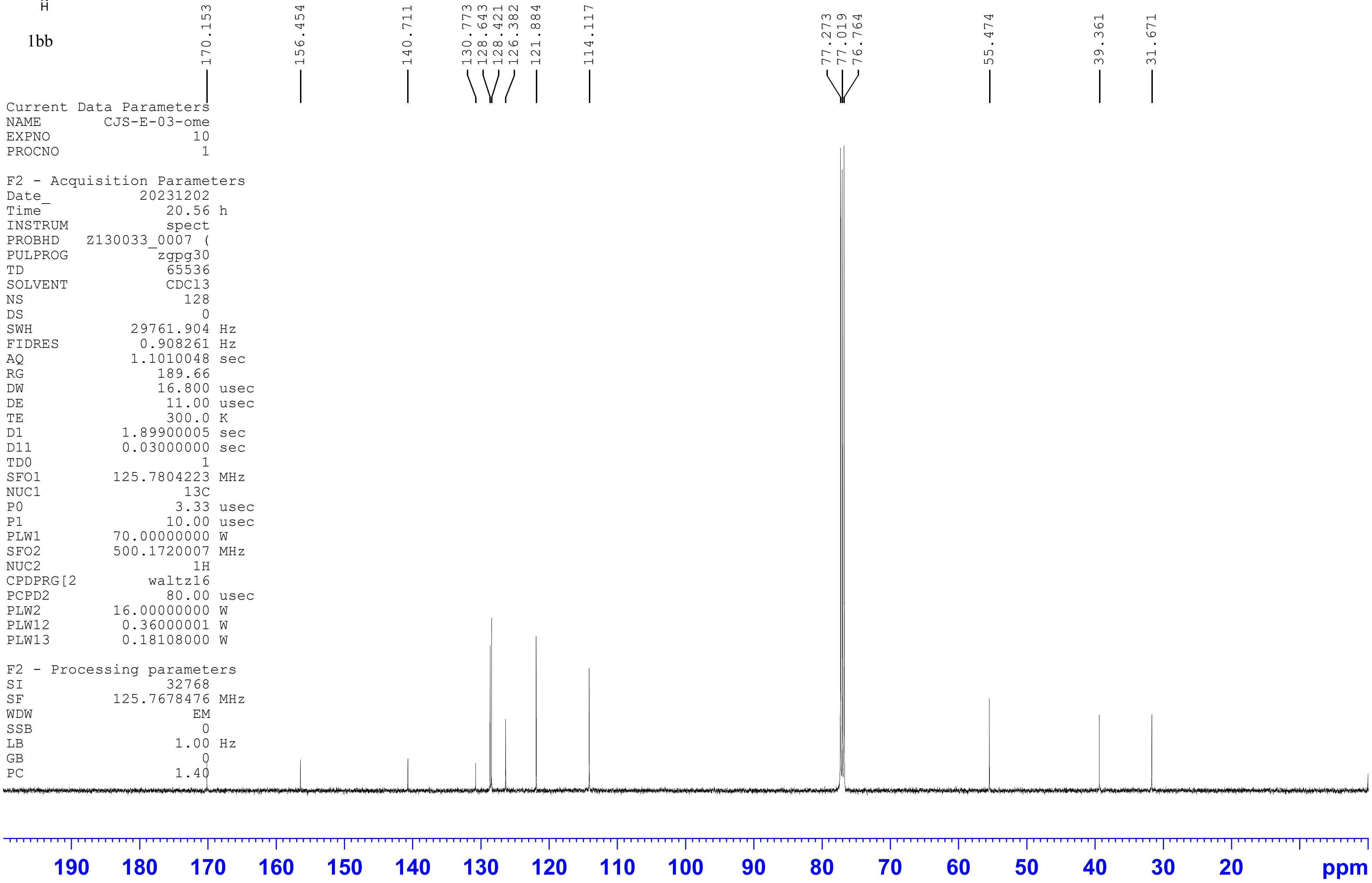

Current Data Parameters
NAME CJS-E-03-ome
EXPNO 10
PROCNO 1

F2 - Acquisition Parameters
Date_ 20231202
Time 20.56 h
INSTRUM spect
PROBHD Z130033_0007 (
PULPROG zgpg30
TD 65536
SOLVENT CDCl3
NS 128
DS 0
SWH 29761.904 Hz
FIDRES 0.908261 Hz
AQ 1.1010048 sec
RG 189.66
DW 16.800 usec
DE 11.00 usec
TE 300.0 K
D1 1.89900005 sec
D11 0.03000000 sec
TD0 1
SFO1 125.7804223 MHz
NUC1 13C
P0 3.33 usec
P1 10.00 usec
PLW1 70.00000000 W
SFO2 500.1720007 MHz
NUC2 1H
CPDPRG[2 waltz16
PCPD2 80.00 usec
PLW2 16.00000000 W
PLW12 0.36000001 W
PLW13 0.18108000 W

F2 - Processing parameters
SI 32768
SF 125.7678476 MHz
WDW EM
SSB 0
LB 1.00 Hz
GB 0
PC 1.40

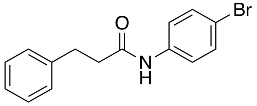

1bc

Current Data Parameters
NAME CJS-E-03-sm-br
EXPNO 10
PROCNO 1

F2 - Acquisition Parameters
Date_ 20230310
Time 14.35 h
INSTRUM spect
PROBHD Z130033_0007 (
PULPROG zg30
TD 65536
SOLVENT CDCl3
NS 1
DS 0
SWH 8012.820 Hz
FIDRES 0.244532 Hz
AQ 4.0894465 sec
RG 31.29
DW 62.400 usec
DE 10.00 usec
TE 300.0 K
D1 1.00000000 sec
TD0 1
SFO1 500.1730010 MHz
NUC1 1H
P0 4.00 usec
P1 12.00 usec
PLW1 13.50000000 W

F2 - Processing parameters
SI 65536
SF 500.1700131 MHz
WDW EM
SSB 0
LB 0.30 Hz
GB 0
PC 1.00

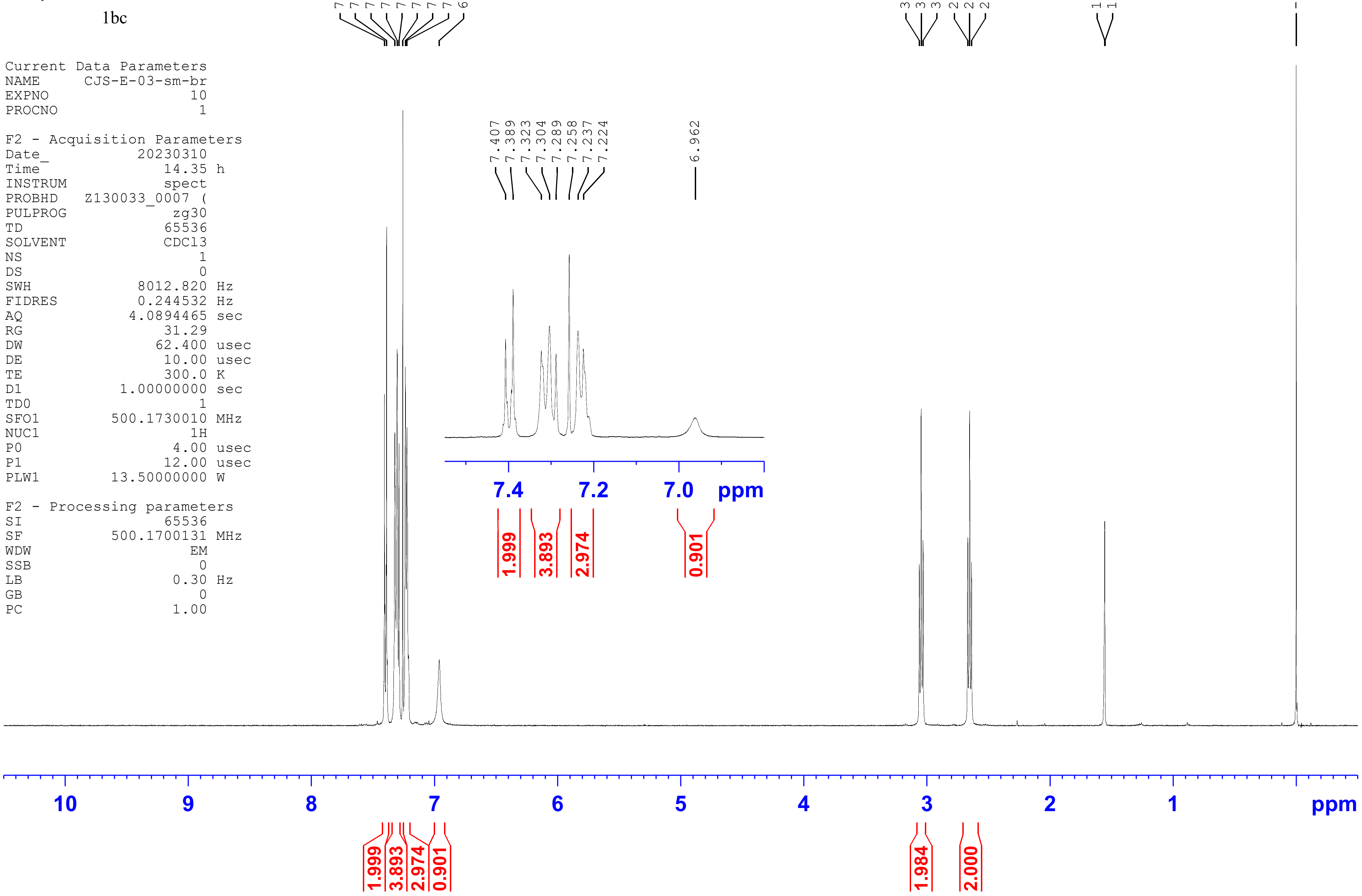

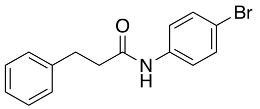

1bc

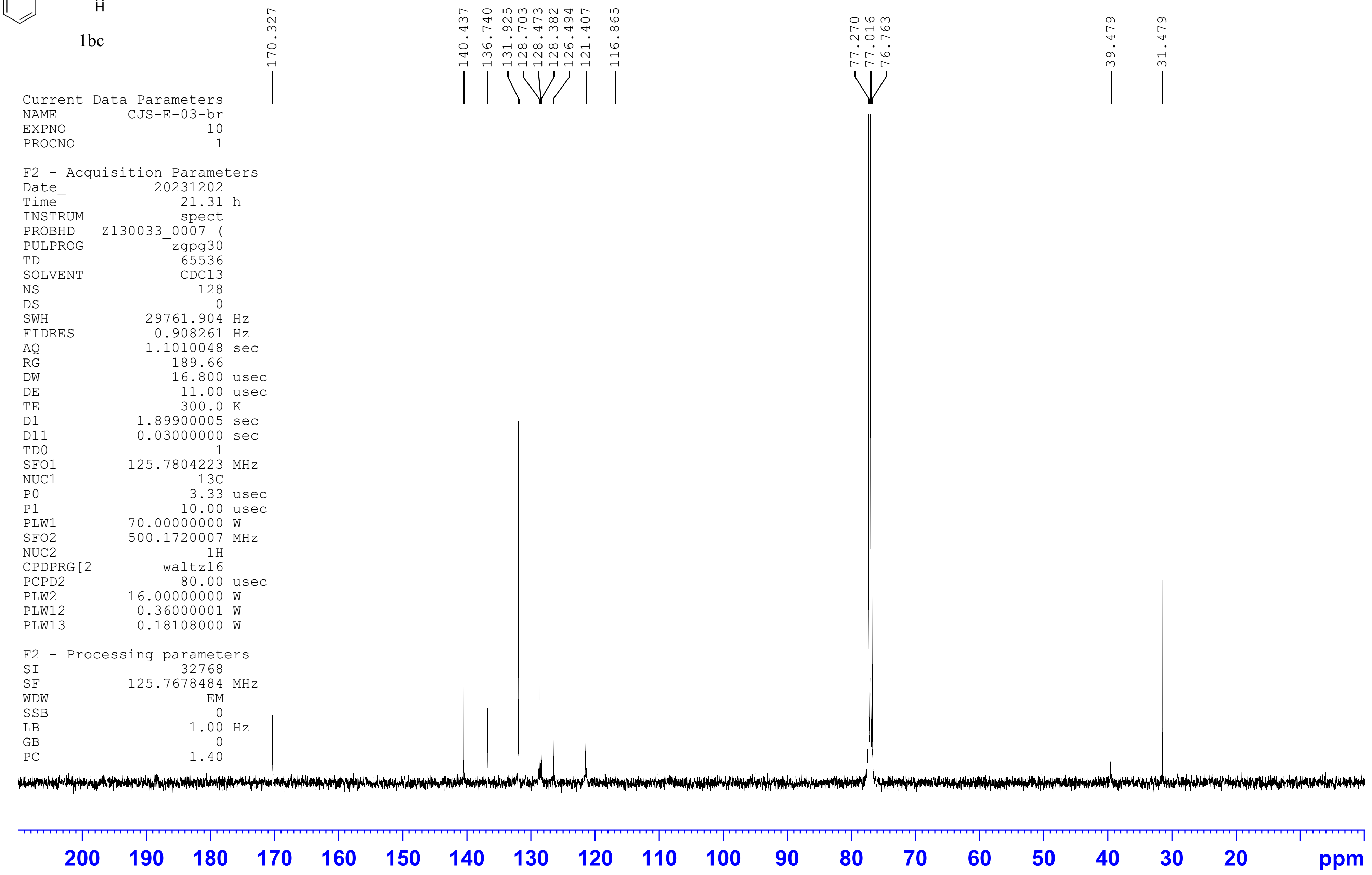


```
Current Data Parameters
NAME         CJS-E-03-br
EXPNO                 10
PROCNO                 1

F2 - Acquisition Parameters
Date_           20231202
Time               21.31 h
INSTRUM            spect
PROBHD    Z130033_0007 (
PULPROG          zgpg30
TD                 65536
SOLVENT            CDCl3
NS                   128
DS                     0
SWH            29761.904 Hz
FIDRES          0.908261 Hz
AQ             1.1010048 sec
RG                189.66
DW                16.800 usec
DE                 11.00 usec
TE                 300.0 K
D1            1.89900005 sec
D11           0.03000000 sec
TD0                    1
SFO1         125.7804223 MHz
NUC1                 13C
P0                  3.33 usec
P1                 10.00 usec
PLW1         70.00000000 W
SFO2         500.1720007 MHz
NUC2                  1H
CPDPRG[2         waltz16
PCPD2              80.00 usec
PLW2         16.00000000 W
PLW12         0.36000001 W
PLW13         0.18108000 W

F2 - Processing parameters
SI                 32768
SF           125.7678484 MHz
WDW                   EM
SSB                    0
LB                  1.00 Hz
GB                     0
PC                  1.40
```

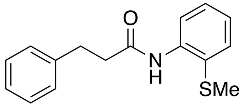

1bd

```
Current Data Parameters
NAME          CJS-E-03-SMe
EXPNO                   10
PROCNO                   1

F2 - Acquisition Parameters
Date_             20230519
Time                 16.26 h
INSTRUM              spect
PROBHD   Z119470_0344 (
PULPROG               zg30
TD                   65536
SOLVENT              CDCl3
NS                       1
DS                       0
SWH               8012.820 Hz
FIDRES            0.244532 Hz
AQ               4.0894465 sec
RG                  130.52
DW                  62.400 usec
DE                    6.50 usec
TE                   298.0 K
D1              1.00000000 sec
TD0                      1
SFO1           500.1730010 MHz
NUC1                    1H
P0                    4.83 usec
P1                   14.50 usec
PLW1           10.80000019 W

F2 - Processing parameters
SI                   65536
SF             500.1700136 MHz
WDW                     EM
SSB                      0
LB                    0.30 Hz
GB                       0
PC                    1.00
```

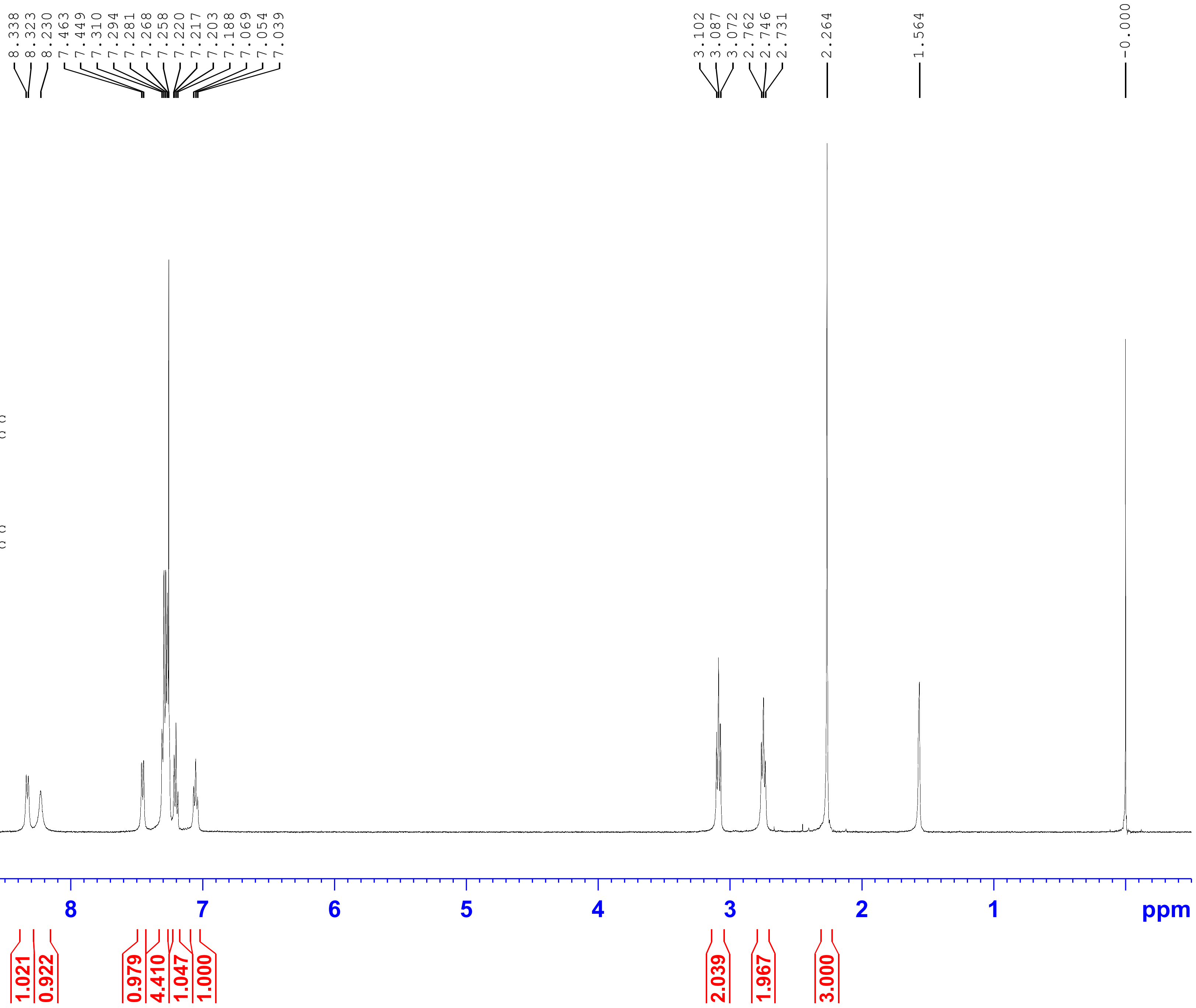

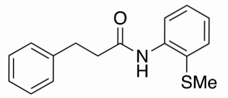

1bd

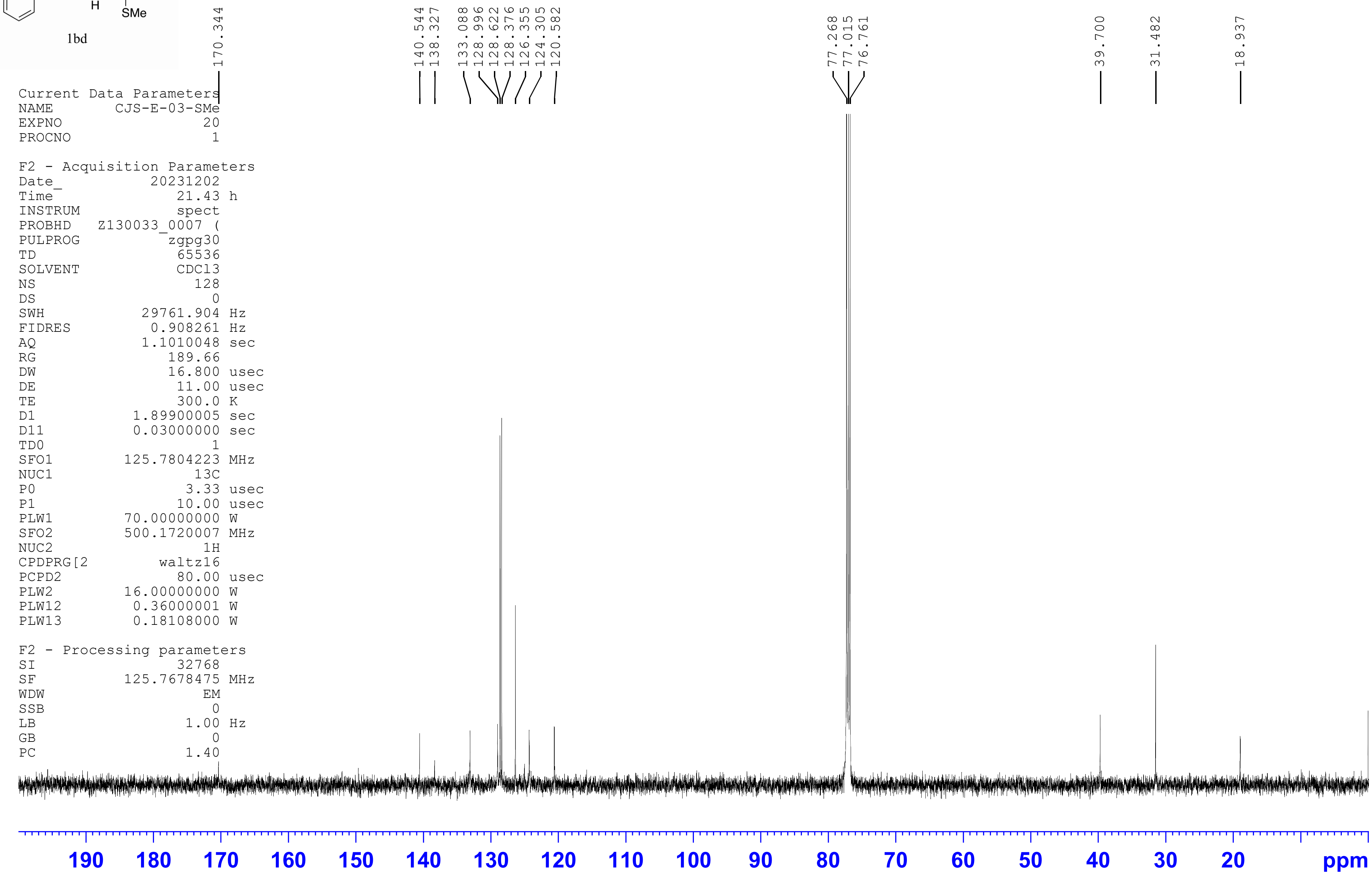

Current Data Parameters
NAME CJS-E-03-SMe
EXPNO 20
PROCNO 1

F2 - Acquisition Parameters
Date_ 20231202
Time 21.43 h
INSTRUM spect
PROBHD Z130033_0007 (
PULPROG zgpg30
TD 65536
SOLVENT CDCl3
NS 128
DS 0
SWH 29761.904 Hz
FIDRES 0.908261 Hz
AQ 1.1010048 sec
RG 189.66
DW 16.800 usec
DE 11.00 usec
TE 300.0 K
D1 1.89900005 sec
D11 0.03000000 sec
TD0 1
SFO1 125.7804223 MHz
NUC1 13C
P0 3.33 usec
P1 10.00 usec
PLW1 70.00000000 W
SFO2 500.1720007 MHz
NUC2 1H
CPDPRG[2 waltz16
PCPD2 80.00 usec
PLW2 16.00000000 W
PLW12 0.36000001 W
PLW13 0.18108000 W

F2 - Processing parameters
SI 32768
SF 125.7678475 MHz
WDW EM
SSB 0
LB 1.00 Hz
GB 0
PC 1.40

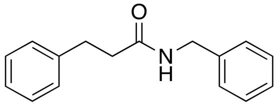

1be

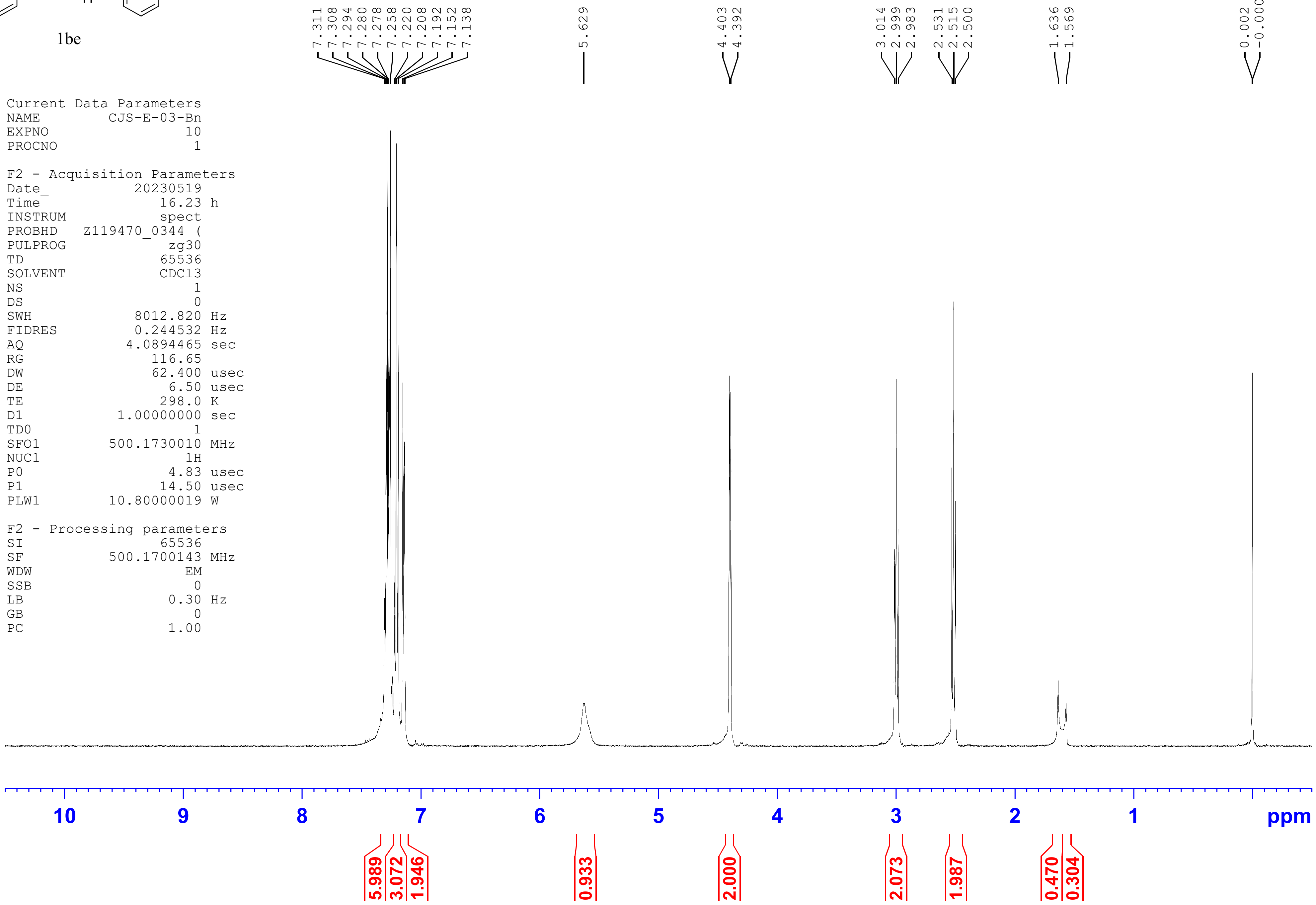

Current Data Parameters
NAME CJS-E-03-Bn
EXPNO 10
PROCNO 1

F2 - Acquisition Parameters
Date_ 20230519
Time 16.23 h
INSTRUM spect
PROBHD Z119470_0344 (
PULPROG zg30
TD 65536
SOLVENT CDCl3
NS 1
DS 0
SWH 8012.820 Hz
FIDRES 0.244532 Hz
AQ 4.0894465 sec
RG 116.65
DW 62.400 usec
DE 6.50 usec
TE 298.0 K
D1 1.00000000 sec
TD0 1
SFO1 500.1730010 MHz
NUC1 1H
P0 4.83 usec
P1 14.50 usec
PLW1 10.80000019 W

F2 - Processing parameters
SI 65536
SF 500.1700143 MHz
WDW EM
SSB 0
LB 0.30 Hz
GB 0
PC 1.00

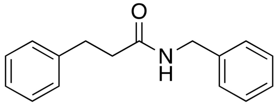

1be

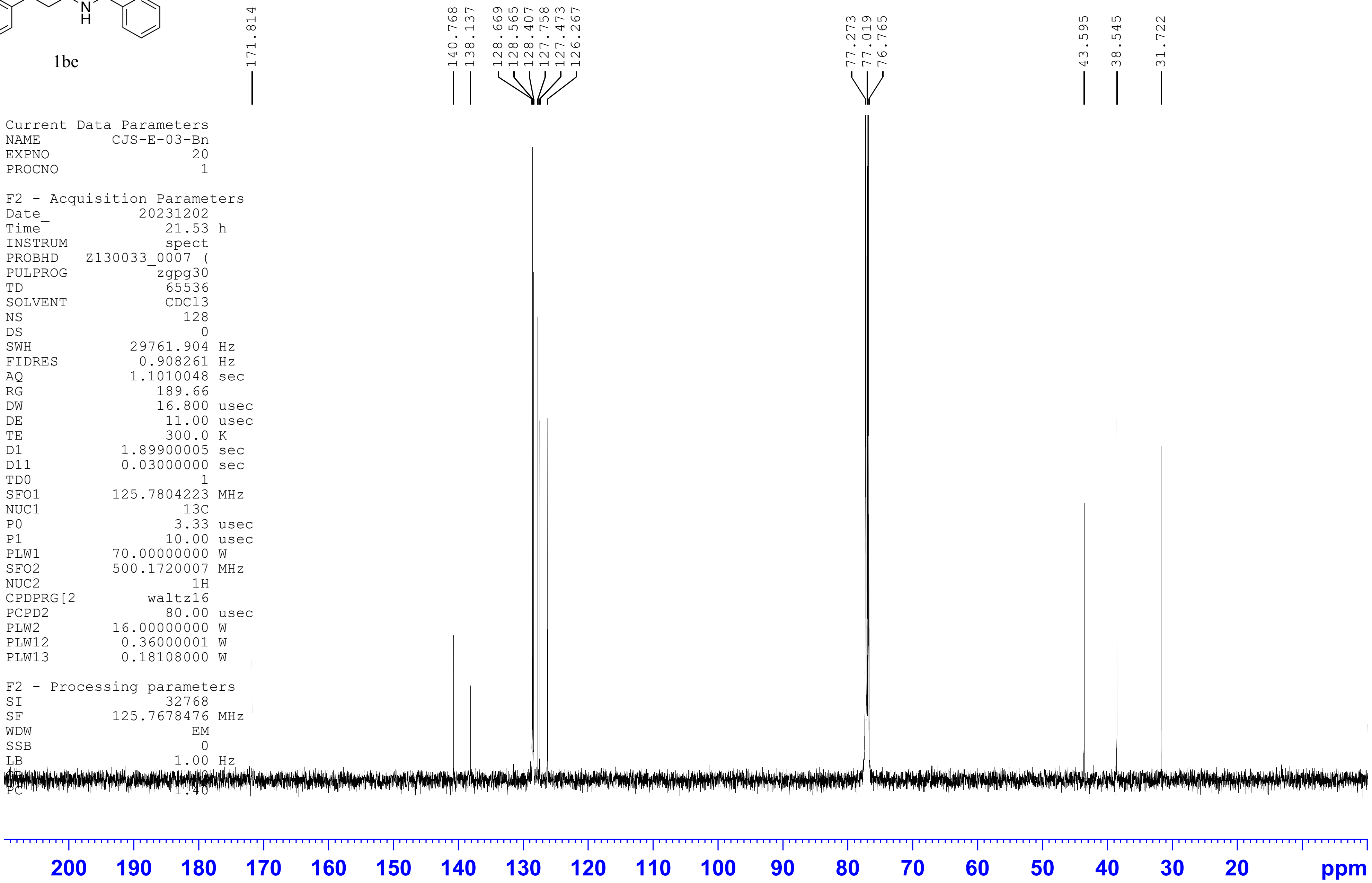


```
Current Data Parameters
NAME          CJS-E-03-Bn
EXPNO                  20
PROCNO                  1

F2 - Acquisition Parameters
Date_            20231202
Time                21.53 h
INSTRUM             spect
PROBHD   Z130033_0007 (
PULPROG           zgpg30
TD                 65536
SOLVENT            CDCl3
NS                   128
DS                     0
SWH            29761.904 Hz
FIDRES          0.908261 Hz
AQ             1.1010048 sec
RG                189.66
DW                16.800 usec
DE                 11.00 usec
TE                 300.0 K
D1            1.89900005 sec
D11           0.03000000 sec
TD0                    1
SFO1         125.7804223 MHz
NUC1                 13C
P0                  3.33 usec
P1                 10.00 usec
PLW1         70.00000000 W
SFO2         500.1720007 MHz
NUC2                  1H
CPDPRG[2         waltz16
PCPD2              80.00 usec
PLW2         16.00000000 W
PLW12         0.36000001 W
PLW13         0.18108000 W

F2 - Processing parameters
SI                 32768
SF           125.7678476 MHz
WDW                   EM
SSB                    0
LB                  1.00 Hz
GB                     0
PC                  1.40
```

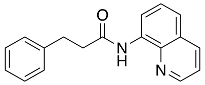

1bf

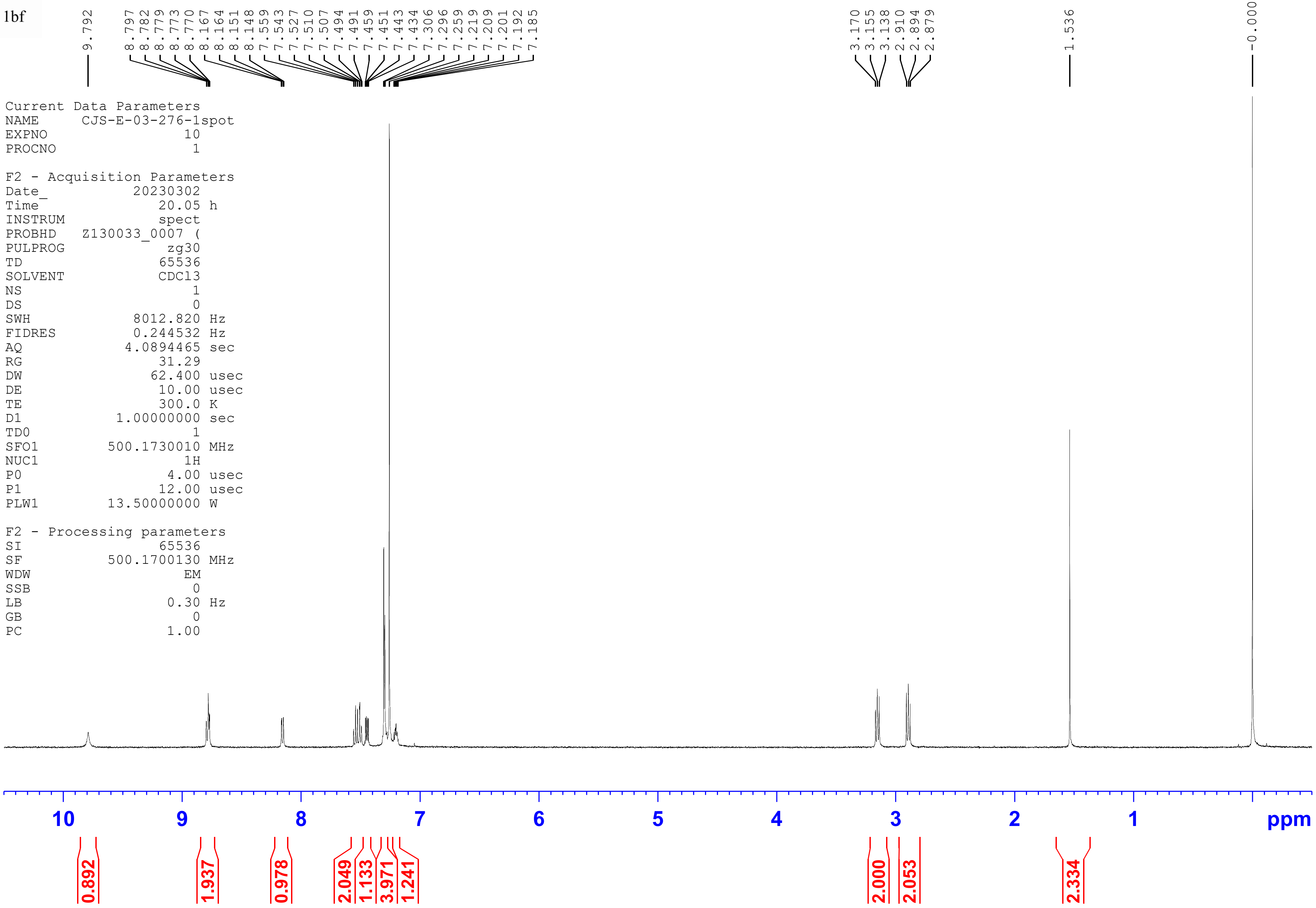


```
Current Data Parameters
NAME      CJS-E-03-276-1spot
EXPNO                   10
PROCNO                   1

F2 - Acquisition Parameters
Date_             20230302
Time                 20.05 h
INSTRUM              spect
PROBHD   Z130033_0007 (
PULPROG               zg30
TD                   65536
SOLVENT              CDCl3
NS                       1
DS                       0
SWH               8012.820 Hz
FIDRES            0.244532 Hz
AQ               4.0894465 sec
RG                   31.29
DW                  62.400 usec
DE                   10.00 usec
TE                   300.0 K
D1              1.00000000 sec
TD0                      1
SFO1           500.1730010 MHz
NUC1                    1H
P0                    4.00 usec
P1                   12.00 usec
PLW1           13.50000000 W

F2 - Processing parameters
SI                   65536
SF             500.1700130 MHz
WDW                     EM
SSB                      0
LB                    0.30 Hz
GB                       0
PC                    1.00
```

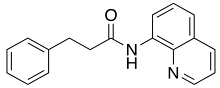

1bf

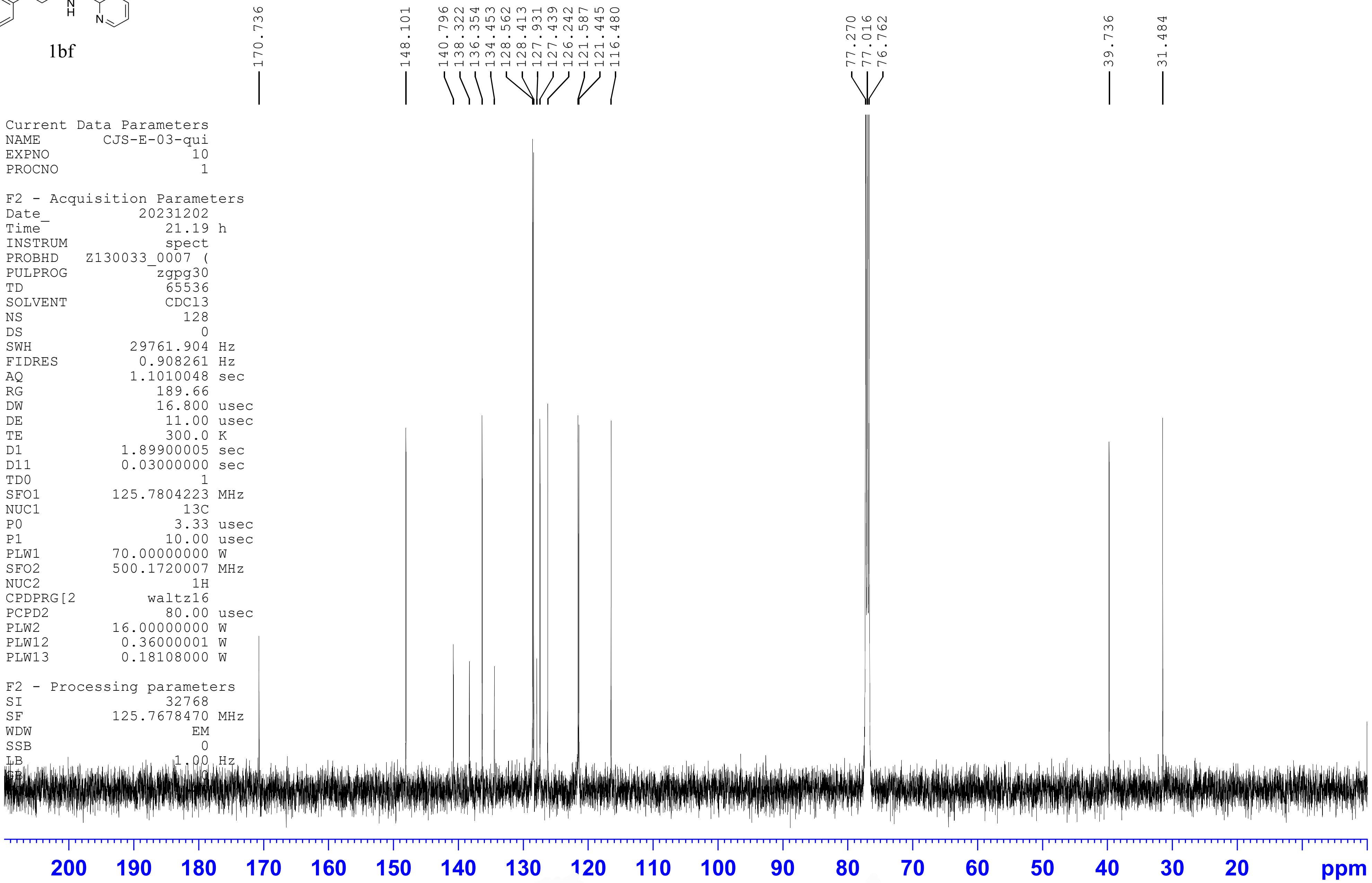

Current Data Parameters	
NAME	CJS-E-03-qui
EXPNO	10
PROCNO	1
F2 - Acquisition Parameters	
Date_	20231202
Time	21.19 h
INSTRUM	spect
PROBHD	Z130033_0007 (
PULPROG	zgpg30
TD	65536
SOLVENT	CDCl3
NS	128
DS	0
SWH	29761.904 Hz
FIDRES	0.908261 Hz
AQ	1.1010048 sec
RG	189.66
DW	16.800 usec
DE	11.00 usec
TE	300.0 K
D1	1.89900005 sec
D11	0.03000000 sec
TD0	1
SFO1	125.7804223 MHz
NUC1	13C
P0	3.33 usec
P1	10.00 usec
PLW1	70.00000000 W
SFO2	500.1720007 MHz
NUC2	1H
CPDPRG[2	waltz16
PCPD2	80.00 usec
PLW2	16.00000000 W
PLW12	0.36000001 W
PLW13	0.18108000 W
F2 - Processing parameters	
SI	32768
SF	125.7678470 MHz
WDW	EM
SSB	0
LB	1.00 Hz
GB	0

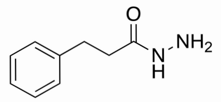

2b

Current Data Parameters
NAME CJS-E-03-525
EXPNO 20
PROCNO 1

F2 - Acquisition Parameters
Date_ 20240309
Time 11.53 h
INSTRUM spect
PROBHD Z130033_0007 (
PULPROG zg30
TD 65536
SOLVENT CDCl3
NS 1
DS 0
SWH 8012.820 Hz
FIDRES 0.244532 Hz
AQ 4.0894465 sec
RG 31.29
DW 62.400 usec
DE 10.00 usec
TE 300.0 K
D1 1.00000000 sec
TD0 1
SFO1 500.1730010 MHz
NUC1 1H
P0 4.00 usec
P1 12.00 usec
PLW1 16.00000000 W

F2 - Processing parameters
SI 65536
SF 500.1700121 MHz
WDW EM
SSB 0
LB 0.30 Hz
GB 0
PC 1.00

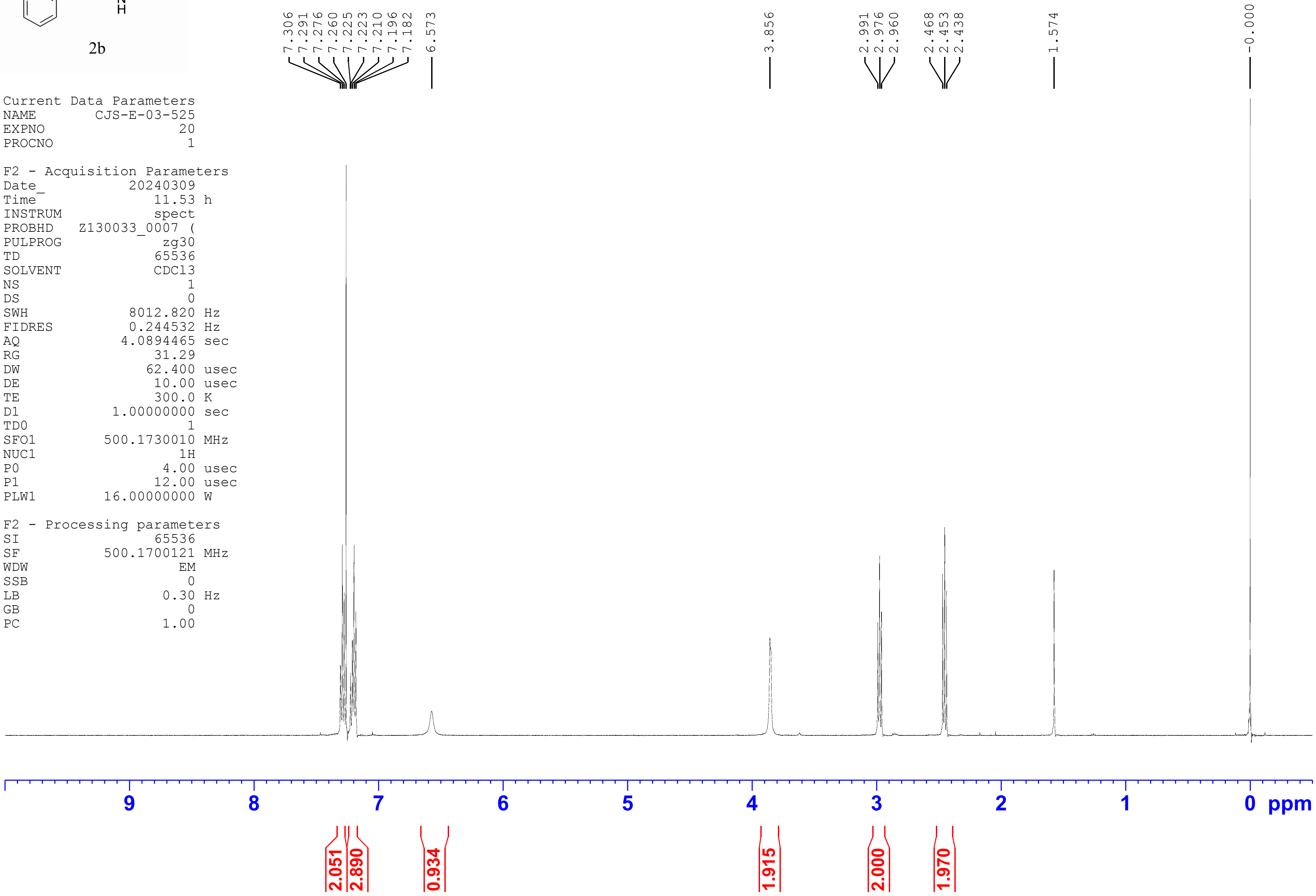

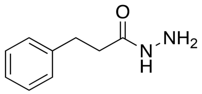

2b

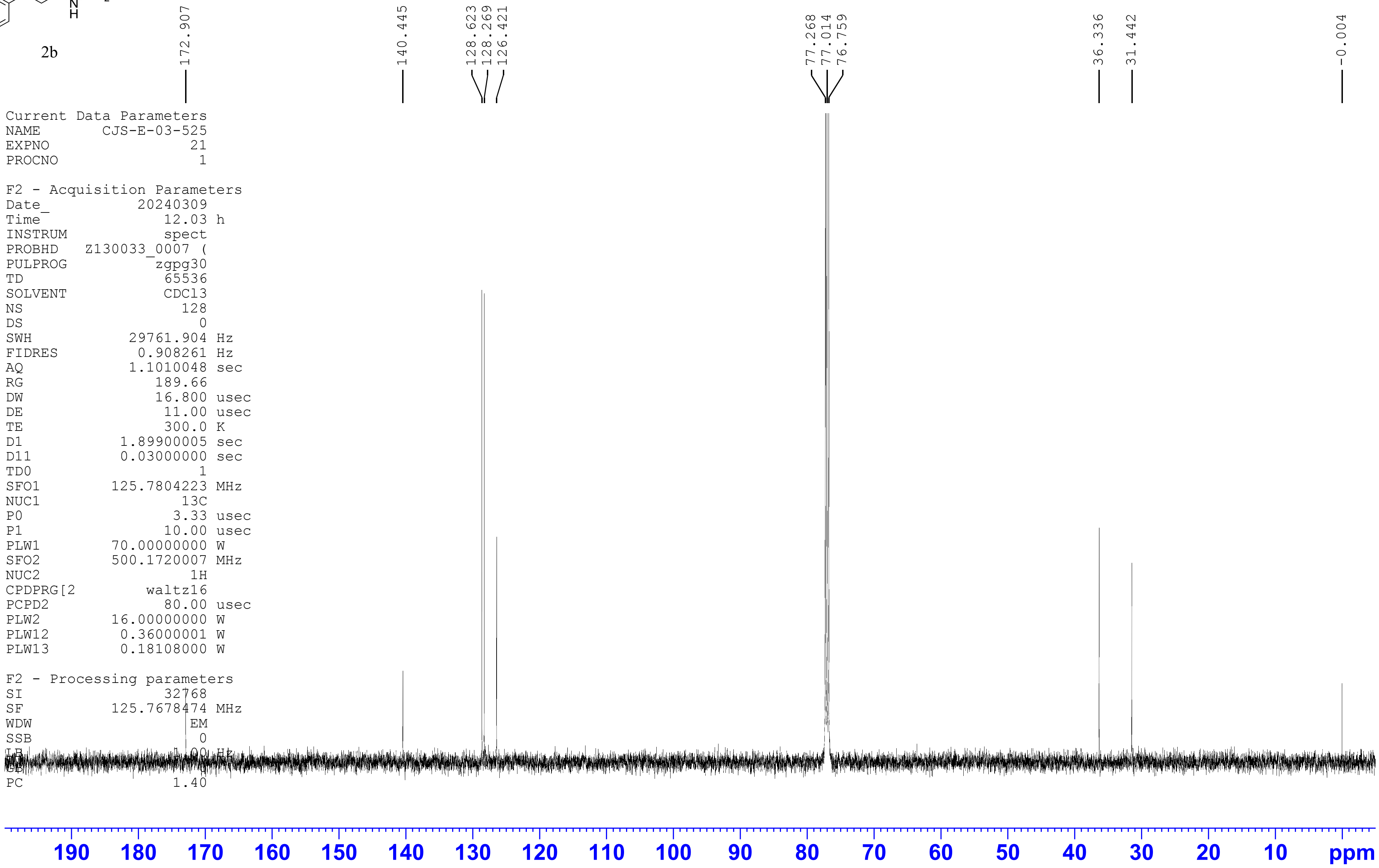

Current Data Parameters
NAME CJS-E-03-525
EXPNO 21
PROCNO 1

F2 - Acquisition Parameters
Date_ 20240309
Time 12.03 h
INSTRUM spect
PROBHD Z130033_0007 (
PULPROG zgpg30
TD 65536
SOLVENT CDCl3
NS 128
DS 0
SWH 29761.904 Hz
FIDRES 0.908261 Hz
AQ 1.1010048 sec
RG 189.66
DW 16.800 usec
DE 11.00 usec
TE 300.0 K
D1 1.89900005 sec
D11 0.03000000 sec
TD0 1
SFO1 125.7804223 MHz
NUC1 13C
P0 3.33 usec
P1 10.00 usec
PLW1 70.00000000 W
SFO2 500.1720007 MHz
NUC2 1H
CPDPRG[2 waltz16
PCPD2 80.00 usec
PLW2 16.00000000 W
PLW12 0.36000001 W
PLW13 0.18108000 W

F2 - Processing parameters
SI 32768
SF 125.7678474 MHz
WDW EM
SSB 0
LB 1.00 Hz
GB 0
PC 1.40

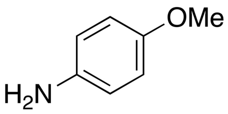

3b

Current Data Parameters
NAME CJS-E-03-nh2ome
EXPNO 10
PROCNO 1

F2 - Acquisition Parameters
Date_ 20240305
Time 10.19 h
INSTRUM spect
PROBHD Z130033_0007 (
PULPROG zg30
TD 65536
SOLVENT CDCl3
NS 1
DS 0
SWH 8012.820 Hz
FIDRES 0.244532 Hz
AQ 4.0894465 sec
RG 31.29
DW 62.400 usec
DE 10.00 usec
TE 300.1 K
D1 1.00000000 sec
TD0 1
SFO1 500.1730010 MHz
NUC1 1H
P0 4.00 usec
P1 12.00 usec
PLW1 16.00000000 W

F2 - Processing parameters
SI 65536
SF 500.1700140 MHz
WDW EM
SSB 0
LB 0.30 Hz
GB 0
PC 1.00

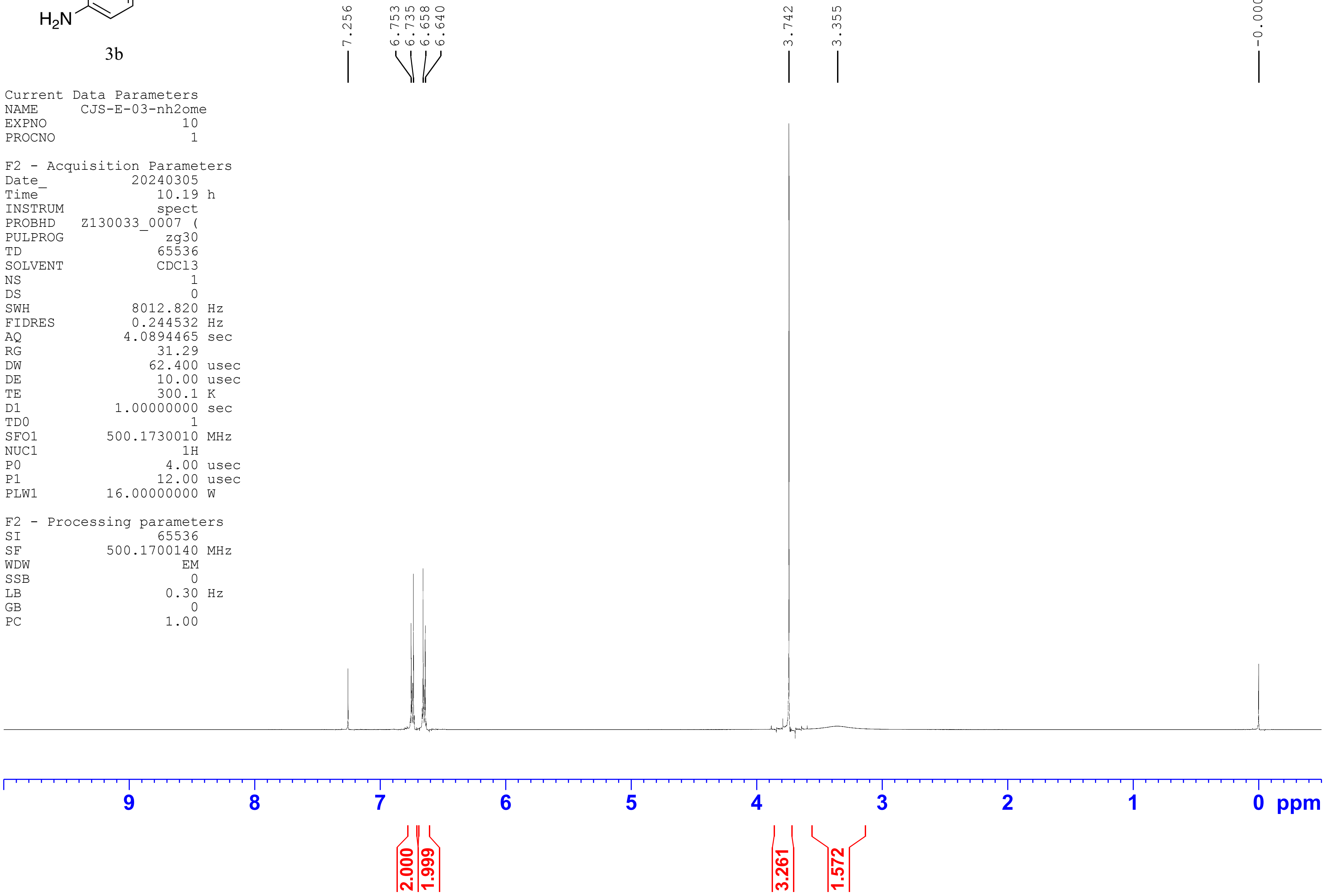

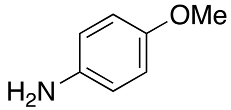

3b

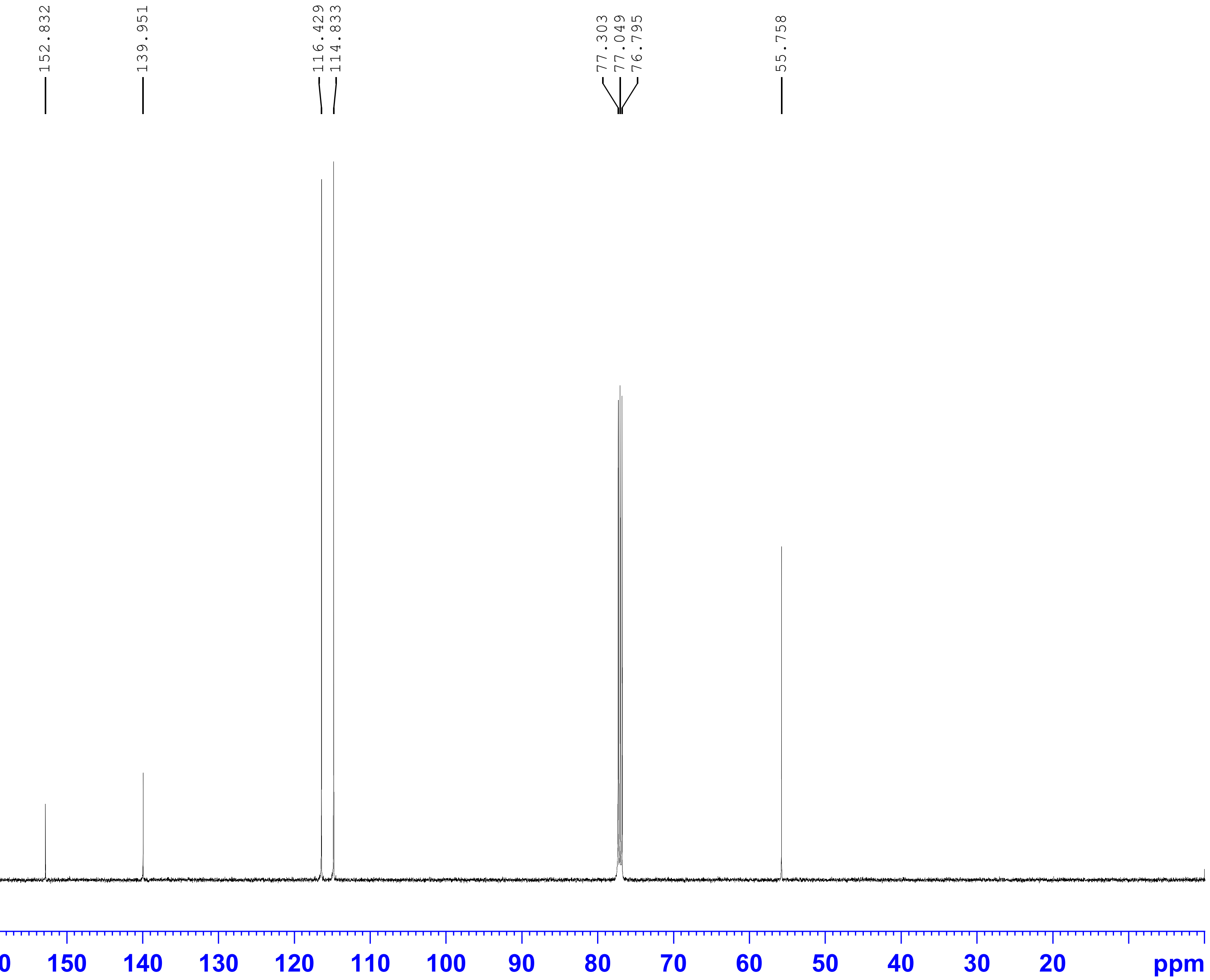


```
Current Data Parameters
NAME     CJS-E-03-panisine
EXPNO                   10
PROCNO                   1

F2 - Acquisition Parameters
Date_             20231203
Time                 19.12 h
INSTRUM              spect
PROBHD   Z130033_0007 (
PULPROG            zgpg30
TD                   65536
SOLVENT              CDCl3
NS                     128
DS                       0
SWH              29761.904 Hz
FIDRES            0.908261 Hz
AQ               1.1010048 sec
RG                  189.66
DW                  16.800 usec
DE                   11.00 usec
TE                   300.0 K
D1              1.89900005 sec
D11             0.03000000 sec
TD0                      1
SFO1           125.7804223 MHz
NUC1                   13C
P0                    3.33 usec
P1                   10.00 usec
PLW1           70.00000000 W
SFO2           500.1720007 MHz
NUC2                    1H
CPDPRG[2           waltz16
PCPD2                80.00 usec
PLW2           16.00000000 W
PLW12           0.36000001 W
PLW13           0.18108000 W

F2 - Processing parameters
SI                   32768
SF             125.7678470 MHz
WDW                     EM
SSB                      0
LB                    1.00 Hz
GB                       0
PC                    1.40
```

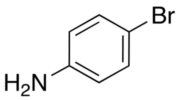

3c

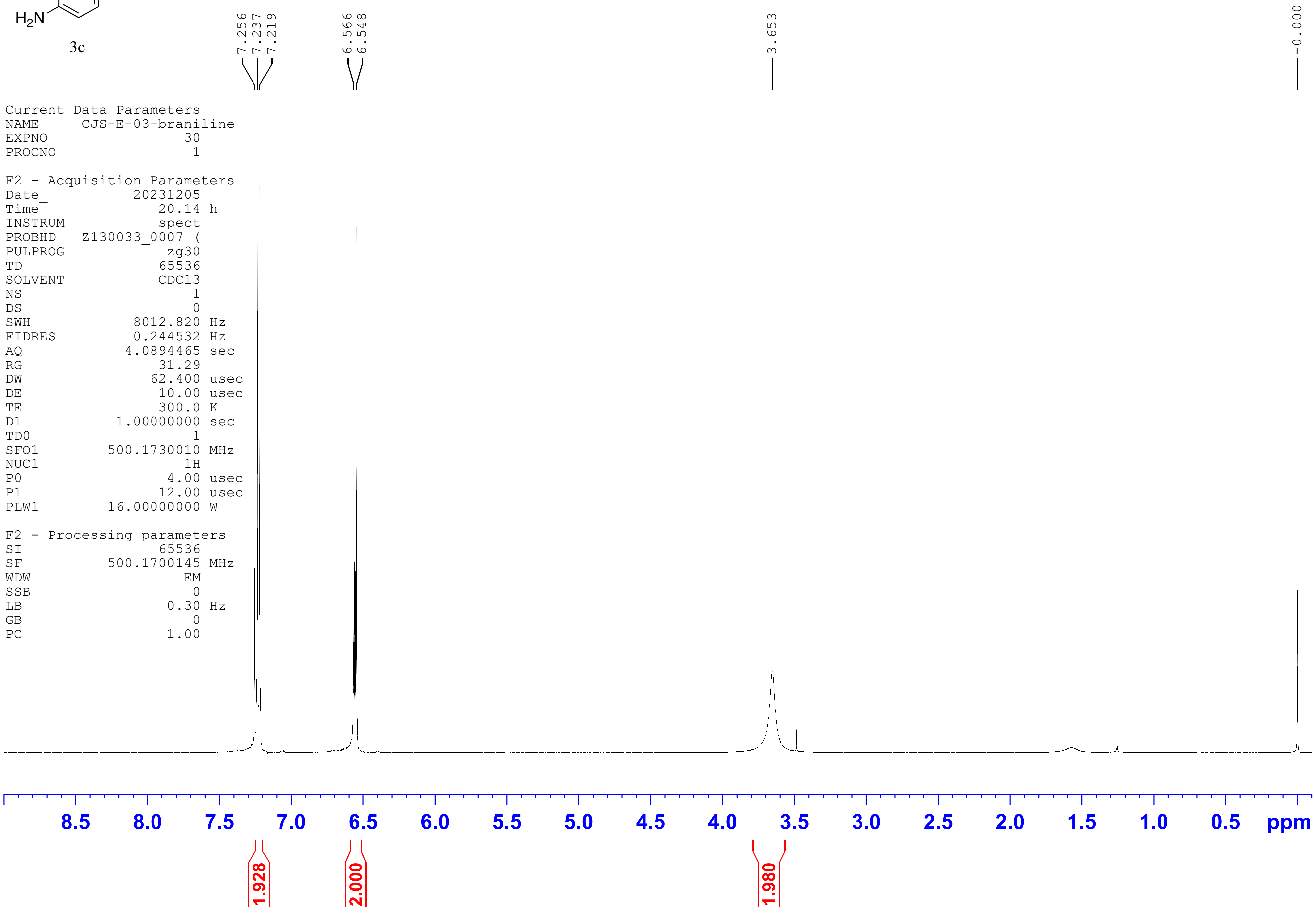
7.256
7.237
7.219
6.566
6.548
3.653
-0.000
Current Data Parameters
NAME CJS-E-03-braniline
EXPNO 30
PROCNO 1
F2 - Acquisition Parameters
Date_ 20231205
Time 20.14 h
INSTRUM spect
PROBHD Z130033_0007 (
PULPROG zg30
TD 65536
SOLVENT CDCl3
NS 1
DS 0
SWH 8012.820 Hz
FIDRES 0.244532 Hz
AQ 4.0894465 sec
RG 31.29
DW 62.400 usec
DE 10.00 usec
TE 300.0 K
D1 1.00000000 sec
TD0 1
SFO1 500.1730010 MHz
NUC1 1H
P0 4.00 usec
P1 12.00 usec
PLW1 16.00000000 W
F2 - Processing parameters
SI 65536
SF 500.1700145 MHz
WDW EM
SSB 0
LB 0.30 Hz
GB 0
PC 1.00
8.5
8.0
7.5
7.0
6.5
6.0
5.5
5.0
4.5
4.0
3.5
3.0
2.5
2.0
1.5
1.0
0.5
ppm
1.928
2.000
1.980

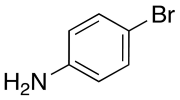

Br
H_2N

3c

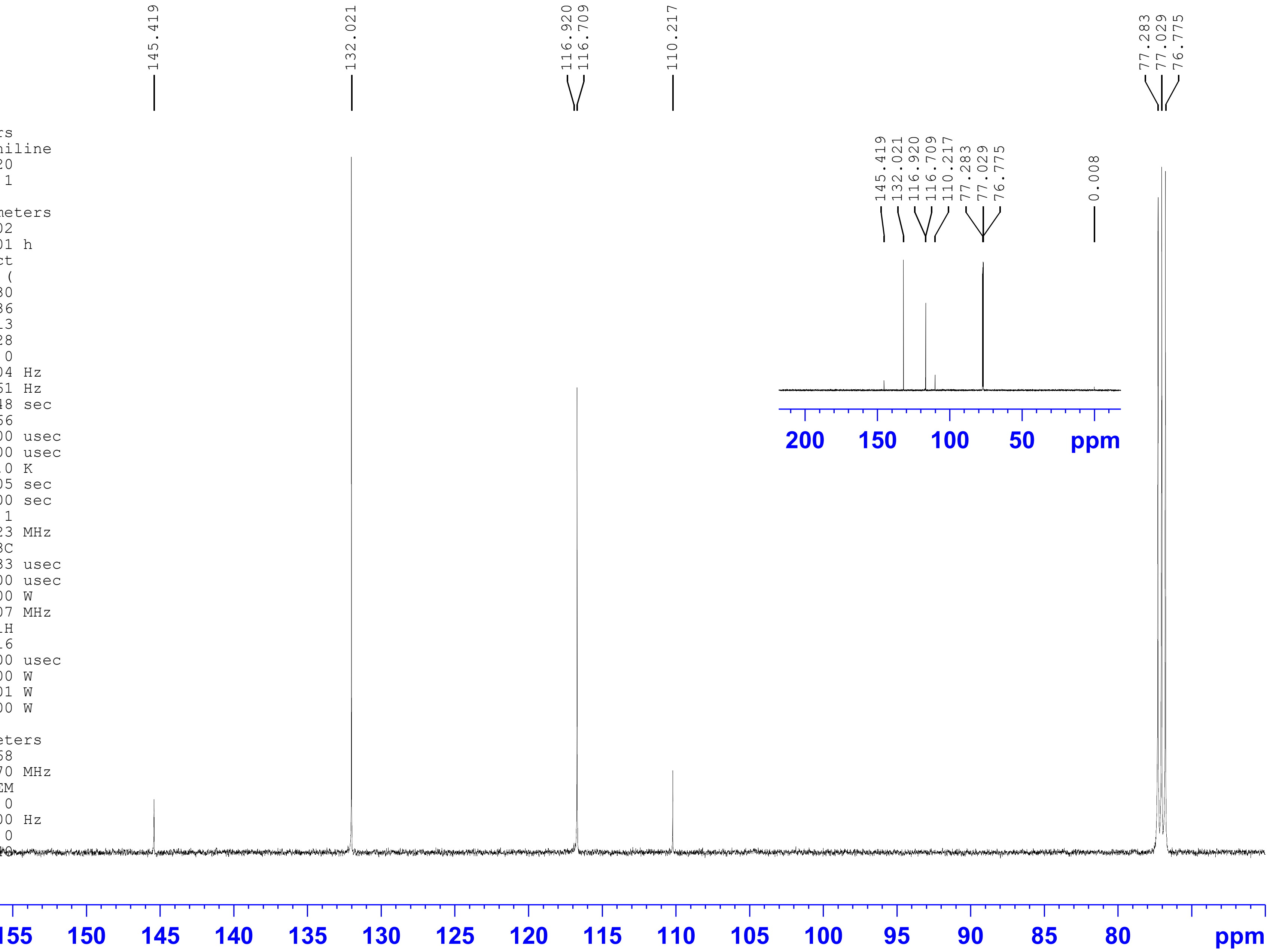

145.419
132.021
116.920
116.709
110.217
77.283
77.029
76.775
0.008
Current Data Parameters
NAME CJS-E-03-braniline
EXPNO 20
PROCNO 1
F2 - Acquisition Parameters
Date_ 20231202
Time 20.01 h
INSTRUM spect
PROBHD Z130033_0007 (
PULPROG zgpg30
TD 65536
SOLVENT CDCl3
NS 128
DS 0
SWH 29761.904 Hz
FIDRES 0.908261 Hz
AQ 1.1010048 sec
RG 189.66
DW 16.800 usec
DE 11.00 usec
TE 300.0 K
D1 1.89900005 sec
D11 0.03000000 sec
TD0 1
SFO1 125.7804223 MHz
NUC1 13C
P0 3.33 usec
P1 10.00 usec
PLW1 70.00000000 W
SFO2 500.1720007 MHz
NUC2 1H
CPDPRG[2 waltz16
PCPD2 80.00 usec
PLW2 16.00000000 W
PLW12 0.36000001 W
PLW13 0.18108000 W
F2 - Processing parameters
SI 32768
SF 125.7678470 MHz
WDW EM
SSB 0
LB 1.00 Hz
GB 0
PC 1.40
200 150 100 50 ppm
165 160 155 150 145 140 135 130 125 120 115 110 105 100 95 90 85 80 ppm

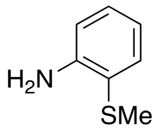

3d

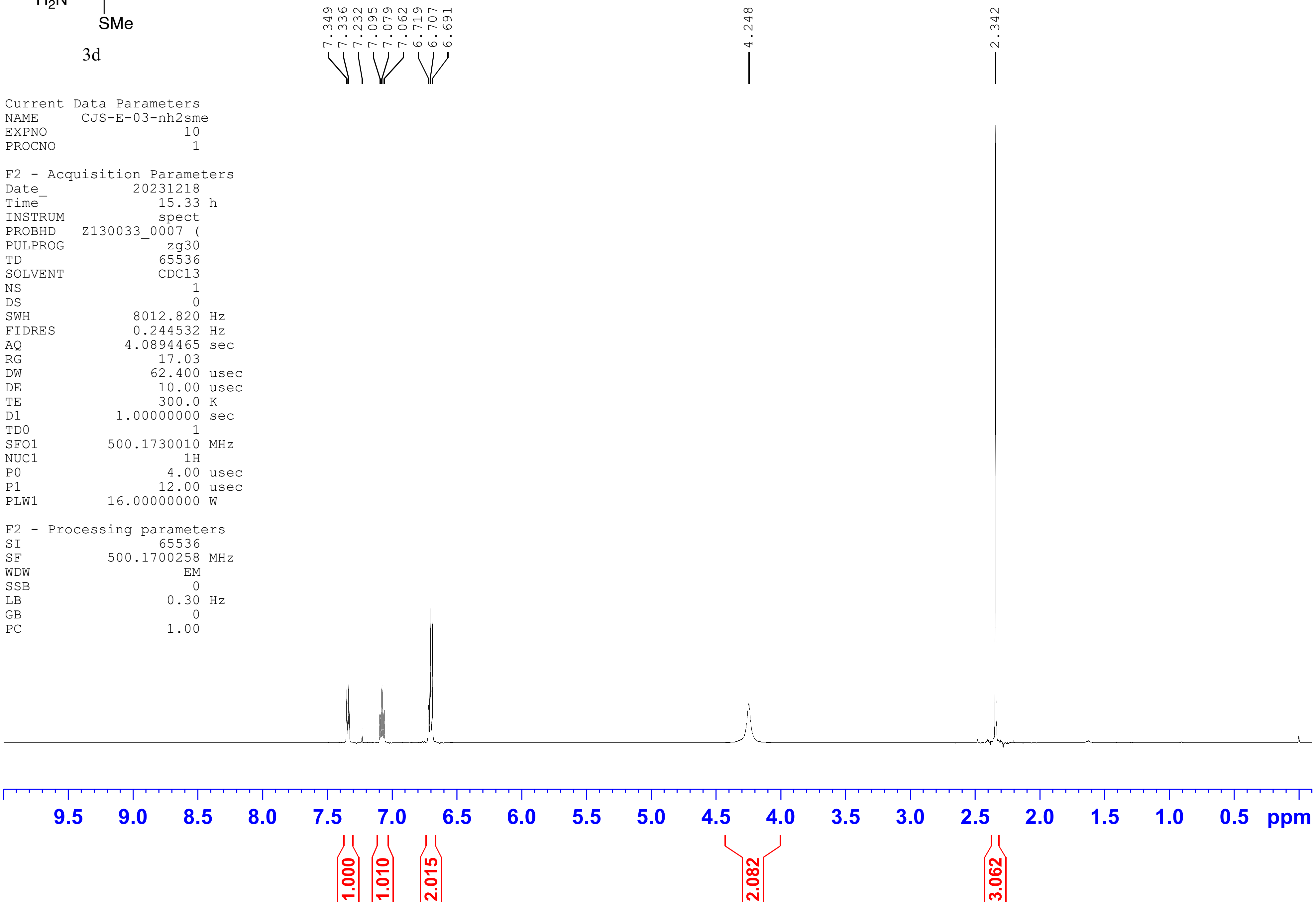

Current Data Parameters
NAME CJS-E-03-nh2sme
EXPNO 10
PROCNO 1

F2 - Acquisition Parameters
Date_ 20231218
Time 15.33 h
INSTRUM spect
PROBHD Z130033_0007 (
PULPROG zg30
TD 65536
SOLVENT CDCl3
NS 1
DS 0
SWH 8012.820 Hz
FIDRES 0.244532 Hz
AQ 4.0894465 sec
RG 17.03
DW 62.400 usec
DE 10.00 usec
TE 300.0 K
D1 1.00000000 sec
TD0 1
SFO1 500.1730010 MHz
NUC1 1H
P0 4.00 usec
P1 12.00 usec
PLW1 16.00000000 W

F2 - Processing parameters
SI 65536
SF 500.1700258 MHz
WDW EM
SSB 0
LB 0.30 Hz
GB 0
PC 1.00

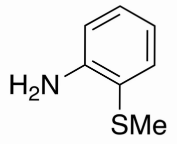

3d

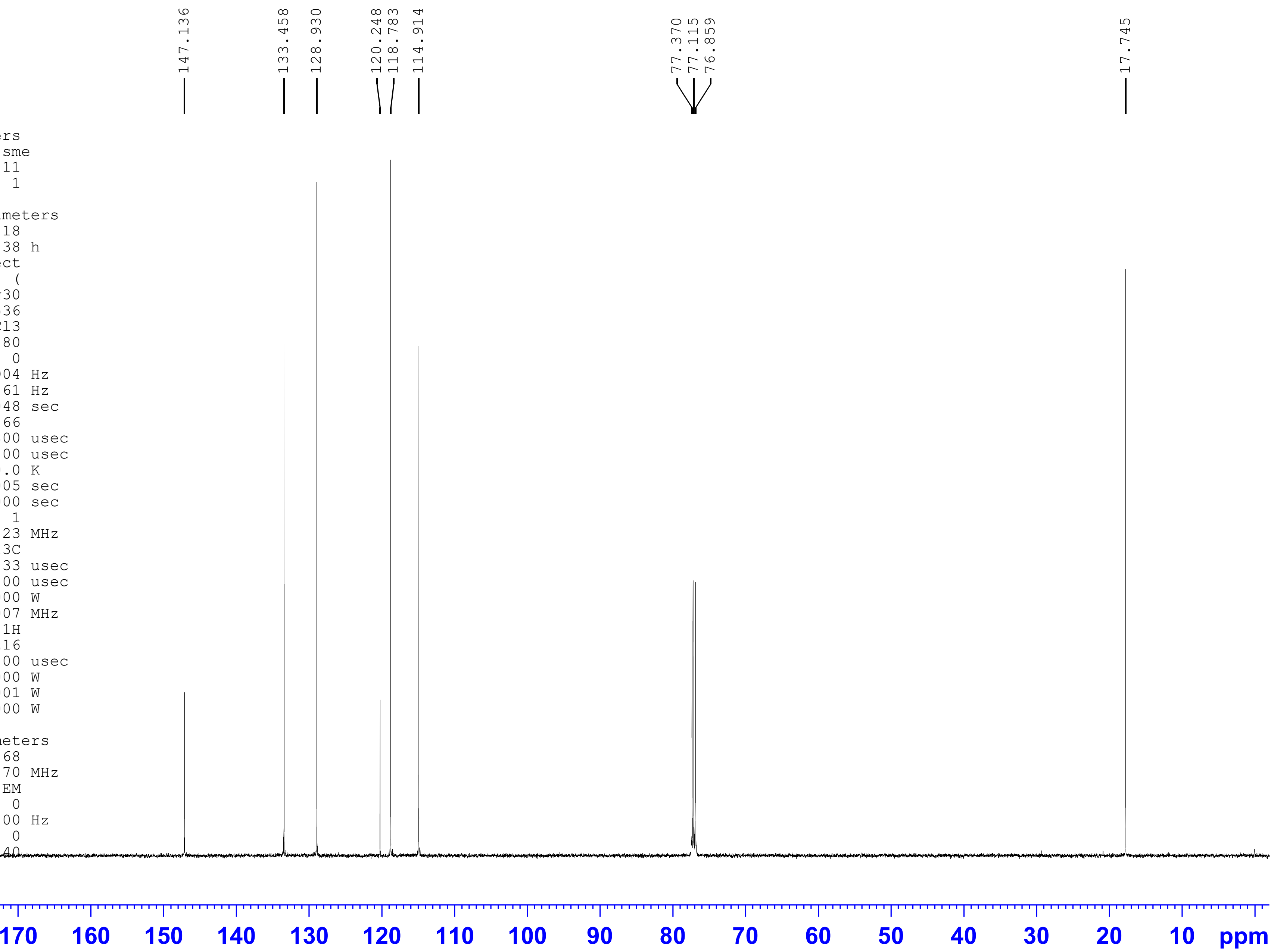


```
Current Data Parameters
NAME     CJS-E-03-nh2sme
EXPNO                  11
PROCNO                  1

F2 - Acquisition Parameters
Date_            20231218
Time                15.38 h
INSTRUM             spect
PROBHD   Z130033_0007 (
PULPROG           zgpg30
TD                 65536
SOLVENT             CDCl3
NS                     80
DS                      0
SWH             29761.904 Hz
FIDRES           0.908261 Hz
AQ              1.1010048 sec
RG                 189.66
DW                 16.800 usec
DE                  11.00 usec
TE                  300.0 K
D1             1.89900005 sec
D11            0.03000000 sec
TD0                     1
SFO1          125.7804223 MHz
NUC1                  13C
P0                   3.33 usec
P1                  10.00 usec
PLW1          70.00000000 W
SFO2          500.1720007 MHz
NUC2                   1H
CPDPRG[2          waltz16
PCPD2               80.00 usec
PLW2          16.00000000 W
PLW12          0.36000001 W
PLW13          0.18108000 W

F2 - Processing parameters
SI                  32768
SF            125.7678470 MHz
WDW                    EM
SSB                     0
LB                   1.00 Hz
GB                      0
PC                   1.40
```

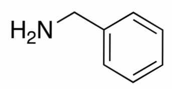

3e

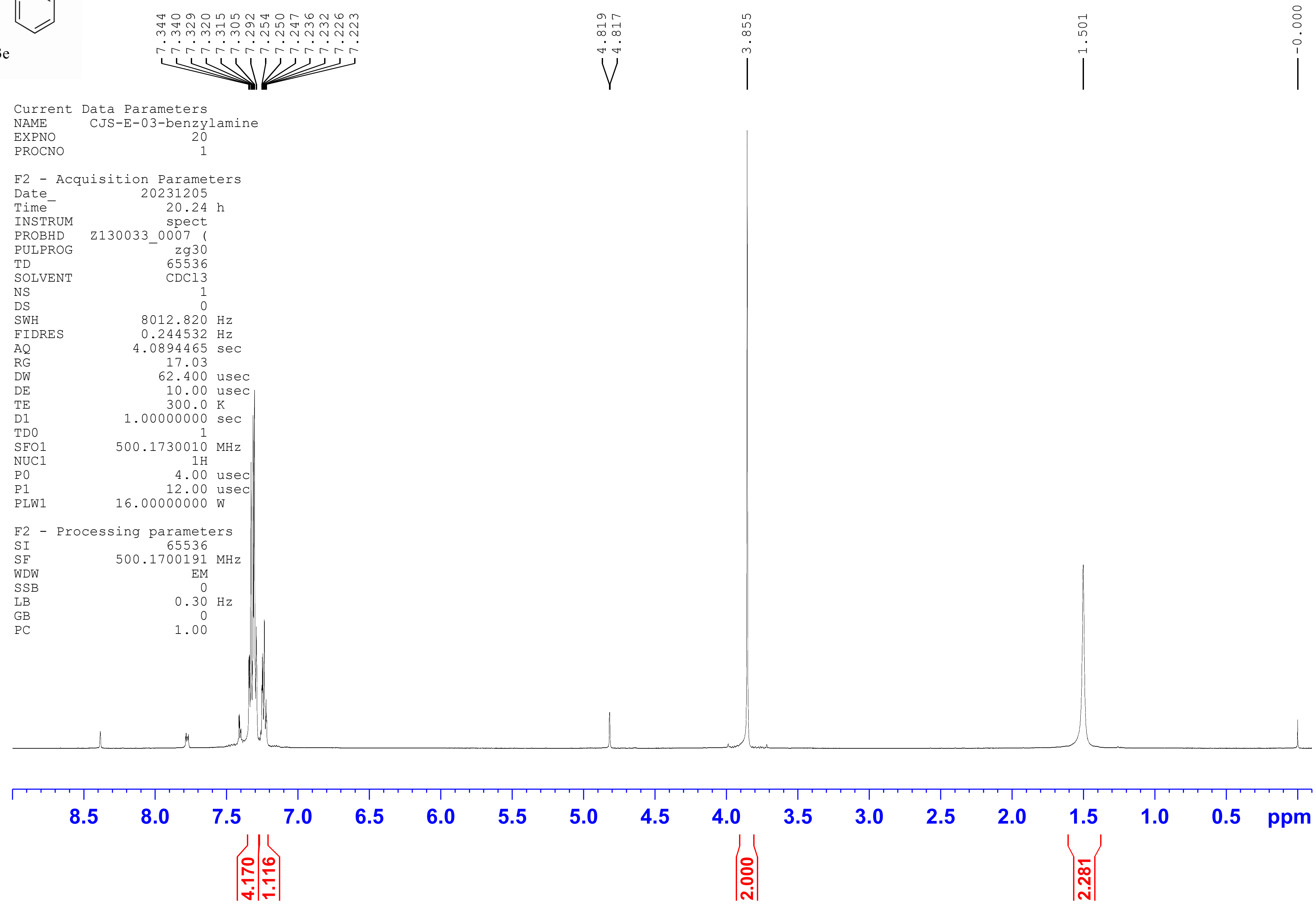

Current Data Parameters
NAME CJS-E-03-benzylamine
EXPNO 20
PROCNO 1

F2 - Acquisition Parameters
Date_ 20231205
Time 20.24 h
INSTRUM spect
PROBHD Z130033_0007 (
PULPROG zg30
TD 65536
SOLVENT CDCl3
NS 1
DS 0
SWH 8012.820 Hz
FIDRES 0.244532 Hz
AQ 4.0894465 sec
RG 17.03
DW 62.400 usec
DE 10.00 usec
TE 300.0 K
D1 1.00000000 sec
TD0 1
SFO1 500.1730010 MHz
NUC1 1H
P0 4.00 usec
P1 12.00 usec
PLW1 16.00000000 W

F2 - Processing parameters
SI 65536
SF 500.1700191 MHz
WDW EM
SSB 0
LB 0.30 Hz
GB 0
PC 1.00

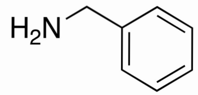

3e

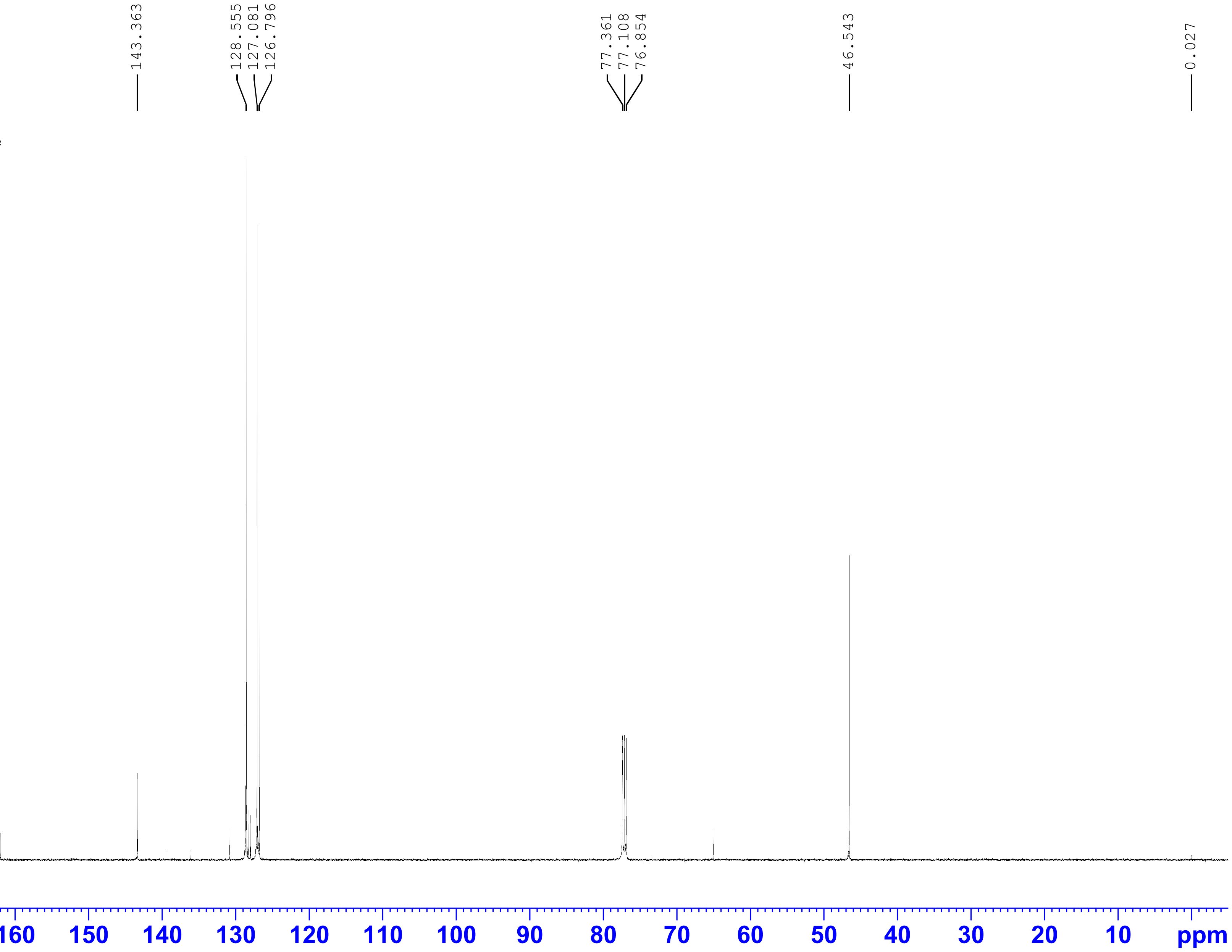


```
Current Data Parameters
NAME     CJS-E-03-benzylamine
EXPNO                  10
PROCNO                  1

F2 - Acquisition Parameters
Date_          20231202
Time              20.23 h
INSTRUM           spect
PROBHD   Z130033_0007 (
PULPROG          zgpg30
TD                65536
SOLVENT           CDCl3
NS                  128
DS                    0
SWH           29761.904 Hz
FIDRES         0.908261 Hz
AQ            1.1010048 sec
RG               189.66
DW               16.800 usec
DE                11.00 usec
TE                300.0 K
D1           1.89900005 sec
D11          0.03000000 sec
TD0                   1
SFO1        125.7804223 MHz
NUC1                13C
P0                 3.33 usec
P1                10.00 usec
PLW1        70.00000000 W
SFO2        500.1720007 MHz
NUC2                 1H
CPDPRG[2        waltz16
PCPD2             80.00 usec
PLW2        16.00000000 W
PLW12        0.36000001 W
PLW13        0.18108000 W

F2 - Processing parameters
SI                32768
SF          125.7678470 MHz
WDW                  EM
SSB                   0
LB                 1.00 Hz
GB                    0
PC                 1.40
```

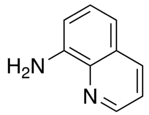

3f

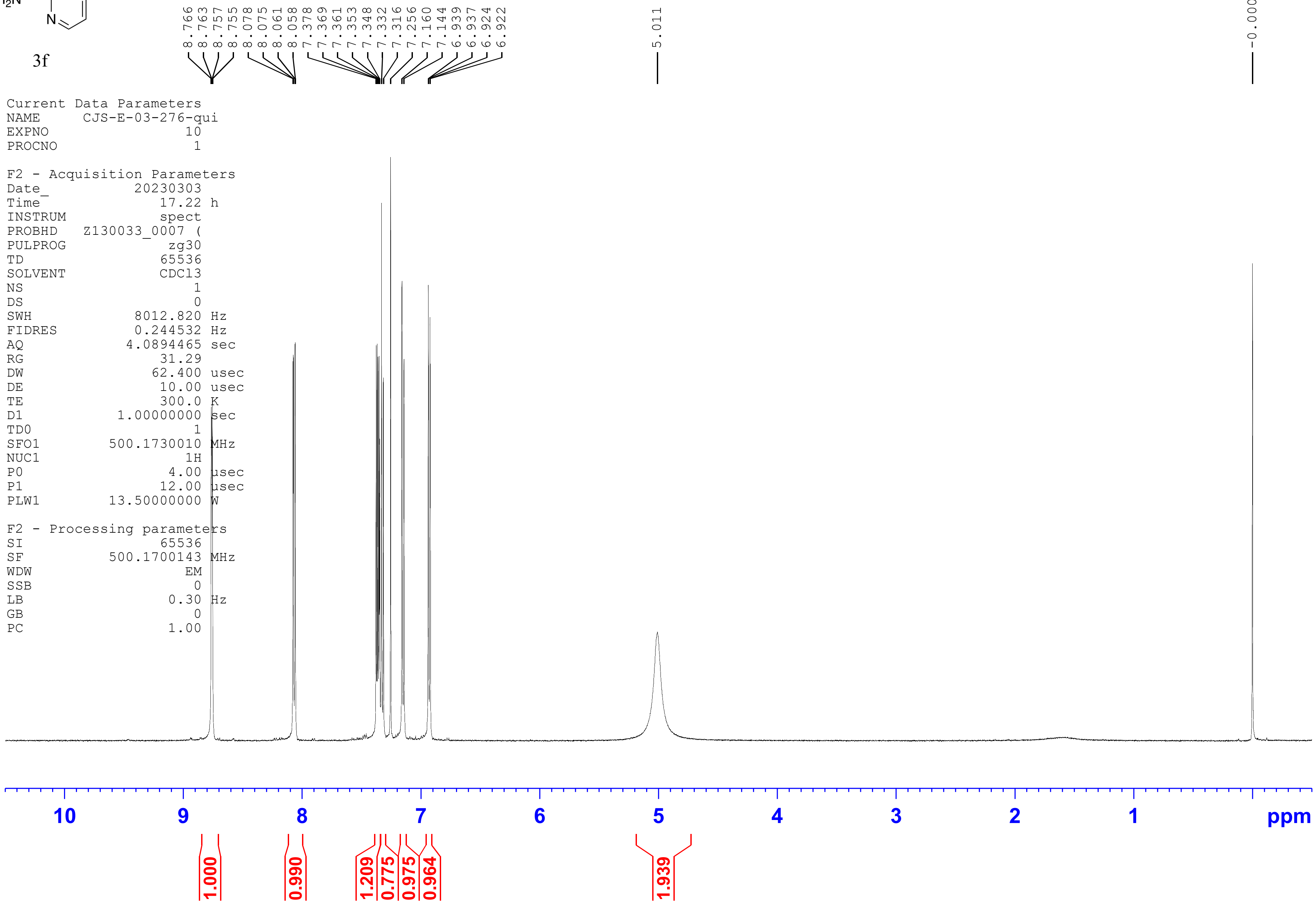

Current Data Parameters
NAME CJS-E-03-276-qui
EXPNO 10
PROCNO 1

F2 - Acquisition Parameters
Date_ 20230303
Time 17.22 h
INSTRUM spect
PROBHD Z130033_0007 (
PULPROG zg30
TD 65536
SOLVENT CDCl3
NS 1
DS 0
SWH 8012.820 Hz
FIDRES 0.244532 Hz
AQ 4.0894465 sec
RG 31.29
DW 62.400 usec
DE 10.00 usec
TE 300.0 K
D1 1.00000000 sec
TD0 1
SFO1 500.1730010 MHz
NUC1 1H
P0 4.00 usec
P1 12.00 usec
PLW1 13.50000000 W

F2 - Processing parameters
SI 65536
SF 500.1700143 MHz
WDW EM
SSB 0
LB 0.30 Hz
GB 0
PC 1.00

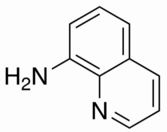

3f

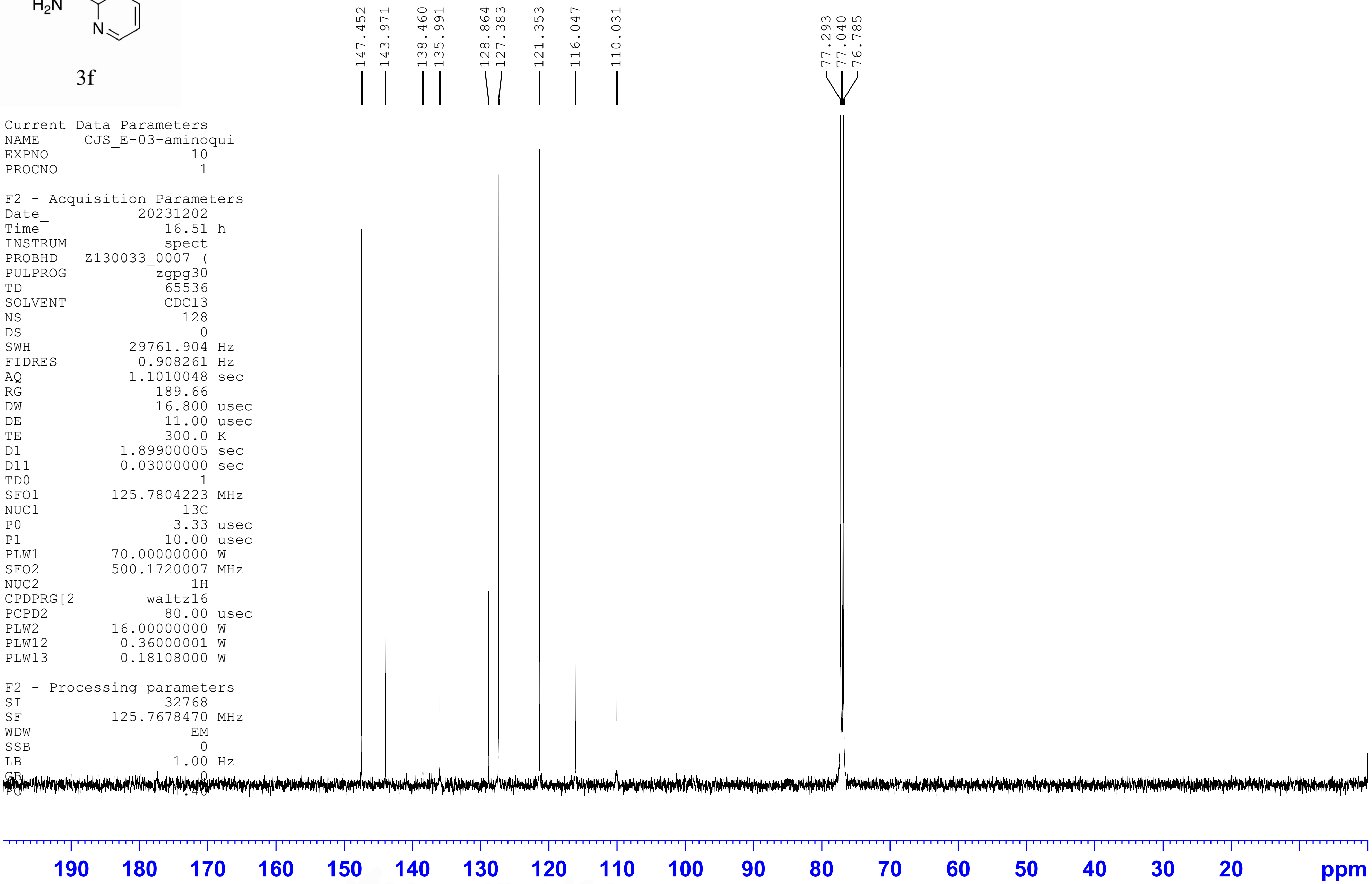
147.452
143.971
138.460
135.991
128.864
127.383
121.353
116.047
110.031
77.293
77.040
76.785
Current Data Parameters
NAME CJS_E-03-aminoqui
EXPNO 10
PROCNO 1
F2 - Acquisition Parameters
Date_ 20231202
Time 16.51 h
INSTRUM spect
PROBHD Z130033_0007 (
PULPROG zgpg30
TD 65536
SOLVENT CDCl3
NS 128
DS 0
SWH 29761.904 Hz
FIDRES 0.908261 Hz
AQ 1.1010048 sec
RG 189.66
DW 16.800 usec
DE 11.00 usec
TE 300.0 K
D1 1.89900005 sec
D11 0.03000000 sec
TD0 1
SFO1 125.7804223 MHz
NUC1 13C
P0 3.33 usec
P1 10.00 usec
PLW1 70.00000000 W
SFO2 500.1720007 MHz
NUC2 1H
CPDPRG[2 waltz16
PCPD2 80.00 usec
PLW2 16.00000000 W
PLW12 0.36000001 W
PLW13 0.18108000 W
F2 - Processing parameters
SI 32768
SF 125.7678470 MHz
WDW EM
SSB 0
LB 1.00 Hz
GB 0
PC 1.40
190
180
170
160
150
140
130
120
110
100
90
80
70
60
50
40
30
20
ppm

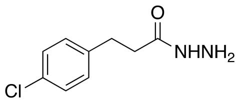

B1

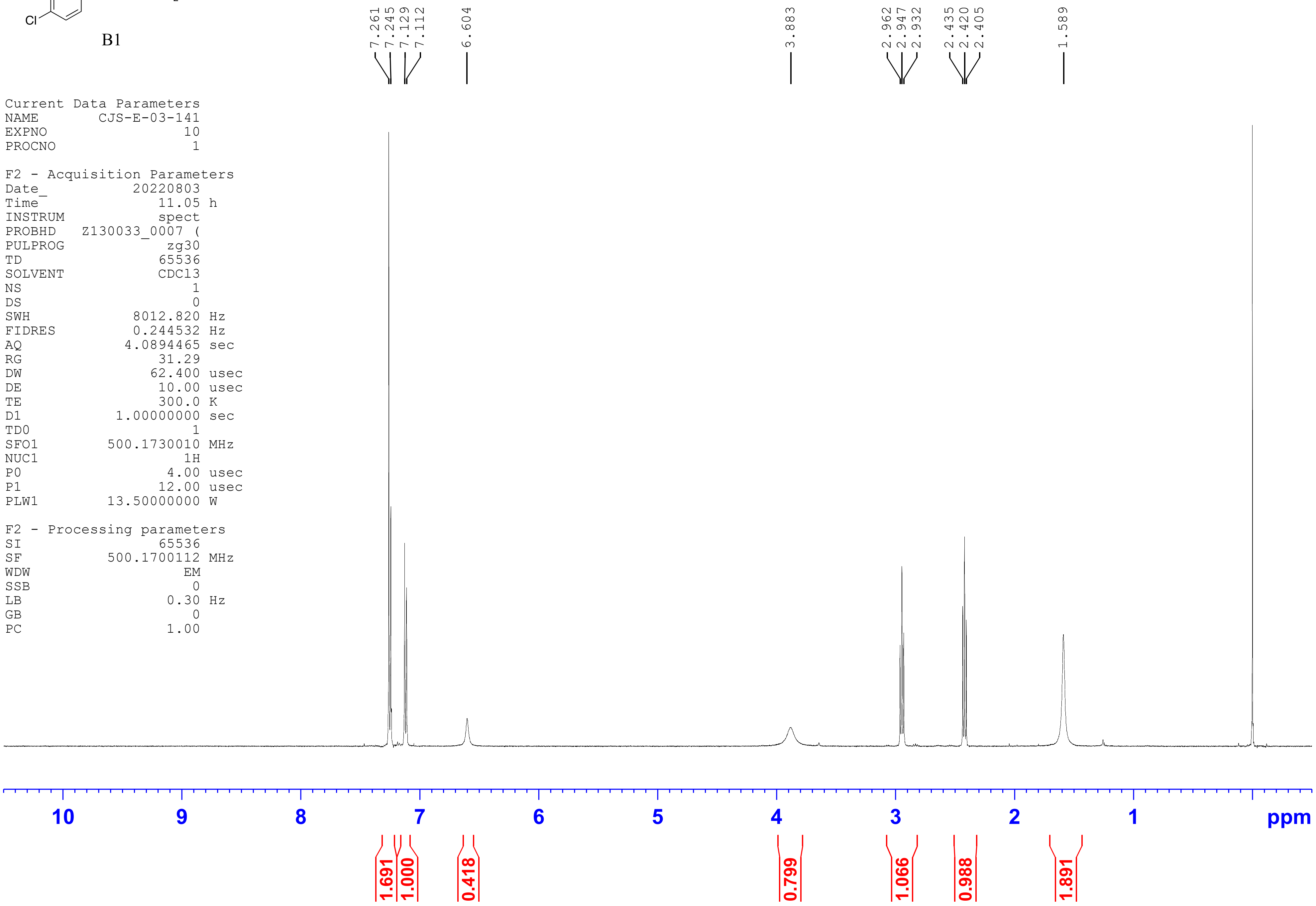

Current Data Parameters
NAME CJS-E-03-141
EXPNO 10
PROCNO 1

F2 - Acquisition Parameters
Date_ 20220803
Time 11.05 h
INSTRUM spect
PROBHD Z130033_0007 (
PULPROG zg30
TD 65536
SOLVENT CDCl3
NS 1
DS 0
SWH 8012.820 Hz
FIDRES 0.244532 Hz
AQ 4.0894465 sec
RG 31.29
DW 62.400 usec
DE 10.00 usec
TE 300.0 K
D1 1.00000000 sec
TD0 1
SFO1 500.1730010 MHz
NUC1 1H
P0 4.00 usec
P1 12.00 usec
PLW1 13.50000000 W

F2 - Processing parameters
SI 65536
SF 500.1700112 MHz
WDW EM
SSB 0
LB 0.30 Hz
GB 0
PC 1.00

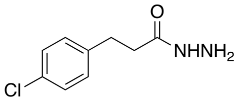

B1

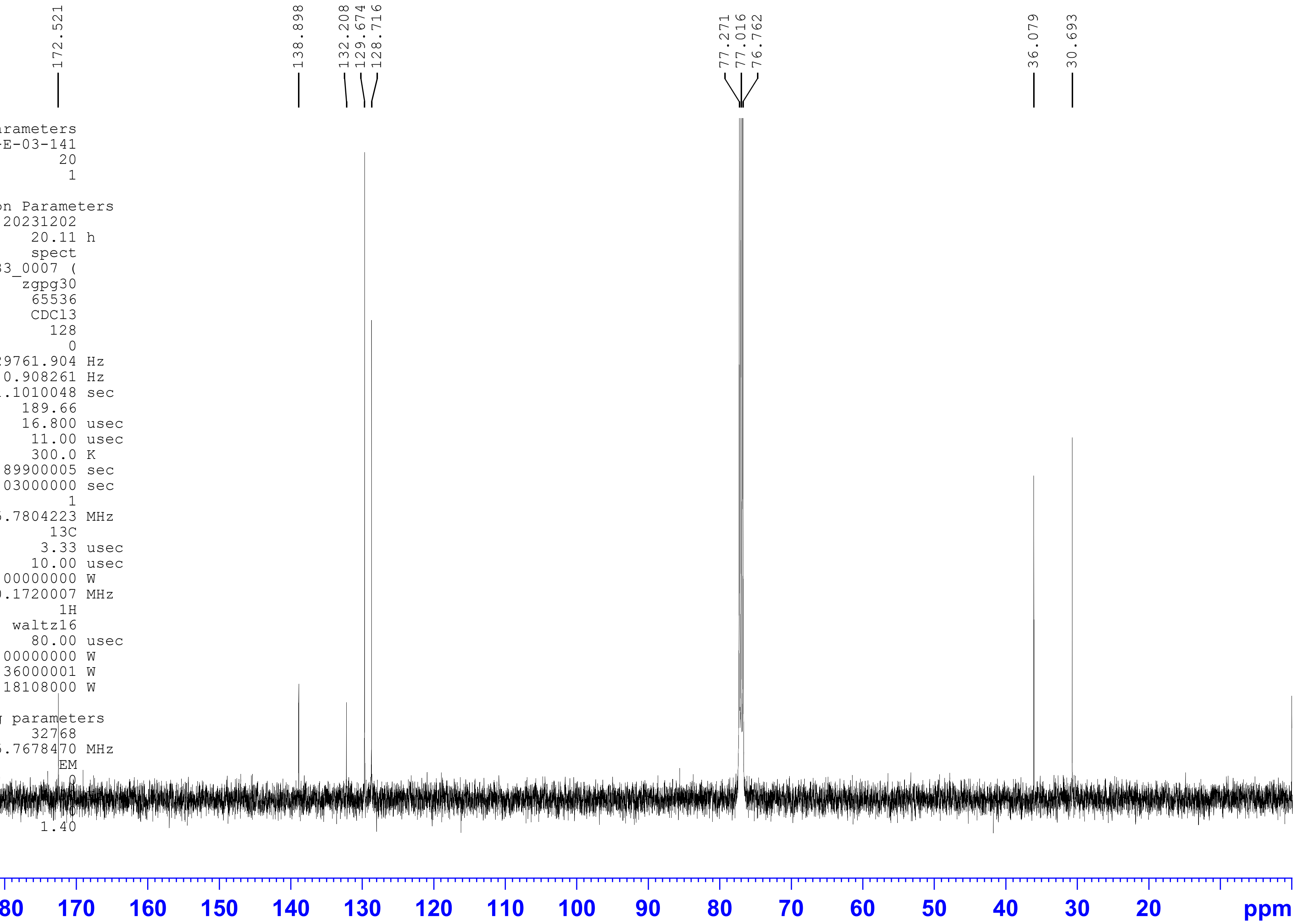


```
Current Data Parameters
NAME        CJS-E-03-141
EXPNO                 20
PROCNO                 1

F2 - Acquisition Parameters
Date_           20231202
Time               20.11 h
INSTRUM            spect
PROBHD    Z130033_0007 (
PULPROG          zgpg30
TD                 65536
SOLVENT            CDCl3
NS                   128
DS                     0
SWH            29761.904 Hz
FIDRES          0.908261 Hz
AQ             1.1010048 sec
RG                189.66
DW                16.800 usec
DE                 11.00 usec
TE                 300.0 K
D1            1.89900005 sec
D11           0.03000000 sec
TD0                    1
SFO1         125.7804223 MHz
NUC1                 13C
P0                  3.33 usec
P1                 10.00 usec
PLW1         70.00000000 W
SFO2         500.1720007 MHz
NUC2                  1H
CPDPRG[2         waltz16
PCPD2              80.00 usec
PLW2         16.00000000 W
PLW12         0.36000001 W
PLW13         0.18108000 W

F2 - Processing parameters
SI                 32768
SF           125.7678470 MHz
WDW                   EM
SSB                    0
LB              [illegible]
GB                     0
PC                  1.40
```

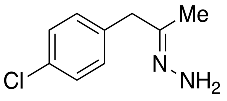

Me
N
NH2
Cl

B4

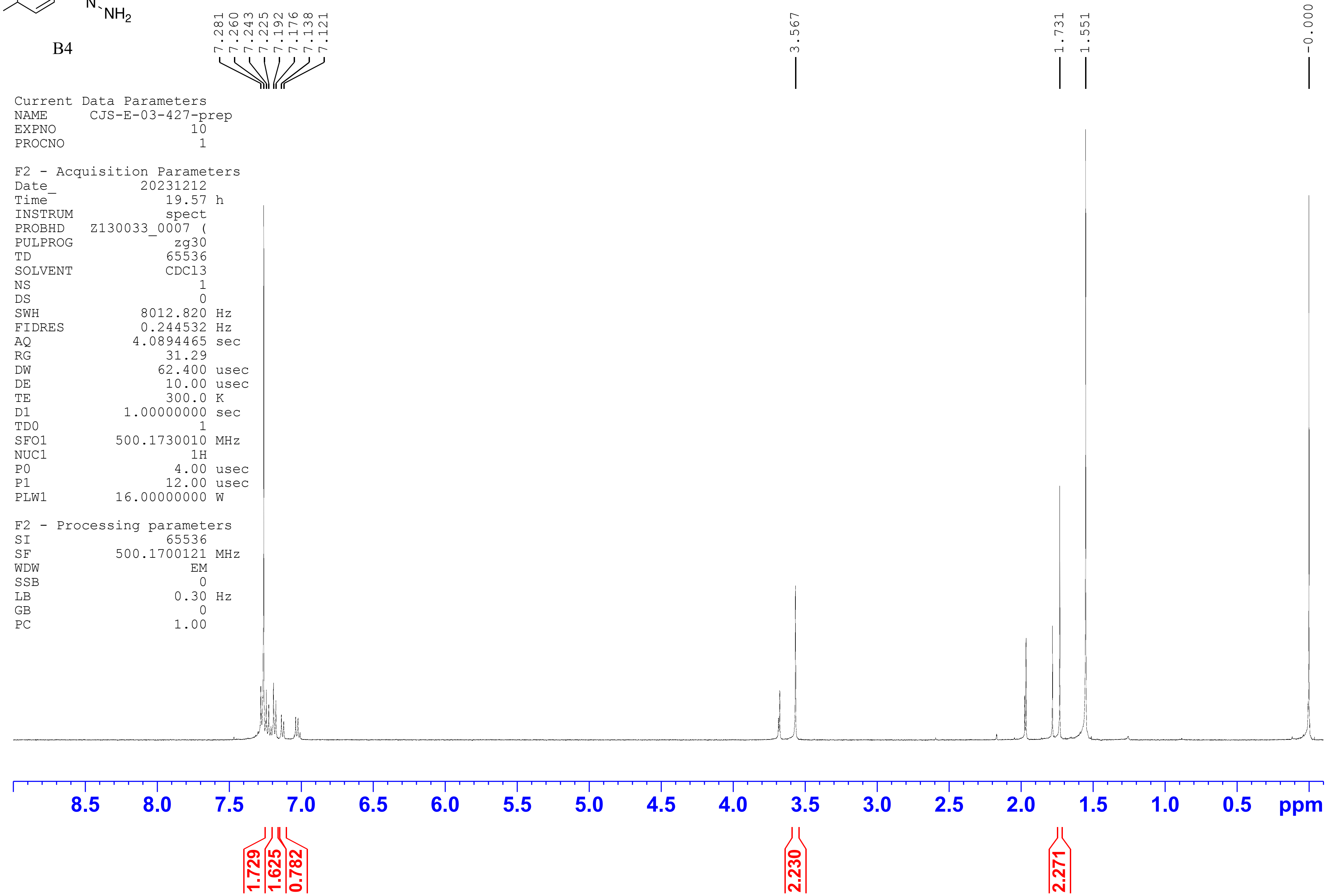

7.281
7.260
7.243
7.225
7.192
7.176
7.138
7.121
3.567
1.731
1.551
-0.000
Current Data Parameters
NAME CJS-E-03-427-prep
EXPNO 10
PROCNO 1
F2 - Acquisition Parameters
Date_ 20231212
Time 19.57 h
INSTRUM spect
PROBHD Z130033_0007 (
PULPROG zg30
TD 65536
SOLVENT CDCl3
NS 1
DS 0
SWH 8012.820 Hz
FIDRES 0.244532 Hz
AQ 4.0894465 sec
RG 31.29
DW 62.400 usec
DE 10.00 usec
TE 300.0 K
D1 1.00000000 sec
TD0 1
SFO1 500.1730010 MHz
NUC1 1H
P0 4.00 usec
P1 12.00 usec
PLW1 16.00000000 W
F2 - Processing parameters
SI 65536
SF 500.1700121 MHz
WDW EM
SSB 0
LB 0.30 Hz
GB 0
PC 1.00
8.5
8.0
7.5
7.0
6.5
6.0
5.5
5.0
4.5
4.0
3.5
3.0
2.5
2.0
1.5
1.0
0.5
ppm
1.729
1.625
0.782
2.230
2.271

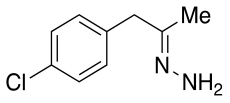

B4

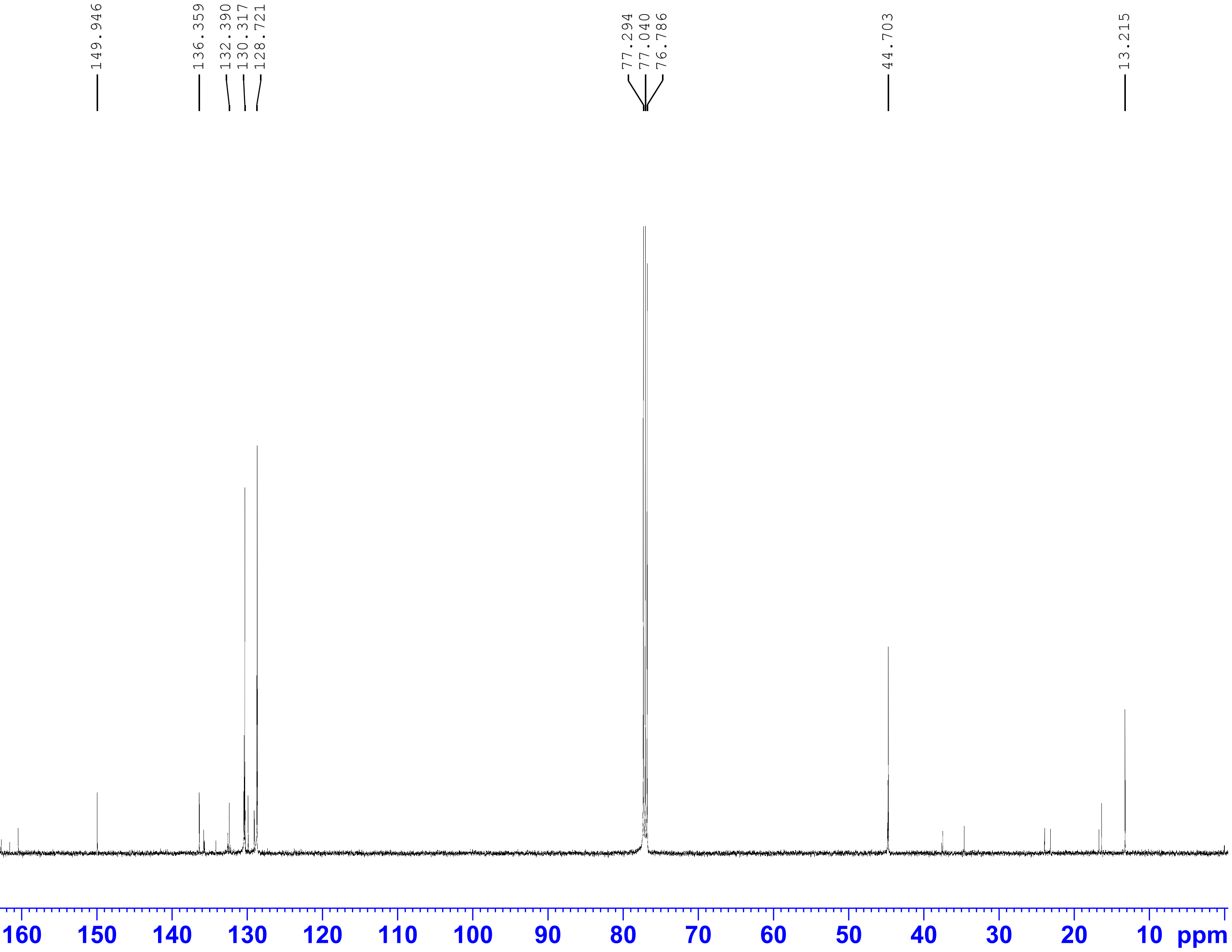

Current Data Parameters		
NAME	CJS-E-03-427	
EXPNO	20	
PROCNO	1	
F2 - Acquisition Parameters		
Date_	20231202	
Time	17.16	h
INSTRUM	spect	
PROBHD	Z130033_0007 (	
PULPROG	zgpg30	
TD	65536	
SOLVENT	CDCl3	
NS	128	
DS	0	
SWH	29761.904	Hz
FIDRES	0.908261	Hz
AQ	1.1010048	sec
RG	189.66	
DW	16.800	usec
DE	11.00	usec
TE	300.0	K
D1	1.89900005	sec
D11	0.03000000	sec
TD0	1	
SFO1	125.7804223	MHz
NUC1	13C	
P0	3.33	usec
P1	10.00	usec
PLW1	70.00000000	W
SFO2	500.1720007	MHz
NUC2	1H	
CPDPRG[2	waltz16	
PCPD2	80.00	usec
PLW2	16.00000000	W
PLW12	0.36000001	W
PLW13	0.18108000	W
F2 - Processing parameters		
SI	32768	
SF	125.7678470	MHz
WDW	EM	
SSB	0	
LB	1.00	Hz
GB	0	
PC	1.40	

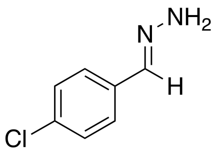

B5

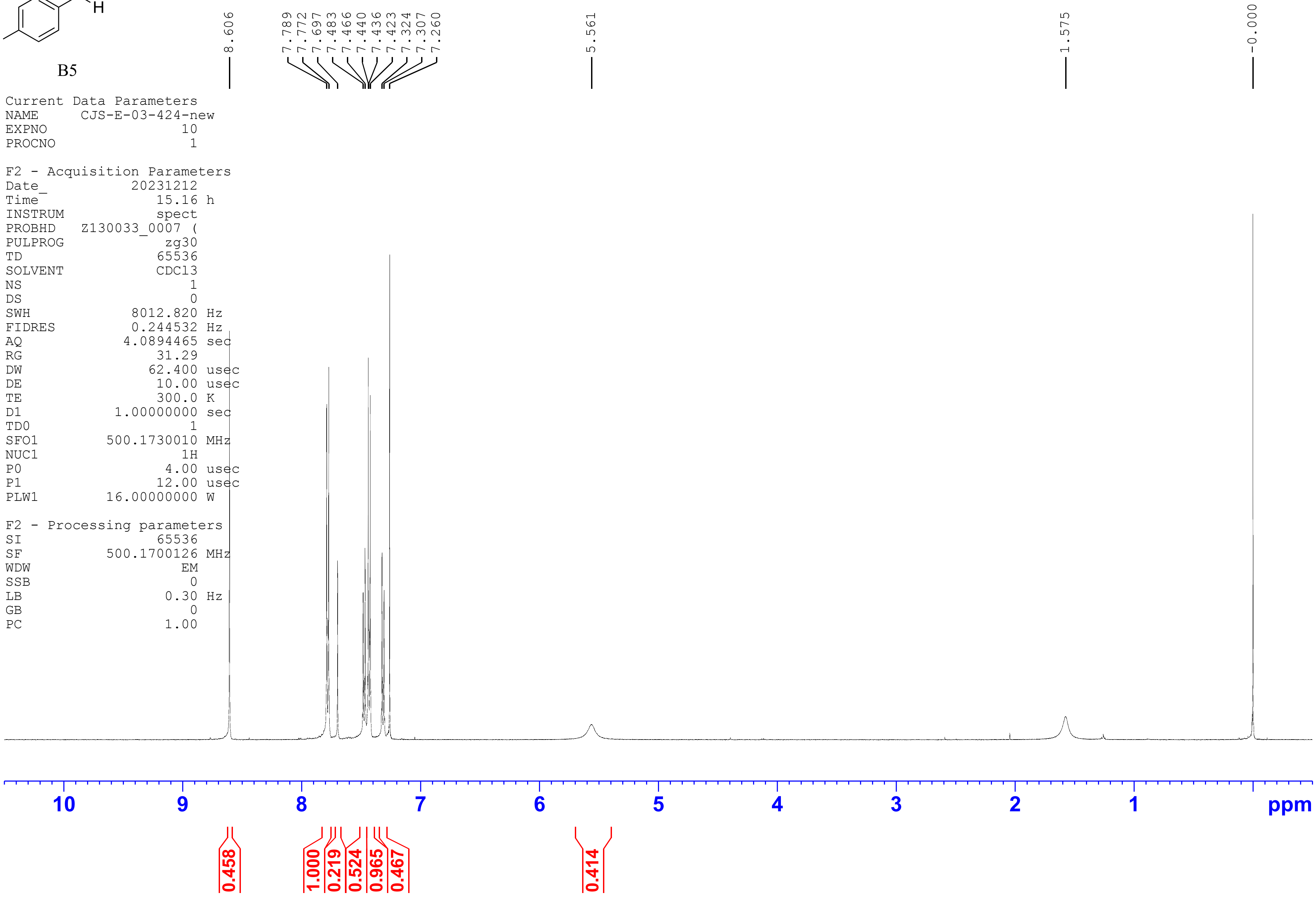
8.606
7.789
7.772
7.697
7.483
7.466
7.440
7.436
7.423
7.324
7.307
7.260
5.561
1.575
-0.000
Current Data Parameters
NAME CJS-E-03-424-new
EXPNO 10
PROCNO 1
F2 - Acquisition Parameters
Date_ 20231212
Time 15.16 h
INSTRUM spect
PROBHD Z130033_0007 (
PULPROG zg30
TD 65536
SOLVENT CDCl3
NS 1
DS 0
SWH 8012.820 Hz
FIDRES 0.244532 Hz
AQ 4.0894465 sec
RG 31.29
DW 62.400 usec
DE 10.00 usec
TE 300.0 K
D1 1.00000000 sec
TD0 1
SFO1 500.1730010 MHz
NUC1 1H
P0 4.00 usec
P1 12.00 usec
PLW1 16.00000000 W
F2 - Processing parameters
SI 65536
SF 500.1700126 MHz
WDW EM
SSB 0
LB 0.30 Hz
GB 0
PC 1.00
10
9
8
7
6
5
4
3
2
1
ppm
0.458
1.000
0.219
0.524
0.965
0.467
0.414

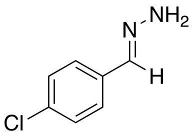

B5

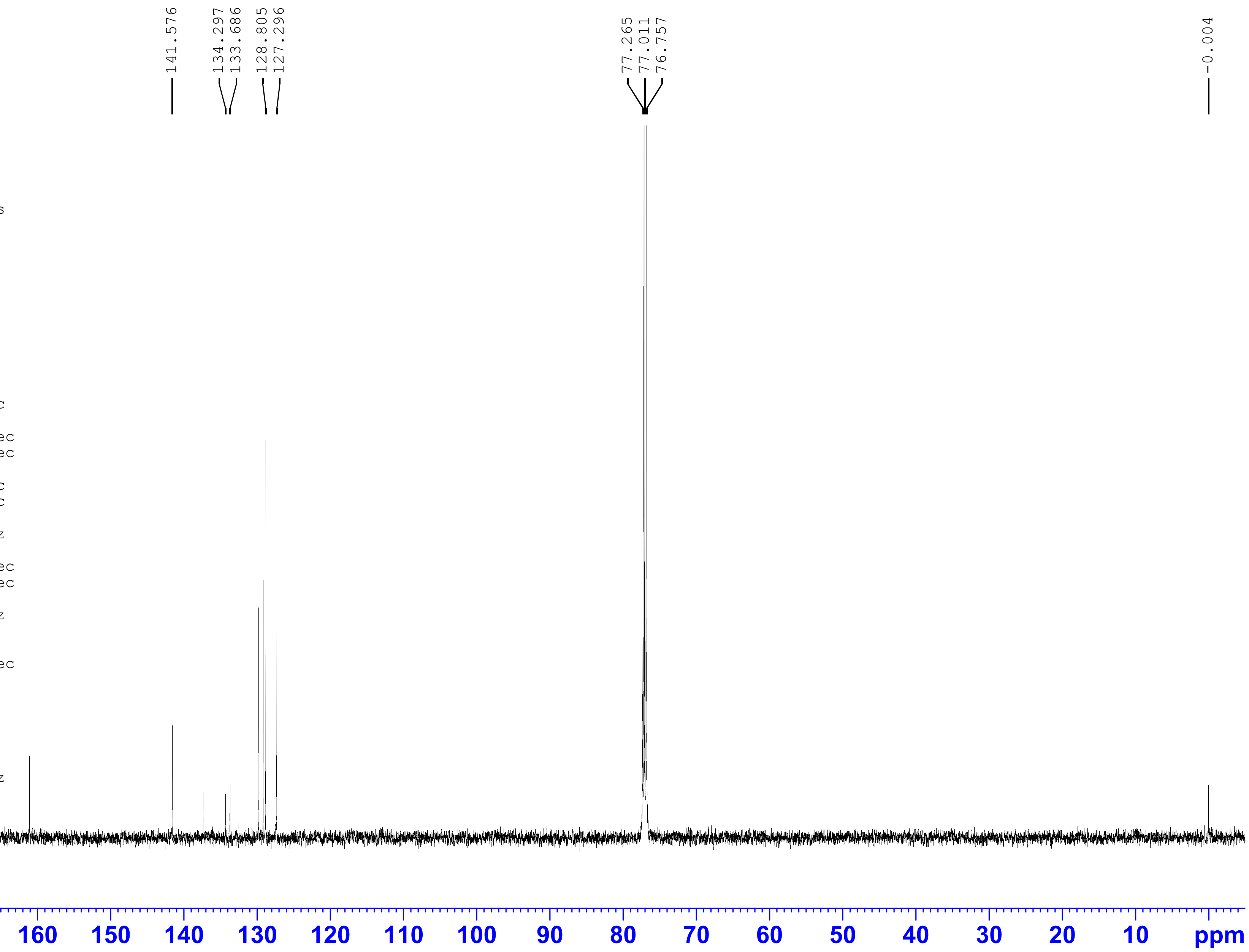

Current Data Parameters
NAME CJS-E-03-424
EXPNO 10
PROCNO 1

F2 - Acquisition Parameters
Date_ 20231203
Time 19.33 h
INSTRUM spect
PROBHD Z130033_0007 (
PULPROG zgpg30
TD 65536
SOLVENT CDCl3
NS 128
DS 0
SWH 29761.904 Hz
FIDRES 0.908261 Hz
AQ 1.1010048 sec
RG 189.66
DW 16.800 usec
DE 11.00 usec
TE 300.0 K
D1 1.89900005 sec
D11 0.03000000 sec
TD0 1
SFO1 125.7804223 MHz
NUC1 13C
P0 3.33 usec
P1 10.00 usec
PLW1 70.00000000 W
SFO2 500.1720007 MHz
NUC2 1H
CPDPRG[2 waltz16
PCPD2 80.00 usec
PLW2 16.00000000 W
PLW12 0.36000001 W
PLW13 0.18108000 W

F2 - Processing parameters
SI 32768
SF 125.7678473 MHz
WDW EM
SSB 0
LB 1.00 Hz
GB 0
PC 1.40

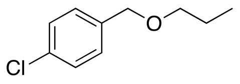

B10

Current Data Parameters
NAME CJS-E-03-414-1
EXPNO 10
PROCNO 1

F2 - Acquisition Parameters
Date_ 20231122
Time 11.51 h
INSTRUM spect
PROBHD Z130033_0007 (
PULPROG zg30
TD 65536
SOLVENT CDCl3
NS 1
DS 0
SWH 8012.820 Hz
FIDRES 0.244532 Hz
AQ 4.0894465 sec
RG 31.29
DW 62.400 usec
DE 10.00 usec
TE 300.0 K
D1 1.00000000 sec
TD0 1
SFO1 500.1730010 MHz
NUC1 1H
P0 4.00 usec
P1 12.00 usec
PLW1 16.00000000 W

F2 - Processing parameters
SI 65536
SF 500.1700138 MHz
WDW EM
SSB 0
LB 0.30 Hz
GB 0
PC 1.00

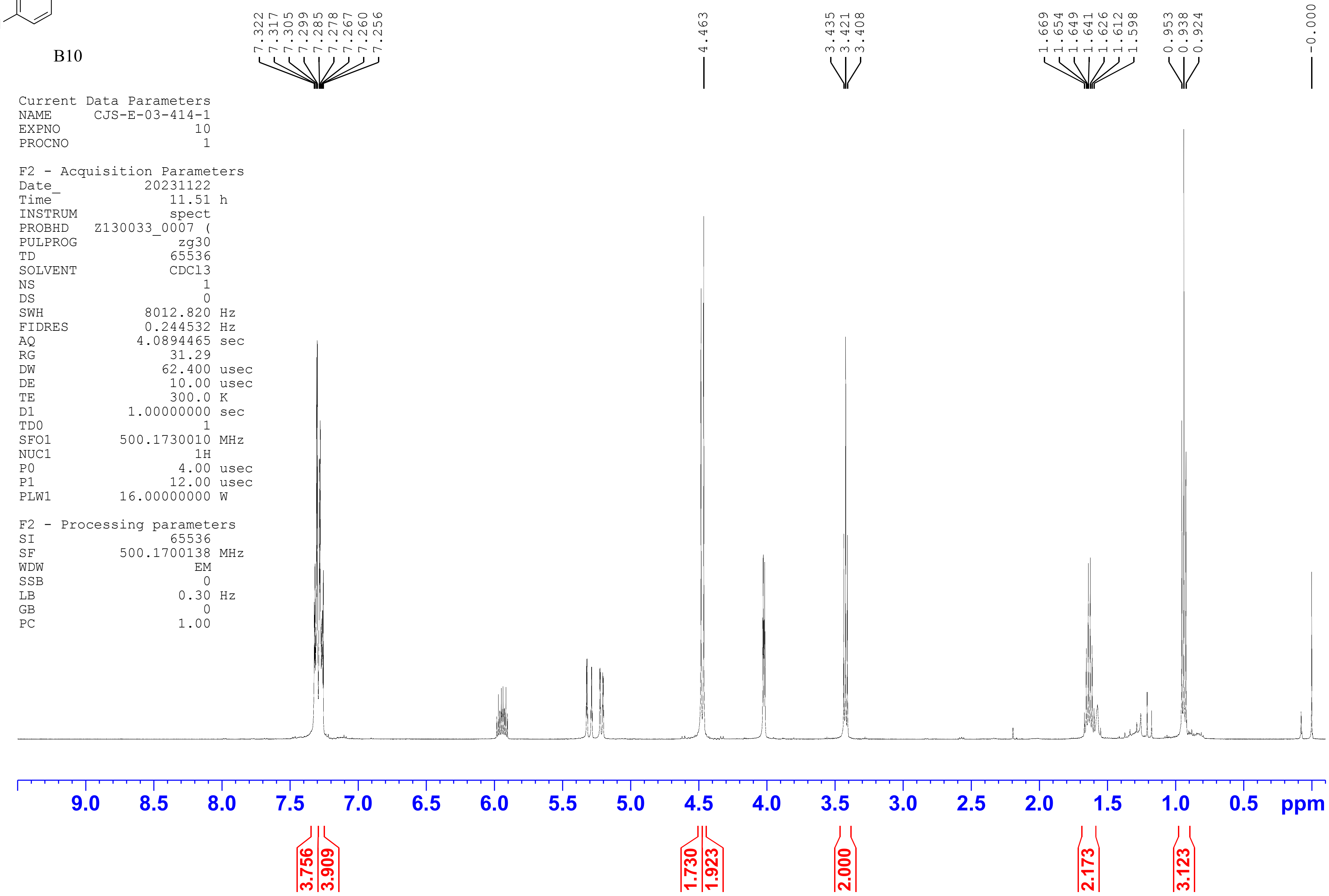

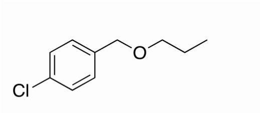

B10

Current Data Parameters
NAME CJS-E-03-111-1spot
EXPNO 30
PROCNO 1

F2 - Acquisition Parameters
Date_ 20220617
Time 10.21 h
INSTRUM spect
PROBHD Z130033_0007 (
PULPROG zgpg30
TD 65536
SOLVENT CDCl3
NS 256
DS 0
SWH 29761.904 Hz
FIDRES 0.908261 Hz
AQ 1.1010048 sec
RG 189.66
DW 16.800 usec
DE 11.00 usec
TE 300.0 K
D1 1.89900005 sec
D11 0.03000000 sec
TD0 1
SFO1 125.7804233 MHz
NUC1 13C
P0 3.33 usec
P1 10.00 usec
PLW1 65.00000000 W
SFO2 500.1720007 MHz
NUC2 1H
CPDPRG[2 waltz16
PCPD2 80.00 usec
PLW2 13.50000000 W
PLW12 0.30375001 W
PLW13 0.15278000 W

F2 - Processing parameters
SI 32768
SF 125.7678465 MHz
WDW EM
SSB 0
LB 1.00 Hz
GB 0
PC 1.40

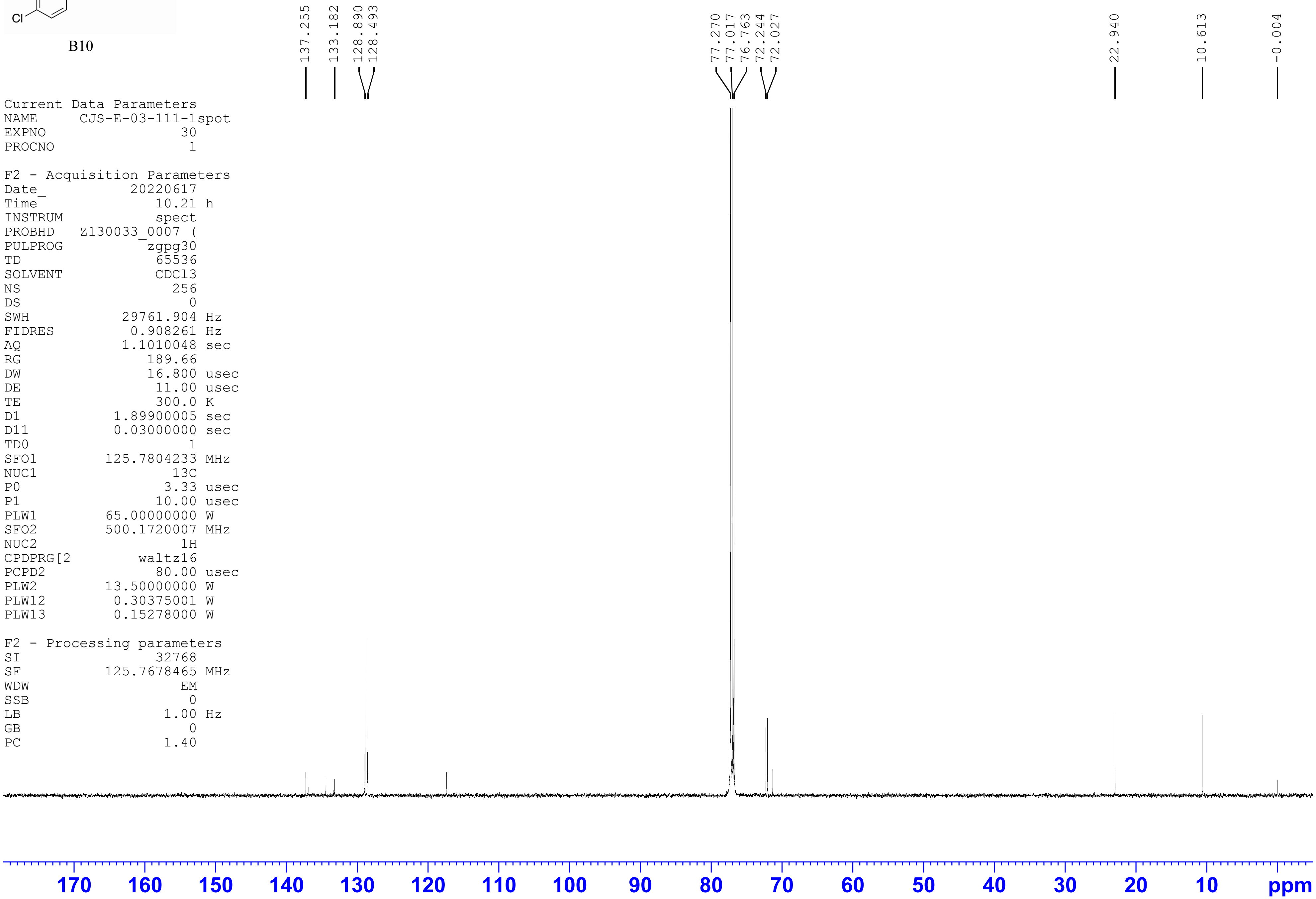

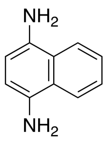

B13

Current Data Parameters
NAME CJS-E-03-422-16tube
EXPNO 10
PROCNO 1

F2 - Acquisition Parameters
Date_ 20231219
Time 12.12 h
INSTRUM spect
PROBHD Z130033_0007 (
PULPROG zg30
TD 65536
SOLVENT Acetone
NS 1
DS 0
SWH 8012.820 Hz
FIDRES 0.244532 Hz
AQ 4.0894465 sec
RG 31.29
DW 62.400 usec
DE 10.00 usec
TE 300.0 K
D1 1.00000000 sec
TD0 1
SFO1 500.1730010 MHz
NUC1 1H
P0 4.00 usec
P1 12.00 usec
PLW1 16.00000000 W

F2 - Processing parameters
SI 65536
SF 500.1700754 MHz
WDW EM
SSB 0
LB 0.30 Hz
GB 0
PC 1.00

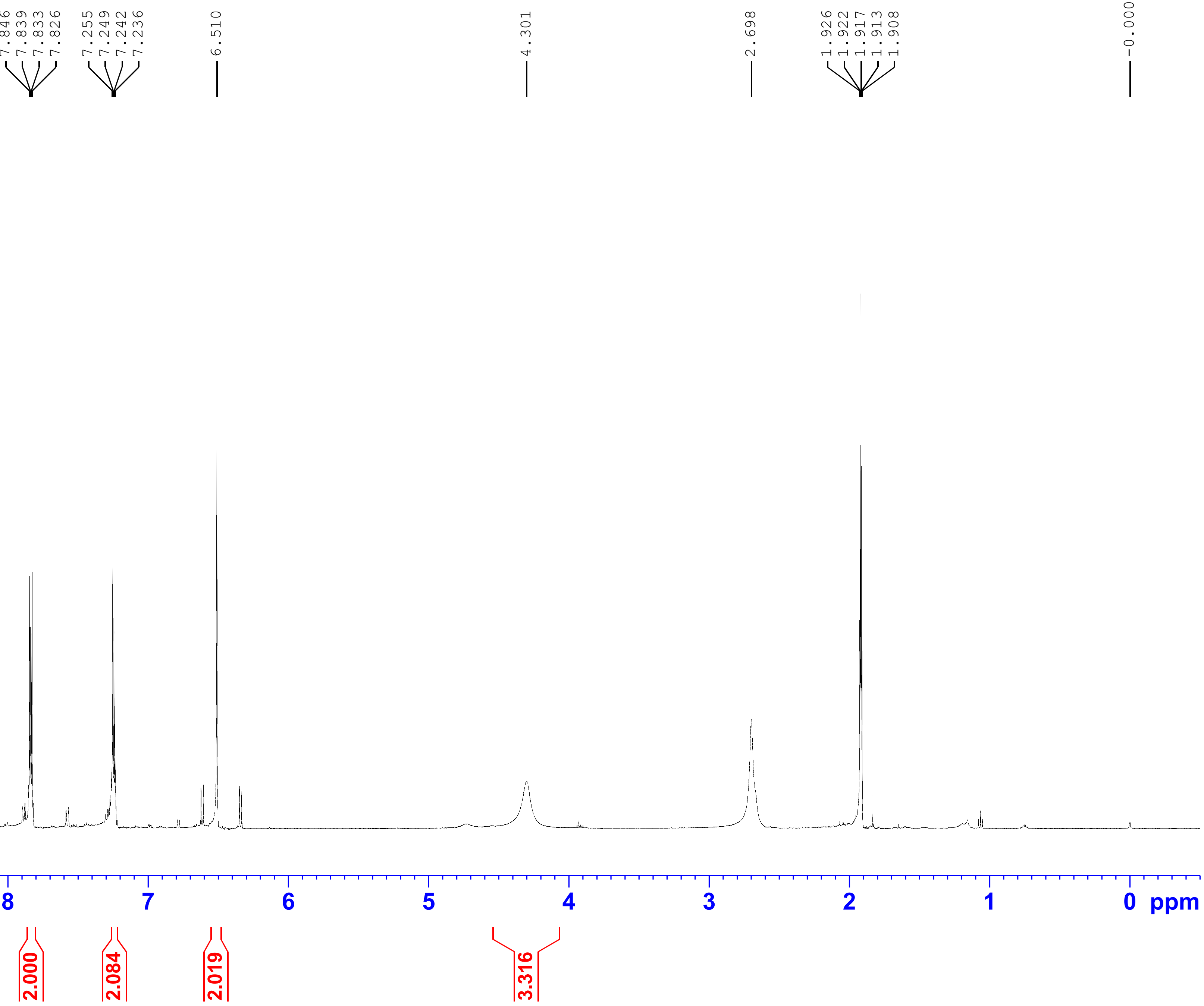

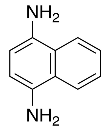

B13

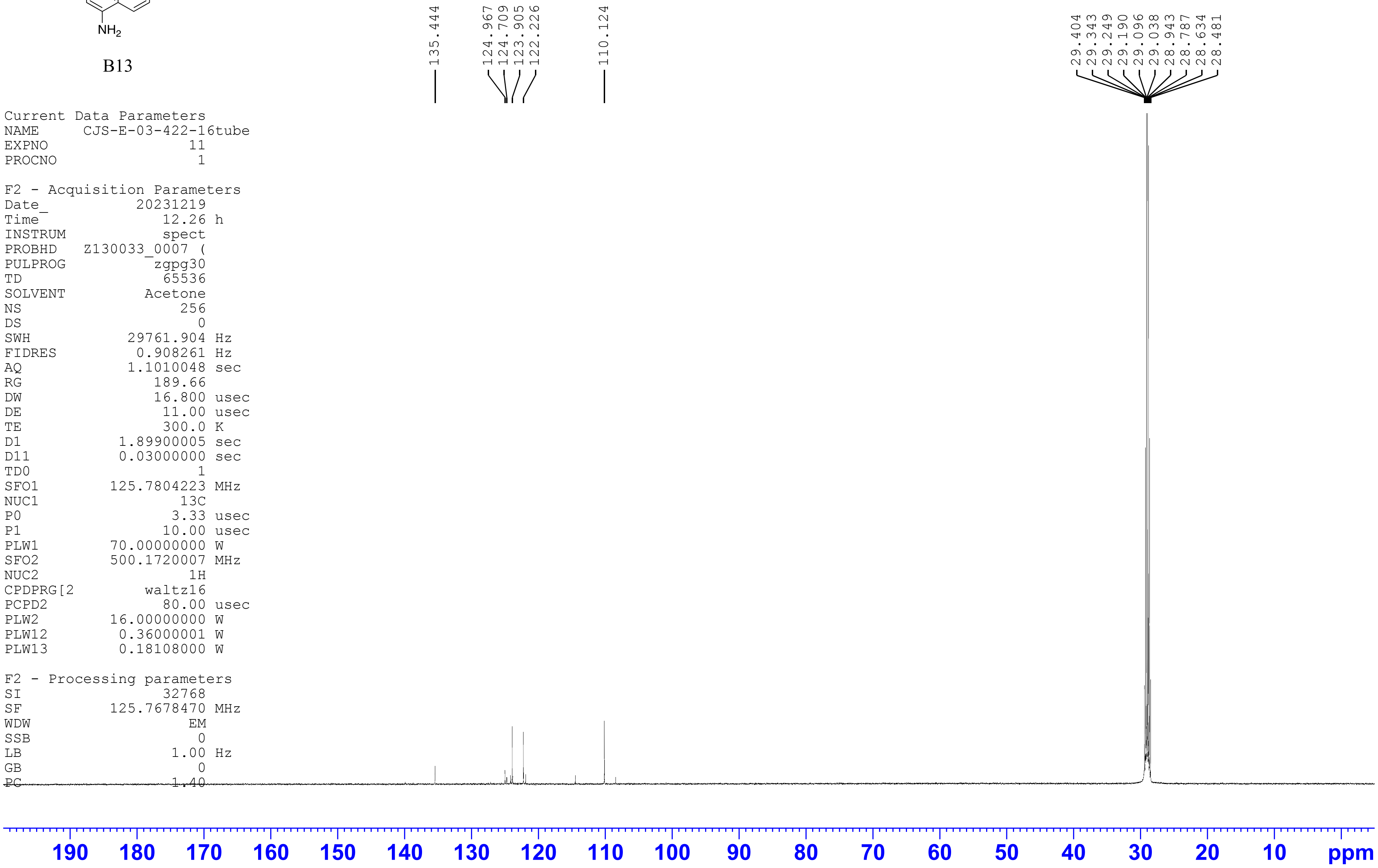

Current Data Parameters
NAME CJS-E-03-422-16tube
EXPNO 11
PROCNO 1

F2 - Acquisition Parameters
Date_ 20231219
Time 12.26 h
INSTRUM spect
PROBHD Z130033_0007 (
PULPROG zgpg30
TD 65536
SOLVENT Acetone
NS 256
DS 0
SWH 29761.904 Hz
FIDRES 0.908261 Hz
AQ 1.1010048 sec
RG 189.66
DW 16.800 usec
DE 11.00 usec
TE 300.0 K
D1 1.89900005 sec
D11 0.03000000 sec
TD0 1
SFO1 125.7804223 MHz
NUC1 13C
P0 3.33 usec
P1 10.00 usec
PLW1 70.00000000 W
SFO2 500.1720007 MHz
NUC2 1H
CPDPRG[2 waltz16
PCPD2 80.00 usec
PLW2 16.00000000 W
PLW12 0.36000001 W
PLW13 0.18108000 W

F2 - Processing parameters
SI 32768
SF 125.7678470 MHz
WDW EM
SSB 0
LB 1.00 Hz
GB 0
PC 1.40

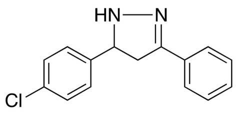

B15

Current Data Parameters

NAME	CJS-E-03-435
EXPNO	10
PROCNO	1

F2 - Acquisition Parameters

Date_	20231226
Time	20.20 h
INSTRUM	spect
PROBHD	Z130033_0007 (
PULPROG	zg30
TD	65536
SOLVENT	CDCl3
NS	1
DS	0
SWH	8012.820 Hz
FIDRES	0.244532 Hz
AQ	4.0894465 sec
RG	31.29
DW	62.400 usec
DE	10.00 usec
TE	300.0 K
D1	1.00000000 sec
TD0	1
SFO1	500.1730010 MHz
NUC1	1H
P0	4.00 usec
P1	12.00 usec
PLW1	16.00000000 W

F2 - Processing parameters

SI	65536
SF	500.1700130 MHz
WDW	EM
SSB	0
LB	0.30 Hz
GB	0
PC	1.00

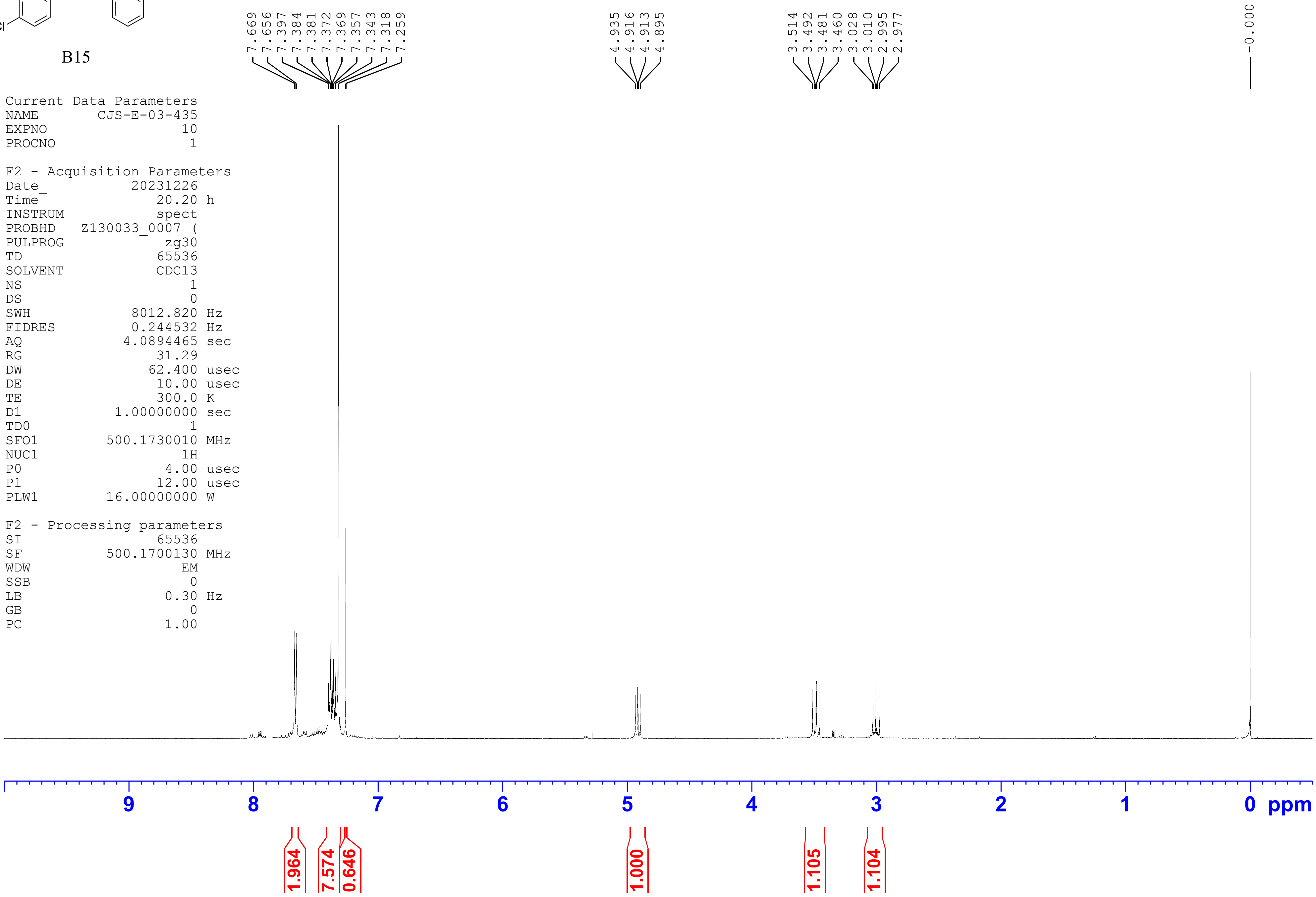

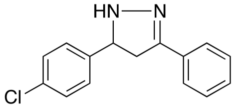

B15

Current Data Parameters
NAME CJS-E-03-435
EXPNO 11
PROCNO 1

F2 - Acquisition Parameters
Date_ 20231226
Time 20.35 h
INSTRUM spect
PROBHD Z130033_0007 (
PULPROG zgpg30
TD 65536
SOLVENT CDCl3
NS 252
DS 0
SWH 29761.904 Hz
FIDRES 0.908261 Hz
AQ 1.1010048 sec
RG 189.66
DW 16.800 usec
DE 11.00 usec
TE 300.0 K
D1 1.89900005 sec
D11 0.03000000 sec
TD0 1
SFO1 125.7804223 MHz
NUC1 13C
P0 3.33 usec
P1 10.00 usec
PLW1 70.00000000 W
SFO2 500.1720007 MHz
NUC2 1H
CPDPRG[2 waltz16
PCPD2 80.00 usec
PLW2 16.00000000 W
PLW12 0.36000001 W
PLW13 0.18108000 W

F2 - Processing parameters
SI 32768
SF 125.7678467 MHz
WDW EM
SSB 0
[illegible]
GB 0
PC 1.40

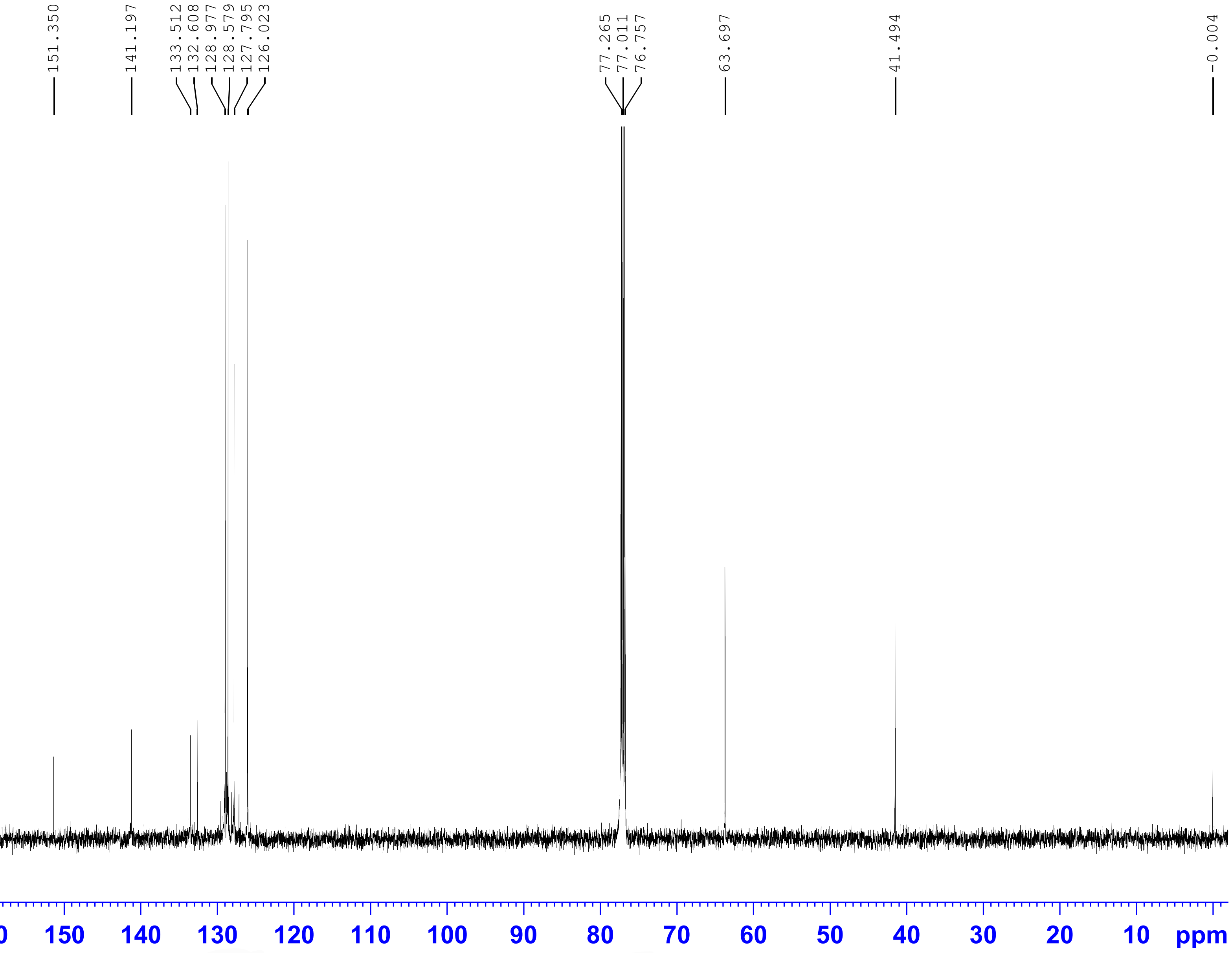

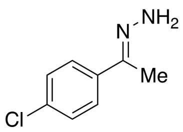

B16

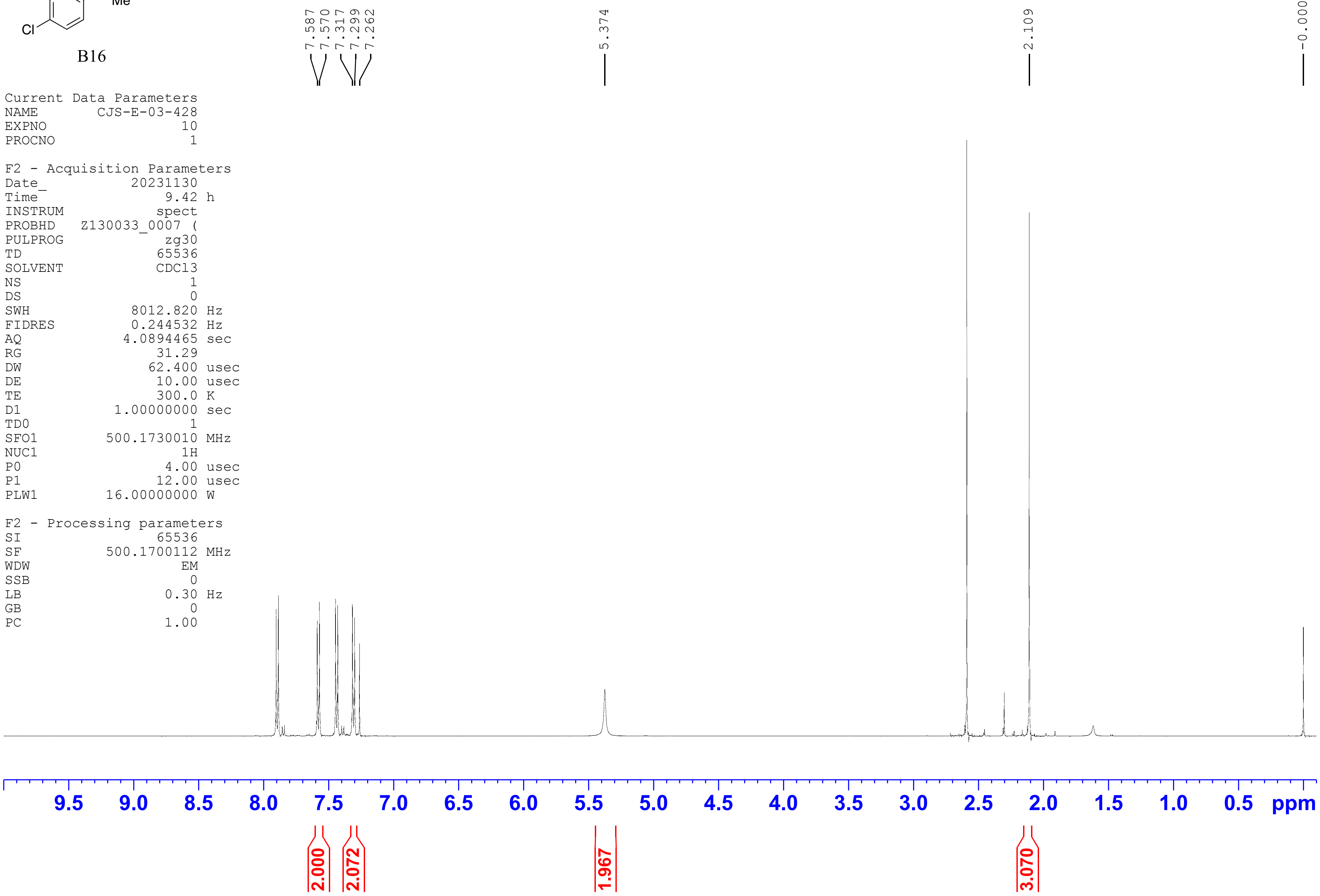


```
Current Data Parameters
NAME        CJS-E-03-428
EXPNO                 10
PROCNO                 1

F2 - Acquisition Parameters
Date_           20231130
Time                9.42 h
INSTRUM            spect
PROBHD   Z130033_0007 (
PULPROG             zg30
TD                 65536
SOLVENT            CDCl3
NS                     1
DS                     0
SWH             8012.820 Hz
FIDRES          0.244532 Hz
AQ             4.0894465 sec
RG                 31.29
DW                62.400 usec
DE                 10.00 usec
TE                 300.0 K
D1            1.00000000 sec
TD0                    1
SFO1         500.1730010 MHz
NUC1                  1H
P0                  4.00 usec
P1                 12.00 usec
PLW1         16.00000000 W

F2 - Processing parameters
SI                 65536
SF           500.1700112 MHz
WDW                   EM
SSB                    0
LB                  0.30 Hz
GB                     0
PC                  1.00
```

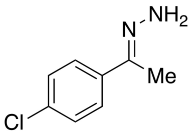

B16

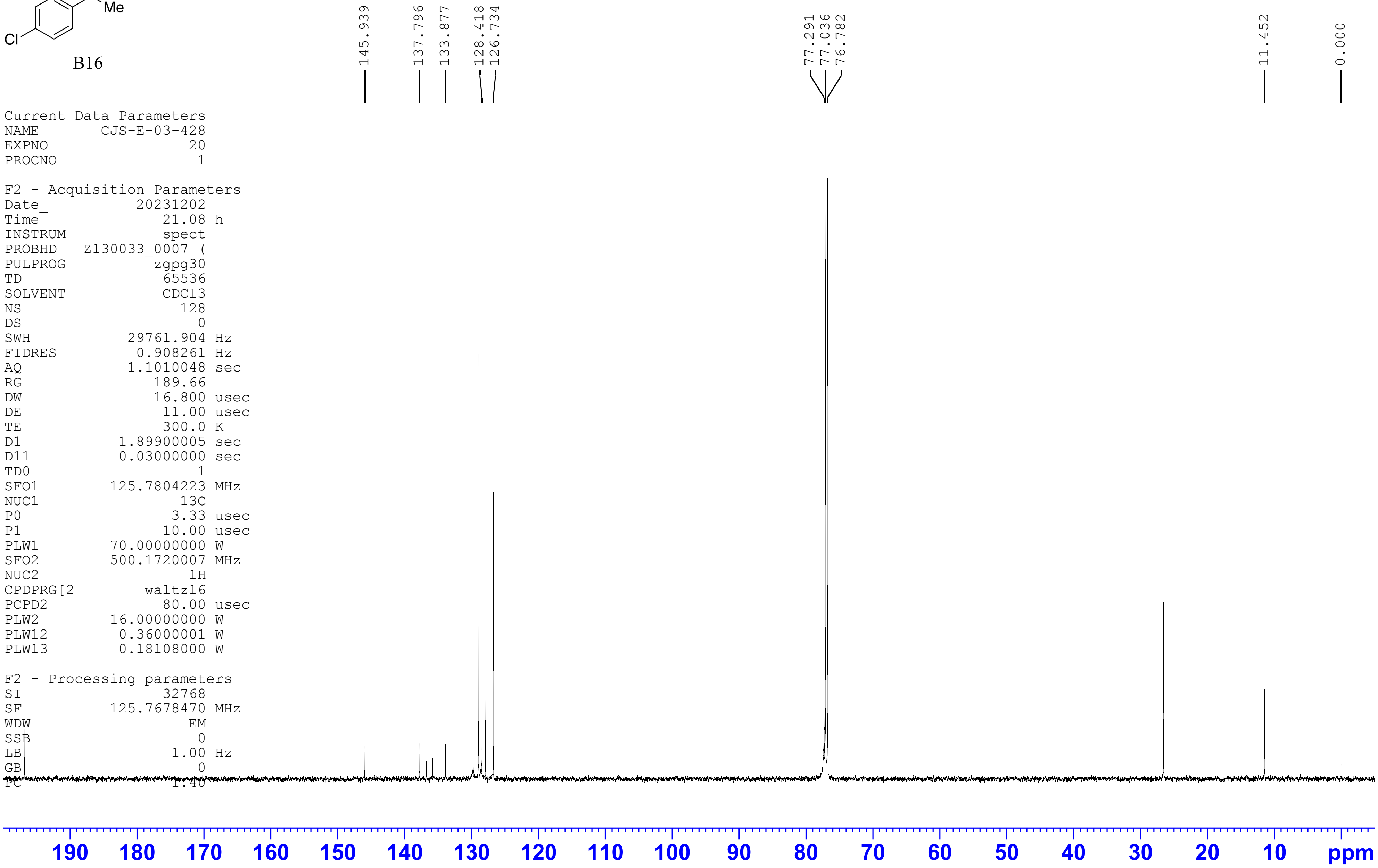


```
Current Data Parameters
NAME       CJS-E-03-428
EXPNO                20
PROCNO                1

F2 - Acquisition Parameters
Date_          20231202
Time              21.08 h
INSTRUM           spect
PROBHD   Z130033_0007 (
PULPROG          zgpg30
TD                65536
SOLVENT           CDCl3
NS                  128
DS                    0
SWH           29761.904 Hz
FIDRES         0.908261 Hz
AQ            1.1010048 sec
RG               189.66
DW               16.800 usec
DE                11.00 usec
TE                300.0 K
D1           1.89900005 sec
D11          0.03000000 sec
TD0                   1
SFO1        125.7804223 MHz
NUC1                13C
P0                 3.33 usec
P1                10.00 usec
PLW1        70.00000000 W
SFO2        500.1720007 MHz
NUC2                 1H
CPDPRG[2        waltz16
PCPD2             80.00 usec
PLW2        16.00000000 W
PLW12        0.36000001 W
PLW13        0.18108000 W

F2 - Processing parameters
SI                32768
SF          125.7678470 MHz
WDW                  EM
SSB                   0
LB                 1.00 Hz
GB                    0
PC                 1.40
```

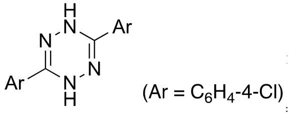

B18

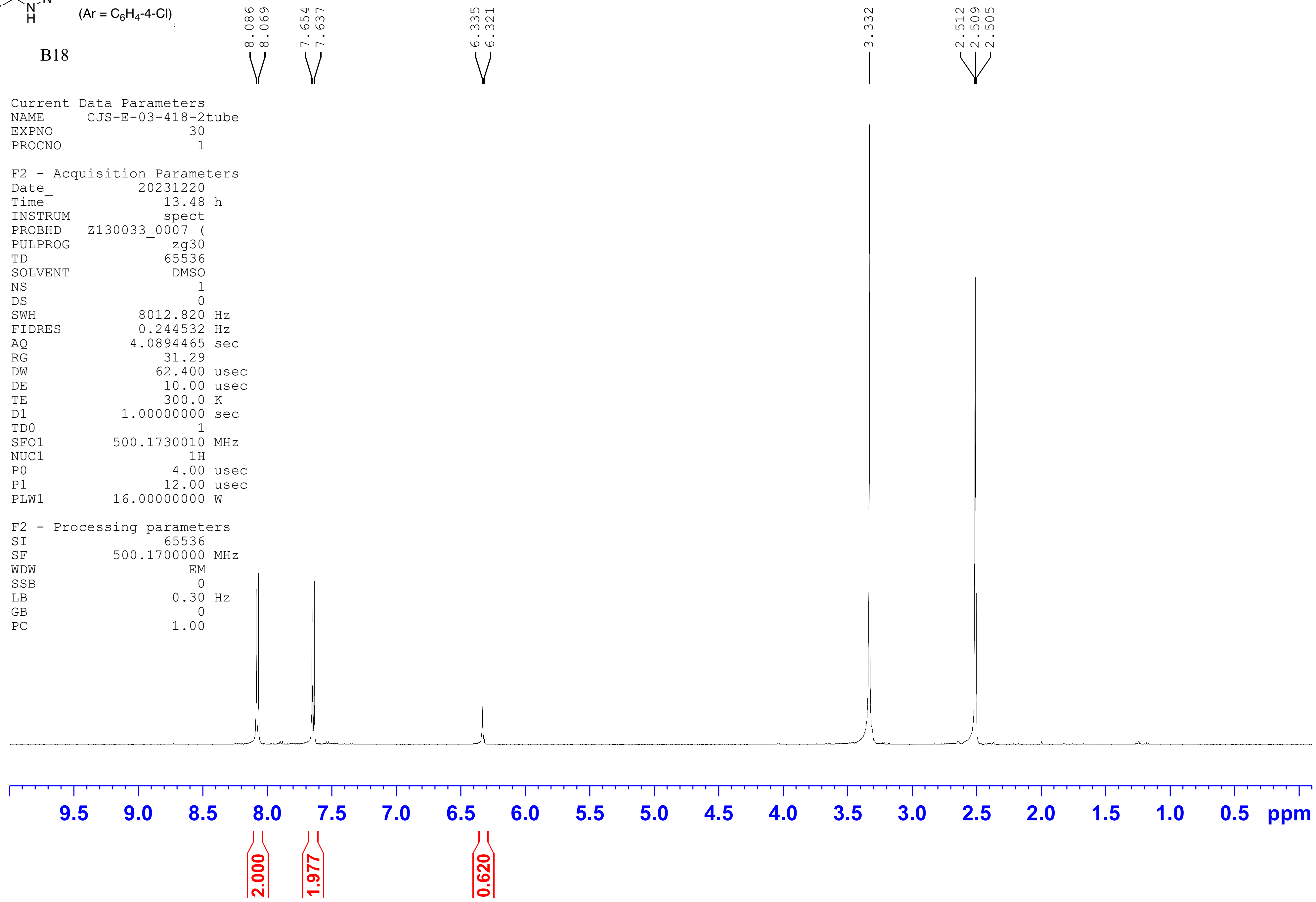

Current Data Parameters
NAME CJS-E-03-418-2tube
EXPNO 30
PROCNO 1

F2 - Acquisition Parameters
Date_ 20231220
Time 13.48 h
INSTRUM spect
PROBHD Z130033_0007 (
PULPROG zg30
TD 65536
SOLVENT DMSO
NS 1
DS 0
SWH 8012.820 Hz
FIDRES 0.244532 Hz
AQ 4.0894465 sec
RG 31.29
DW 62.400 usec
DE 10.00 usec
TE 300.0 K
D1 1.00000000 sec
TD0 1
SFO1 500.1730010 MHz
NUC1 1H
P0 4.00 usec
P1 12.00 usec
PLW1 16.00000000 W

F2 - Processing parameters
SI 65536
SF 500.1700000 MHz
WDW EM
SSB 0
LB 0.30 Hz
GB 0
PC 1.00

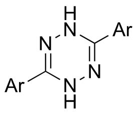

(Ar = C_6H_4-4-Cl)

B18

Current Data Parameters
NAME CJS-E-03-418-2tube
EXPNO 31
PROCNO 1

F2 - Acquisition Parameters
Date_ 20231220
Time 14.00 h
INSTRUM spect
PROBHD Z130033_0007 (
PULPROG zgpg30
TD 65536
SOLVENT DMSO
NS 200
DS 0
SWH 29761.904 Hz
FIDRES 0.908261 Hz
AQ 1.1010048 sec
RG 189.66
DW 16.800 usec
DE 11.00 usec
TE 300.0 K
D1 1.89900005 sec
D11 0.03000000 sec
TD0 1
SFO1 125.7804223 MHz
NUC1 13C
P0 3.33 usec
P1 10.00 usec
PLW1 70.00000000 W
SFO2 500.1720007 MHz
NUC2 1H
CPDPRG[2 waltz16
PCPD2 80.00 usec
PLW2 16.00000000 W
PLW12 0.36000001 W
PLW13 0.18108000 W

F2 - Processing parameters
SI 32768
SF 125.7678470 MHz
WDW EM
SSB 0
LB 1.00 Hz
GB 0
PC 1.40

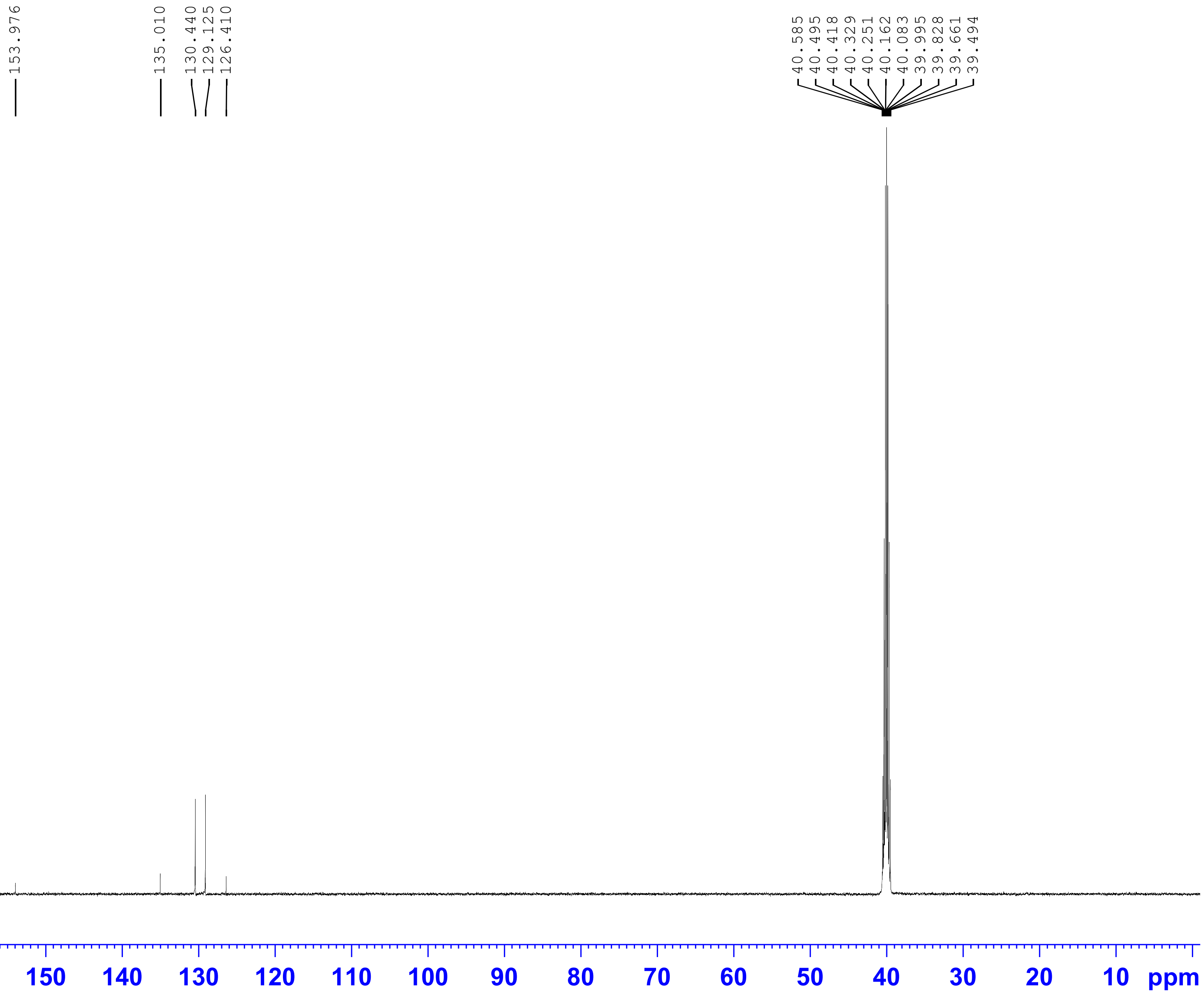

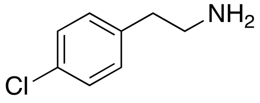

B21

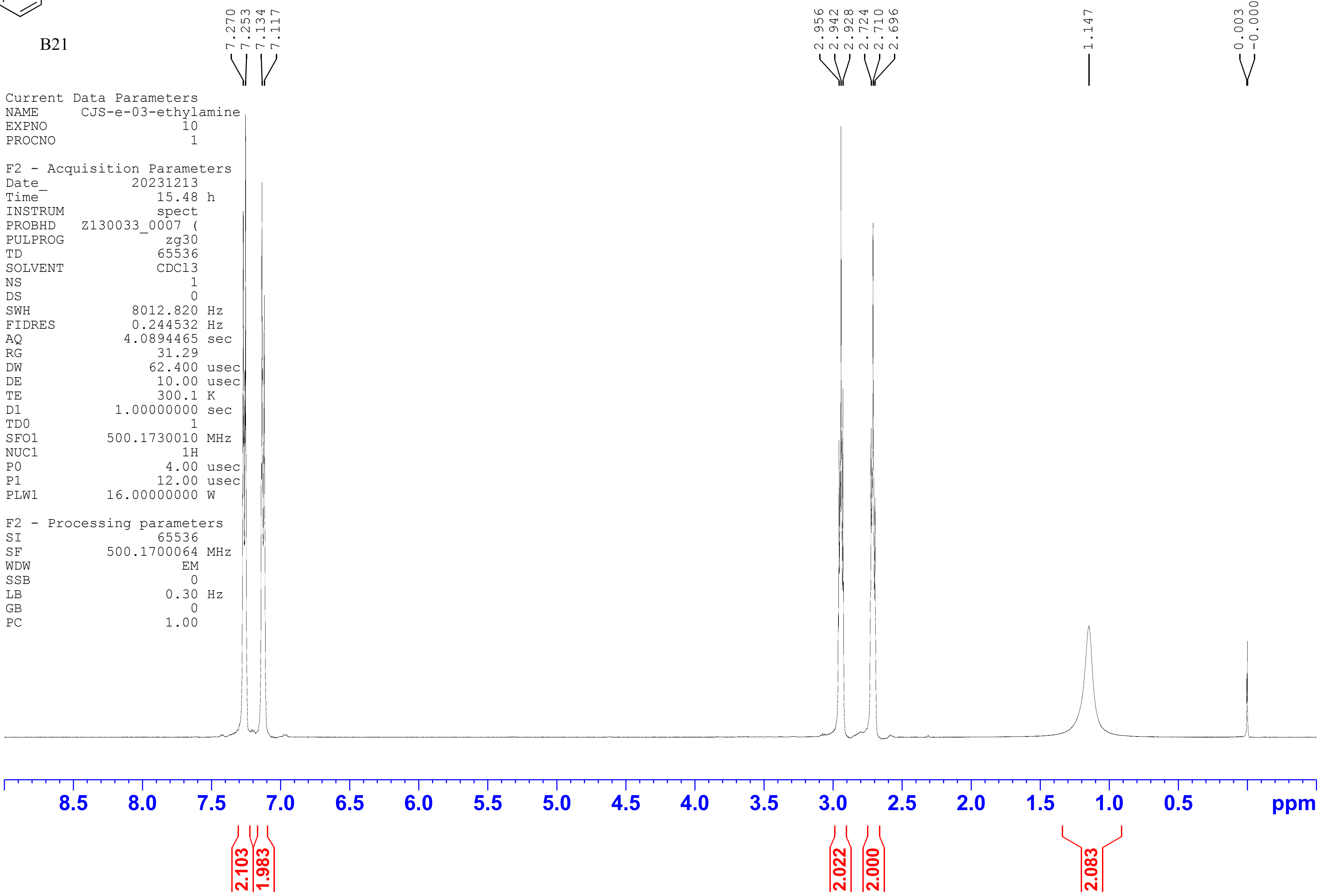


```
Current Data Parameters
NAME     CJS-e-03-ethylamine
EXPNO                  10
PROCNO                  1

F2 - Acquisition Parameters
Date_            20231213
Time                15.48 h
INSTRUM             spect
PROBHD   Z130033_0007 (
PULPROG              zg30
TD                  65536
SOLVENT             CDCl3
NS                      1
DS                      0
SWH              8012.820 Hz
FIDRES           0.244532 Hz
AQ              4.0894465 sec
RG                  31.29
DW                 62.400 usec
DE                  10.00 usec
TE                  300.1 K
D1             1.00000000 sec
TD0                     1
SFO1          500.1730010 MHz
NUC1                   1H
P0                   4.00 usec
P1                  12.00 usec
PLW1          16.00000000 W

F2 - Processing parameters
SI                  65536
SF            500.1700064 MHz
WDW                    EM
SSB                     0
LB                   0.30 Hz
GB                      0
PC                   1.00
```

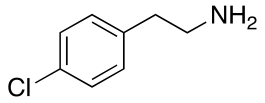

B21

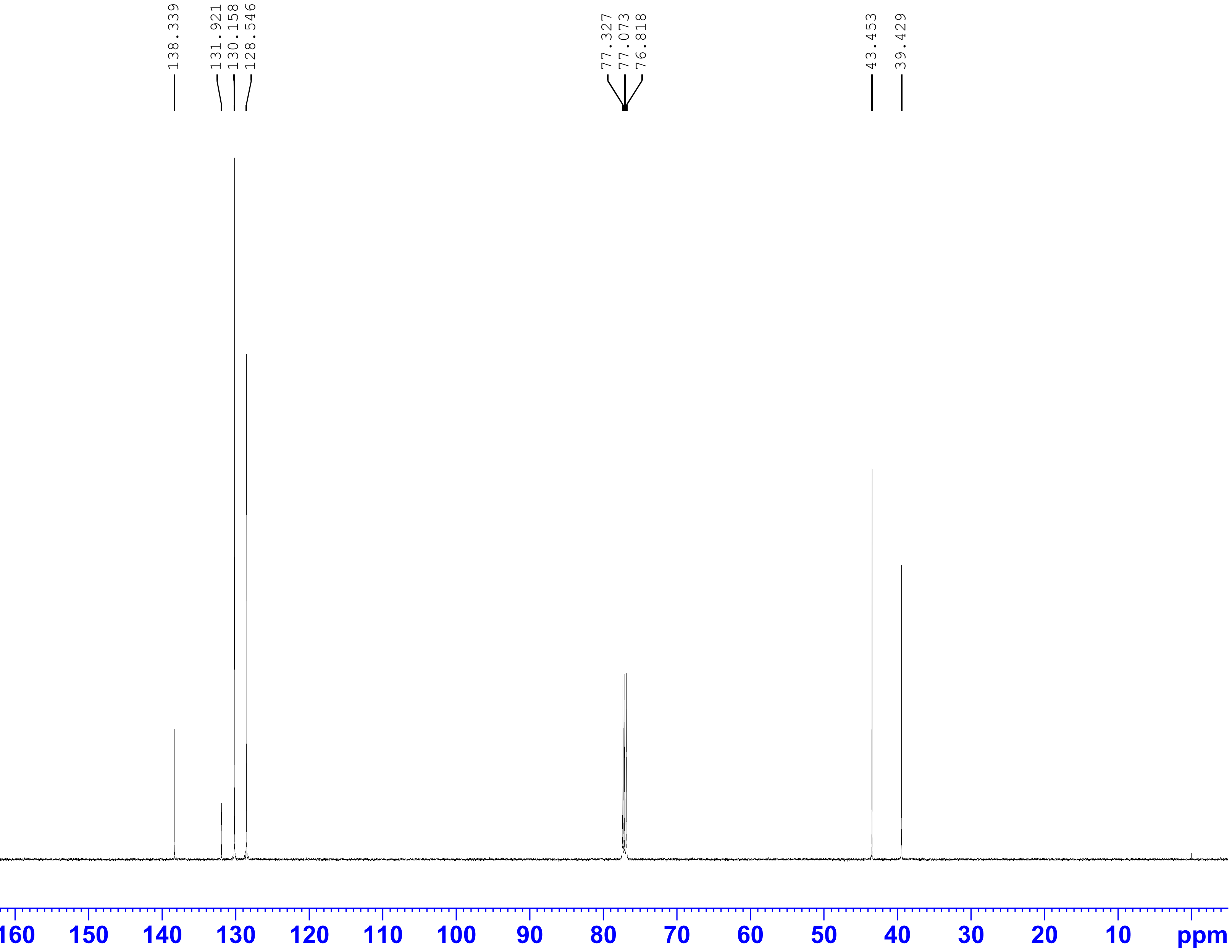

Current Data Parameters
NAME CJS-e-03-ethylamine
EXPNO 11
PROCNO 1

F2 - Acquisition Parameters
Date_ 20231213
Time 15.56 h
INSTRUM spect
PROBHD Z130033_0007 (
PULPROG zgpg30
TD 65536
SOLVENT CDCl3
NS 128
DS 0
SWH 29761.904 Hz
FIDRES 0.908261 Hz
AQ 1.1010048 sec
RG 189.66
DW 16.800 usec
DE 11.00 usec
TE 300.0 K
D1 1.89900005 sec
D11 0.03000000 sec
TD0 1
SFO1 125.7804223 MHz
NUC1 13C
P0 3.33 usec
P1 10.00 usec
PLW1 70.00000000 W
SFO2 500.1720007 MHz
NUC2 1H
CPDPRG[2 waltz16
PCPD2 80.00 usec
PLW2 16.00000000 W
PLW12 0.36000001 W
PLW13 0.18108000 W

F2 - Processing parameters
SI 32768
SF 125.7678470 MHz
WDW EM
SSB 0
LB 1.00 Hz
GB 0
PC 1.40

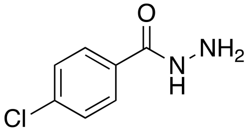

B24

Current Data Parameters	
NAME	CJS-E-03-145-2spot
EXPNO	10
PROCNO	1

F2 - Acquisition Parameters	
Date_	20220804
Time	10.35 h
INSTRUM	spect
PROBHD	Z130033_0007 (
PULPROG	zg30
TD	65536
SOLVENT	CDCl3
NS	1
DS	0
SWH	8012.820 Hz
FIDRES	0.244532 Hz
AQ	4.0894465 sec
RG	31.29
DW	62.400 usec
DE	10.00 usec
TE	300.0 K
D1	1.00000000 sec
TD0	1
SFO1	500.1730010 MHz
NUC1	1H
P0	4.00 usec
P1	12.00 usec
PLW1	13.50000000 W

F2 - Processing parameters	
SI	65536
SF	500.1700115 MHz
WDW	EM
SSB	0
LB	0.30 Hz
GB	0
PC	1.00

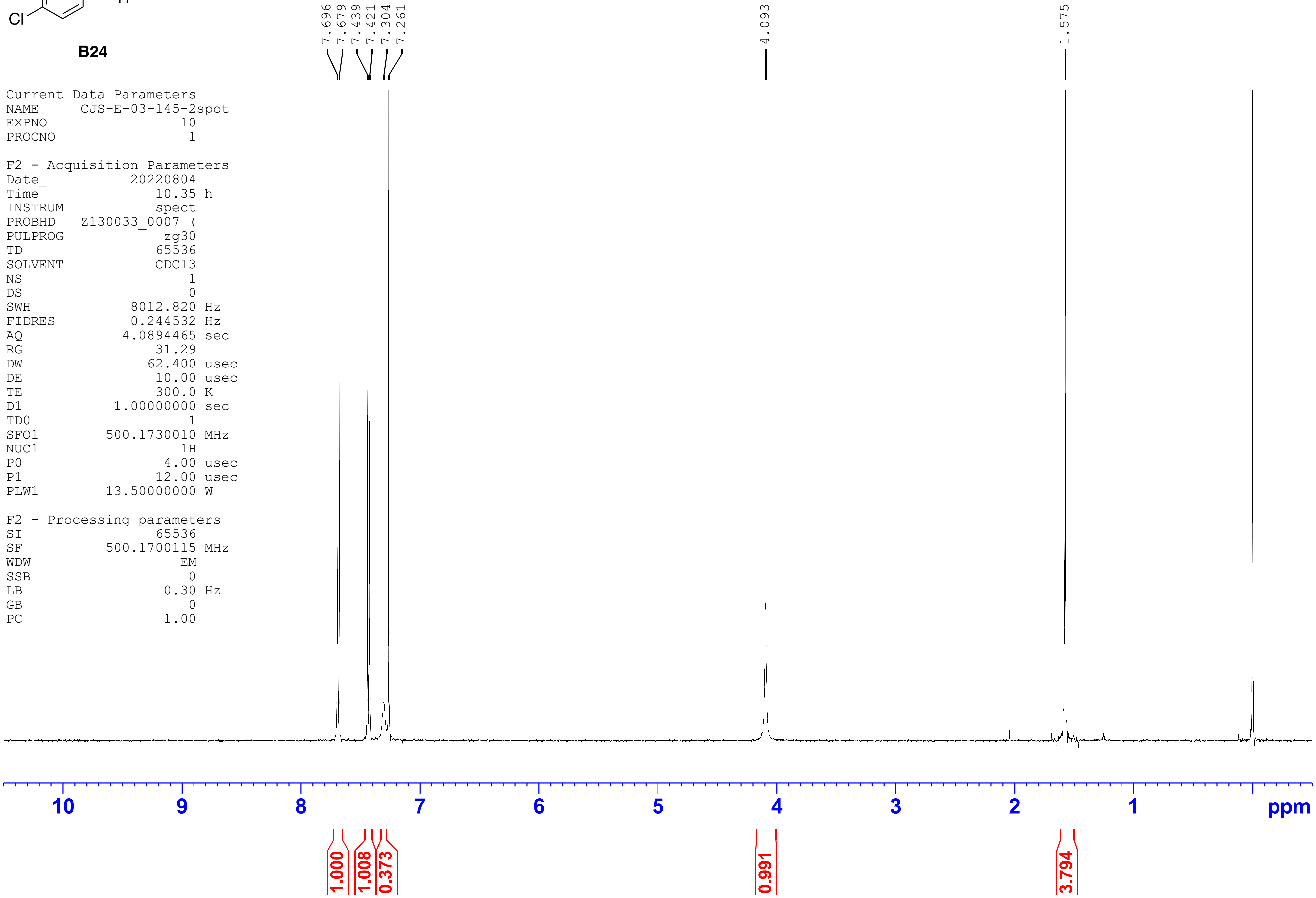

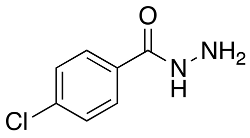

B24

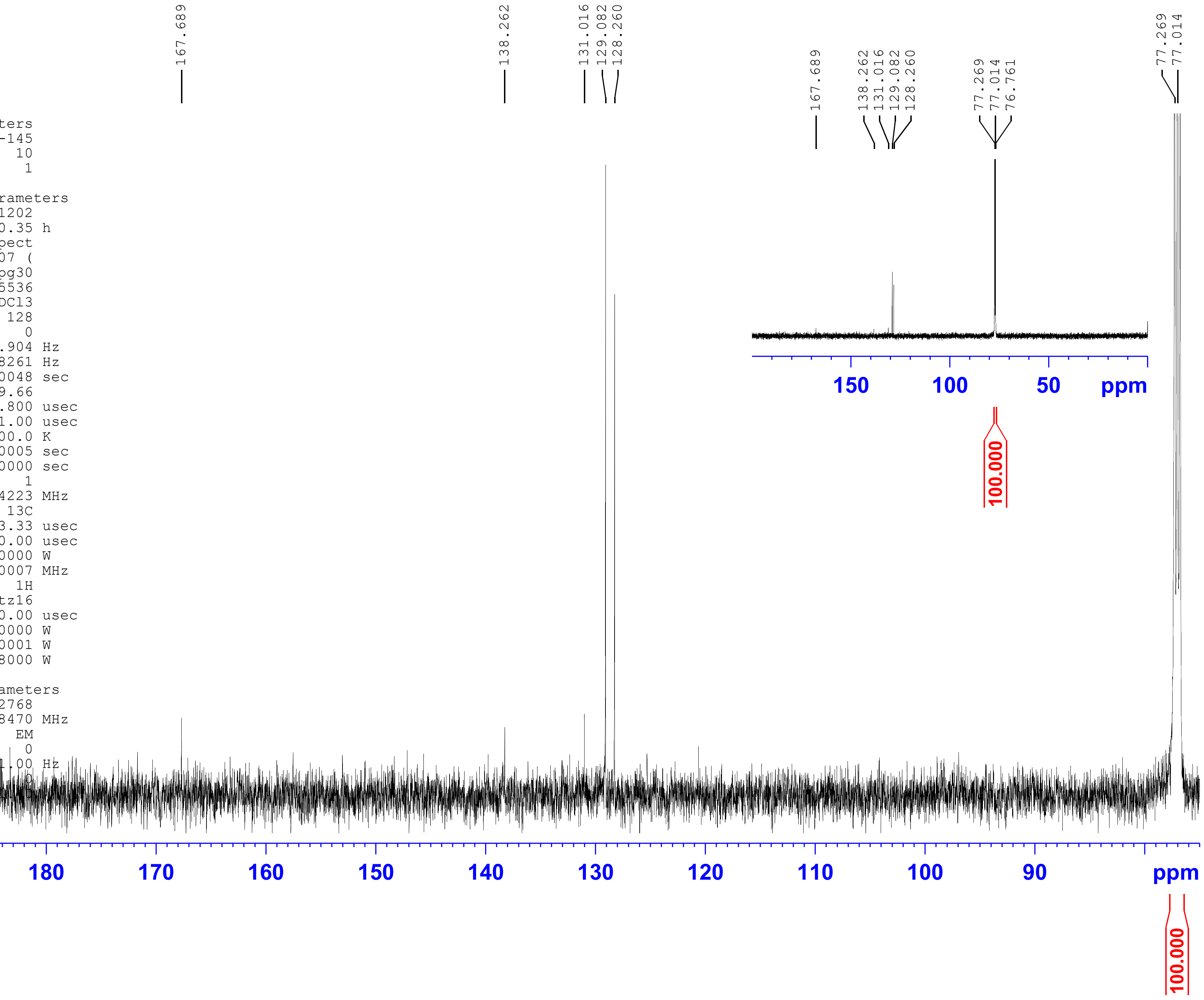


```
Current Data Parameters
NAME          CJS-E-03-145
EXPNO                   10
PROCNO                   1

F2 - Acquisition Parameters
Date_             20231202
Time                 20.35 h
INSTRUM              spect
PROBHD    Z130033_0007 (
PULPROG            zgpg30
TD                   65536
SOLVENT              CDCl3
NS                     128
DS                       0
SWH              29761.904 Hz
FIDRES            0.908261 Hz
AQ               1.1010048 sec
RG                  189.66
DW                  16.800 usec
DE                   11.00 usec
TE                   300.0 K
D1              1.89900005 sec
D11             0.03000000 sec
TD0                      1
SFO1           125.7804223 MHz
NUC1                   13C
P0                    3.33 usec
P1                   10.00 usec
PLW1           70.00000000 W
SFO2           500.1720007 MHz
NUC2                    1H
CPDPRG[2           waltz16
PCPD2                80.00 usec
PLW2           16.00000000 W
PLW12           0.36000001 W
PLW13           0.18108000 W

F2 - Processing parameters
SI                   32768
SF             125.7678470 MHz
WDW                     EM
SSB                      0
LB                    1.00 Hz
GB                       0
PC              [illegible]
```
